# A systematic review of consumer preference for e-cigarette attributes: Flavor, nicotine strength, and type

**DOI:** 10.1371/journal.pone.0194145

**Published:** 2018-03-15

**Authors:** Samane Zare, Mehdi Nemati, Yuqing Zheng

**Affiliations:** Department of Agricultural Economics, University of Kentucky, Lexington, Kentucky, United States of America; Ohio State University, UNITED STATES

## Abstract

**Objective:**

Systematic review of research examining consumer preference for the main electronic cigarette (e-cigarette) attributes namely flavor, nicotine strength, and type.

**Method:**

A systematic search of peer-reviewed articles resulted in a pool of 12,933 articles. We included only articles that meet all the selection criteria: (1) peer-reviewed, (2) written in English, and (3) addressed consumer preference for one or more of the e-cigarette attributes including flavor, strength, and type.

**Results:**

66 articles met the inclusion criteria for this review. Consumers preferred flavored e-cigarettes, and such preference varied with age groups and smoking status. We also found that several flavors were associated with decreased harm perception while tobacco flavor was associated with increased harm perception. In addition, some flavor chemicals and sweeteners used in e-cigarettes could be of toxicological concern. Finally, consumer preference for nicotine strength and types depended on smoking status, e-cigarette use history, and gender.

**Conclusion:**

Adolescents could consider flavor the most important factor trying e-cigarettes and were more likely to initiate vaping through flavored e-cigarettes. Young adults overall preferred sweet, menthol, and cherry flavors, while non-smokers in particular preferred coffee and menthol flavors. Adults in general also preferred sweet flavors (though smokers like tobacco flavor the most) and disliked flavors that elicit bitterness or harshness. In terms of whether flavored e-cigarettes assisted quitting smoking, we found inconclusive evidence. E-cigarette users likely initiated use with a cigarette like product and transitioned to an advanced system with more features. Non-smokers and inexperienced e-cigarettes users tended to prefer no nicotine or low nicotine e-cigarettes while smokers and experienced e-cigarettes users preferred medium and high nicotine e-cigarettes. Weak evidence exists regarding a positive interaction between menthol flavor and nicotine strength.

## Introduction

Electronic cigarettes (e-cigarettes) have been increasingly popular among youth [1] and adults [2, 3]. In 2014, the use of the e-cigarette surpassed cigarette usage in adolescents for the first time in history [4]. Unlike e-cigarettes, cigarettes have been the subject of heavy tobacco control policies that target specific product attributes. For example, the U.S. Food and Drug Administration (FDA) has authority to regulate tobacco products, such as setting standards for cigarette nicotine and tar levels, banning flavored cigarettes except for menthol, and requiring cigarettes be sold in packs of at least twenty. Beginning in mid-2016, FDA extended their regulatory authority to e-cigarettes and has worked to level the playing field with cigarettes. One example is a mandatory nicotine and tobacco warning statement on e-cigarette product packages targeting a start date in 2018. However, they extended the deadline to 2022 for the vaping industry to comply with new FDA guidelines [5].

FDA also can regulate e-cigarette attributes. E-cigarettes have a variety of characterizing attributes, such as flavor, nicotine strength, type (also known as form), price, health warning, brand, battery life, e-liquid size, and device weight. Hundreds of e-cigarette flavors exist, including tobacco, menthol, fruit, and coffee, etc. E-cigarettes are also sold in different types, such as disposable versus refillable, and cigarette like (cigalike) versus advanced systems with more powerful batteries, a manual button, and a larger choice of liquid flavors. Strength is measured by the amount of nicotine in milligrams per milliliter of the e-liquid. Given the regulatory shift to the FDA and other potential policy changes at the local/state level (e.g., San Francisco is proposing to ban the sales of all flavored tobacco products including e-cigarettes [6]), there is a critical need from a research perspective to understand how consumers perceive various e-cigarette attributes, which becomes the focus of this study.

Review studies on consumer preference for tobacco product attributes are largely limited to flavors, focusing on either preference for flavors that can be used in tobacco products [7] or flavored tobacco products in general [8]. Specifically, one study examined the available evidence of children and adults’ preferences for flavors that can be used in tobacco products. Their study, not specifically addressing preferences for e-cigarettes flavors, found that infants and children had a stronger preference for sweet and salt compared with adults [7]. Another study reviewed 32 studies on the use of and attitudes toward flavored tobacco products, of which only four studies are related to e-cigarettes [8]. A more recent study focused on non-menthol flavors in tobacco products [9]. Our study focuses on flavor, strength, and type as three key e-cigarette attributes, where the literature is mostly concentrated (e.g., we found no study addressing e-liquid size). In addition, results on flavor are classified by age cohorts, and categorized based on the contribution to smoke cessation, toxicity, and harm perception. These results will provide information that can be used to determine what regulations might be needed.

## Materials and methods

### Search strategy, study selection, and data extraction

We performed a systematic literature review using the search terms (“electronic cigarettes”, “e-cigarettes”, “electronic nicotine delivery systems”, “E-cig”, and “E-cigarette”) in five databases (PubMed, MEDLINE, Web of Science, PsycINFO, and CINAHL Plus) for publications studying consumer preference for e-cigarette attributes. Our search strategy used the Boolean search strategy to identify the potential studies for this review study only using one level based on the keywords mentioned above. Avoiding using further search filters is the advantage of our study, which reduces the risk of missing relevant studies. Also, for the same reason, we applied the same search terms to 11 journals that publish tobacco-related studies in addition to the five databases. These journals include Tobacco Control, Nicotine & Tobacco Research, Addictive Behaviors, Addiction, Drug, and Alcohol Dependence, Health Education, Drug & Alcohol Review, Journal of Pediatrics, American Journal of Preventive Medicine, International Journal of Public Health, and Preventive Medicine Reports.

Studies examining humans of any age, race/ethnicity, gender, were eligible for this review. We began the search on October 1^st^, 2016 and finished the process on January 8^th^, 2018. We searched without imposing restrictions on date or year, locations, study design, study aim, or inclusion/exclusion criteria. Using the search procedure, we retrieved a pool of 12,933 articles with the title and abstract related to e-cigarettes.

Based on this pool, two reviewers screened titles and abstracts using the following inclusion criteria: (1) peer-reviewed and published papers, (2) written in English, (3) relevant to consumer preference for e-cigarettes attributes. Therefore, working papers, editorial comments and letters, and news articles were excluded. There are no temporal or geographical restrictions, and all international, national or subnational populations were included. Next based on our original pool and these criteria, following a previous study method [10] the two reviewers were also assigned to review 10% of randomly selected articles that were excluded by each other. Disagreements at each of these steps were resolved through discussion between the two reviewers, and with a third reviewer as required.

## Results

**Fig 1:**
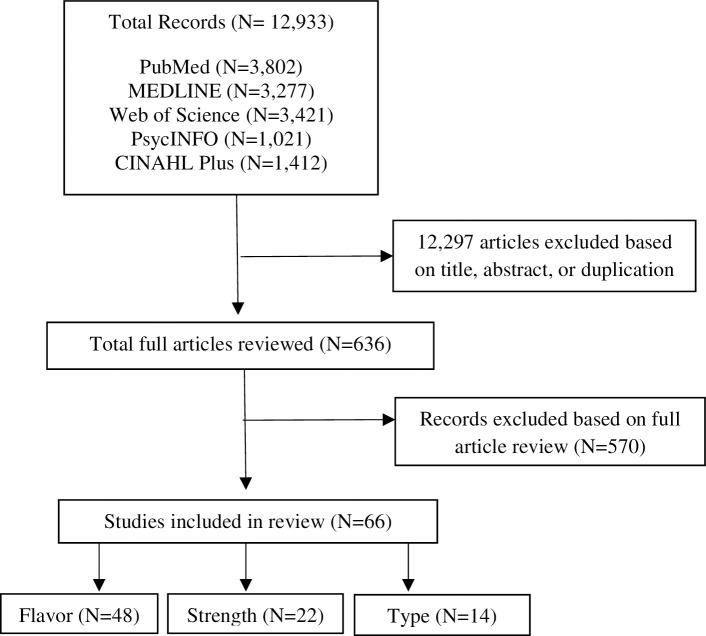
Studies screened and selected for inclusion in the review of consumer preferences for e-cigarette attributes.

We screened 12,933 references and studied the full text of a final 636 articles. All these 636 articles were published from 2010 through 2018, reflecting the popularity of research on e-cigarettes in recent years. Fig 1 describes the search process and the number of articles excluded in each step. After reviewing titles and abstracts, we excluded duplicates, irrelevant articles, editorials, and working papers. Next, full articles were reviewed and 570 articles were excluded from this review because they did not meet our inclusion criteria. For this study, we reviewed 66 articles, of which 13 were published in 2017, and 34 were published in 2016. These articles are divided into three main groups: flavor (48 studies), strength (22 studies), and type (14 studies). Some studies investigated consumer preference for more than one e-cigarette attributes. A full list of included and excluded articles and exclusion reasons is presented in S1 Appendix. PRISMA Checklist is presented in S1 Table.

In Table 1, we provide a list of all the 66 articles, a short description of the sample (e.g., age, sample size, [cigarette] smokers, [e-cigarette] vapers, and location of the study if it is done in the countries other than the United States), summary of the findings, classification of data type (e.g., experiment, focus group, and survey), and finally methods (descriptive, regression, etc.). In this section, we summarize the findings in the literature regarding consumer preference for the three attributes (flavor, nicotine strength, and type).

**Table 1:** Summary of peer-reviewed literature on consumer preference for e-cigarette attributes.

Lead Author & Year	Sample Description	Findings	Data Type/Method ^a^
Audrain, 2016 [11]	Cigarette smokers (n = 32, age 18–30)	Flavoring enhances the experience of the vaping value of e-cigsb with nicotine for cigarette smokers.	Experiment, 2
Baweja, 2016 [12]	E-cig users (n = 200, age 30–50)	Tank feed e-cig liquid with a variety of flavors is preferable for around 60% of experienced e-cig users.	Online survey, 1
Berg, 2016 [13]	Never, current, and former smokers (n = 1567, age 18–34)	Current smokers prefer various flavors; however, fruit flavors are more desirable.	Online survey, 1
Bold, 2016 [14]	Middle and high school students (n = 340)	After curiosity, the flavor is the most important factor in the decision to try e-cigs.	Longitudinal surveys, 2
Bonhomme, 2016 [15]	Adults (n = 75,233, age > 18)	Preferences for e-cig flavors are fruit, menthol/mint, and candy, chocolate, and other sweet flavors in descending order.	2013–2014 NATS, 1
Browne, 2018 [16]	E-cig users (n = 436, age 17–88)	Older and female e-cig users prefer a low power, higher nicotine-concentration style of vaping.	Internet discussion forums, 2
Camenga, 2017 [17]	Current and former smokers who are e-cig ever-user (n = 189, mean age 18)	Menthol and combination of two or more flavors mixed together are preferred flavors.	Survey, 2
Chen, 2016 [18]	E-cig users who ever smoked (n = 923, age > 17)	Open systems are more likely used by former smokers and more likely to be used daily than a closed system. Users intend to reduce their intake but with higher nicotine concentration e-cigs.	Online survey, 1
Choi, 2012 [19]	Young adult tobacco users and non-users (n = 66, age 18–26)	Flavors contributed to positive perceptions of new e-cig products.	Focus group, 1
Clarke, 2017 [20]	Adolescents (n = 256, age 16–19) in the U.K.	Flavored e-cigs are more appealing than non-flavored ones, and tobacco flavor was less favorable compared with other flavors.	Survey, 1&2
Cooper, 2016 [21]	Current e-cig users (n = 50, age 19–61)	Trying different flavors is one primary reason for using e-cigs.	Interview, 1
Czoli, 2016 [22]	Cigarette smokers and non-smokers (n = 915, age > 16) in Canada	New vapers prefer menthol or cherry flavors with low or medium nicotine content over coffee flavored e-cigs with none or high nicotine content.	Online survey, 2
Dai, 2016 [23]	Middle and high school students (n = 21,491)	Flavored e-cig use is associated with a higher initiation rate of cigarette use, a lower intention rate of quitting tobacco use, and a lower prevalence use of the perception of tobacco’s danger.	2014 NYTSc, 1
Dawkins, 2013 [24]	Primarily ex- and current cigarette smokers (n = 1,347, mean age 43) in 33 countries	Most popular: tobacco, fruit, menthol (flavor); 18mg, 11mg, 24mg (strength); tank use, tornado tank Ego-c, tornado tank (type). No significant differences between ex and current smokers for any demographic variable or country of origin.	Online survey, 1
Dawkins, 2015 [25]	Smokers (n = 97, age mean 26) in the U.K.	Second-generation devices are more satisfying.	Survey, 2
EL-Hellani, 2018 [26]	27 e-cig products from 10 top brands	Nicotine emissions vary widely from 0.27 to 2.91 mg/15 puffs.	Lab test, 2
Elkalmi, 2016 [27]	General population (n = 277, age > 17) in Malaysia	Variety of flavors are preferable for e-cig users.	Survey, 1
Etter, 2015 [28]	Former smokers who are e-cig users (n = 374, age > 18)	Refillable e-cigs with a high level of nicotine provides stronger attenuation of craving for tobacco.	Online Survey, 1
Etter, 2016a [29]	E-cig users dedicated to quit smoking (n = 98, age > 18) in Switzerland, France, or the U.S.	E-cig users decreased the concentration of nicotine in their e-liquids but increased their consumption in order to compensate.	Online survey, 1
Etter, 2016b [30]	Current e-cig users (n = 2,807, age > 18) in several countries	Refillable e-cigs are more effective in smoke reduction and cessation.	Online survey, 1
Feirman,2015 [8]	Review of 32 tobacco-related studies	Consumers prefer flavored tobacco products, and these products are more common for youth.	Review study, 1
Ford, 2016 [31]	Adolescents (n = 1,205, age 11–16) in the U.K.	E-cigs were perceived as harmful (moderated by product flavors). Fruit and sweet flavors were more likely to be tried by never smoker than smokers trying to quit.	2014 YTPSd 1
Giovenco, 2014 [32]	Current and former cigarette smokers (n = 2,136, age > = 18)	Established users prefer rechargeable e-cigs over disposable ones.	Online survey, 2
Goldenson, 2016 [33]	Young adult e-cig users (n = 20, age 19–34)	Sweet-flavored solutions produced greater appeal than other flavors and nicotine increases throat hit.	Experiment, 1 & 2
Harrell, 2017 [34]	General population (n = 15,440, age> = 12)	Most of e-cig users initiate with flavored e-cigs, and never smokers and former smokers start with non-tobacco flavored e-cigs while dual users start with tobacco flavors.	TATAMSe, M-PACT^f^ & TPRPS^g^, 1
Hoffman, 2016 [7]	Review of 59 studies	The flavoring in tobacco products impacts use and initiation for young adults while product switching or dual use for adults.	Review study, 1
Huang, 2017 [9]	Review of 40 tobacco flavor-related studies	Flavors play an important role in the initiation and continue to use and decrease the initiation to quit tobacco products.	Review study, 1
Hutzler, 2014 [35]	28 e-liquids from 7 manufacturers in Germany	141 flavor chemicals identified in one or more of the products and about 80% of e-liquids contained at least one flavor chemical.	Lab test, 1
Kim, 2016 [36]	Young adult and adult e-cig users (n = 31, age 22–44)	Sweetness and coolness are preferred (bitterness and harshness are not).	Experiment, 1&2
Kinnunen, 2016 [37]	Adolescents (n = 10,233, age 12–18) in Finland	Ever smokers prefer e-cig with liquid containing nicotine while non-smokers prefer liquid without nicotine.	2013 & 2015 AHLSh 1&2
Kinouani, 2017 [38]	French-speaking students (n = 2,720, age > = 18) mostly in France	Flavors are ranked as the third most important reason for trying e-cigarettes, after curiosity and being offered by someone to try.	Survey, 1&2
Kistler, 2017 [39]	E-cig users (n = 34, age 18–80)	Among e-cig features, women pay more attention to flavor and young adult to the modifiability.	Interviews, 1
Kong, 2015 [40]	Students (n = 1,302, age 12–22)	Appealing flavors is the most important factor for trying e-cig after curiosity.	Focus group & survey, 1
Krishnan-Sarin, 2015 [41]	High and middle school students (n = 4,780, age 11–21)	Rechargeable e-cigs with sweet flavors is most popular. Current cigarette smokers initiate e-cigs with nicotine containing and ever and never cigarette smokers initiate e-cigs without nicotine.	Survey, 2
Krishnan-Sarin, 2017 [42]	e-cig users (n = 60, age 16–20)	For youth, menthol increases the positive rewarding effects of high nicotine strength of e-cigs.	Experiment, 1
Laverty, 2016 [43]	Ever tobacco and e-cig users (n = 2,430, age > = 15) in 28 EU countries	Most common reasons in descending order for choosing the brand of e-cigs are Flavor, price and amount of nicotine.	Eurobarometer survey,1
Leigh, 2016 [44]	Six types of ENDS with five different flavors	Product type, battery output voltage, and flavors affect the toxicity of e-cig, and strawberry-flavored products are the most cytotoxic.	Lab test, 1
Litt, 2016 [45]	Young adult and adult cigarette smokers substituting e-cigs (n = 88, age 18–55)	The largest drop in cigarette smoking was associated with menthol e-cigs, and the smallest drop was associated with chocolate and cherry flavored e-cigs.	Experiment, 2
Marynak, 2017 [46]	E-cig products	In 2015, almost all e-cigs sold in most U.S. retail outlets (excluding vapor shops and online ones) contain nicotine.	Nielsen company, 1
Miech, 2017 [47]	Students, nationally representative (n = 44,892, grades 8, 10, and 12)	Two-thirds of students used vaporizers with just flavoring such as e-cigs, while 20% of 12th and 10th grade and 13% in 8th grade used products with nicotine.	Survey, 1
Morean, 2016 [48]	High and middle school students (n = 513, age mean 16)	The shares of adolescents using nicotine-free e-liquid, nicotine e-liquid, or not knowing their e-liquid nicotine concentration are similar.	Survey, 1 &2
Nonnemaker, 2016 [49]	Adult cigarette smokers (n = 765, age > 18)	For cigarette-only users, losing flavors significantly reduced the willingness to pay for an e-cigarette.	Online survey, 1&2
Oncken, 2015 [50]	Smokers (N = 27, age 18–55)	Using nonpreferred flavors by women leads to lower nicotine concentrations.	Experiment, 1
Patel, 2016 [51]	Current young adult and adult e-cig users (n = 2,448, age >18)	The likelihood of flavoring as a reason for e-cig use is greater among 18 to 24 years old than the elders.	Online survey, 2
Pepper, 2013 [52]	Male adolescents (n = 228, age 11–19)	No difference observed between willingness to try plain versus flavored e-cigs.	Online survey, 1
Pepper, 2016 [53]	Adolescents (n = 1,125, age 13–17)	E-cigs with menthol, candy or fruit flavoring are more interesting than tobacco or alcohol flavoring. Fruit-flavored e-cigs were perceived to be less harmful than tobacco flavored ones. 20% of adolescents thought e-cigs had no nicotine or were unsure.	Phone survey, 1&2
Pineiro, 2016 [54]	e-cig users (n = 1,815, age = >18)	Women are more likely to use disposable, non-tobacco flavored, lower nicotine strength, and first-generation types of e-cigs.	Online survey, 1
Polosa, 2015 [55]	Adult smokers (n = 71, age > = 18) in Italy	Smokers reduce nicotine strength of e-cig nicotine and switch from standard refillable to more advanced devices over time.	Experiment, 1
Rosbrook, 2016 [56]	Adult smokers (n = 32, age 18–45)	Menthol flavor can reduce perceived irritation and harshness of high nicotine concentration e-cigs.	Experiment, 1
Seidenberg, 2016 [57]	The top nine e-cig brand websites	Brands developed by cigarette manufacturers were not available in disposable models, advanced systems (e.g., tanks) or nicotine-free options.	Websites, 1
Shang, 2017 [58]	Ever and never e-cig users (n = 515, age 14–17)	Flavor has the biggest effect in choosing e-cigs in comparison to device type and warning. The probability of choosing e-cig among youth increases with fruit/sweets/beverage flavors.	Online survey, 2
Shiffman, 2015 [59]	Nonsmoking teens and adult smokers (n = 648, age 13–80)	Flavor does not affect nonsmoking teens’ interest for e-cig, but adults’ interest varies by flavor.	Online survey, 1
Simmons, 2016 [60]	E-cig users (n = 31, mean age 49)	Some users match e-cig flavors with their combustible cigarettes while some use totally different flavors from their cigarettes.	Focus groups, 1
Smith, 2016 [61]	Adult tobacco users (n = 1,443, age > 17)	The first use of a flavored tobacco product is related to current flavored tobacco use and polytobacco use. Young black non-Hispanic adults were more interested in using flavored tobacco products.	Phone survey, 1&2
Soule, 2016a [62]	Past 30-day e-cig users (n = 108, mean age 35)	Younger users enjoy a variety of flavors in e-cigs. Most popular: less than 8 mg/ml, 8–16 mg/ml, more than 16 mg/ml (strength); tank, drip, prefilled (type).	Online survey, 3
Soule, 2016b [63]	Past 30-day e-cig users (n = 46, mean age 38)	Flavored e-cigs increase satisfaction/enjoyment and feel/taste better than cigarettes.	Online survey, 3
Soussy, 2016 [64]	Aerosols of e-liquids under various vaping conditions	The addition of sweeteners to e-cig liquids exposes vapors to furans, a toxic class of compounds.	Lab test, 1
St. Helen, 2017 [65]	E-cig users (n = 14)	Flavors may influence the rate of nicotine absorption through an effect on Ph and can affect nicotine concentrations for women vapers.	Experiment, 1
Sussman, 2014 [66]	Online Yelp reviews for 103 e-cig shops	The most important vape shop attributes were the selection of flavors or hardware, fair prices, and unique flavors or hardware.	Yelp reviews,1
Tierney, 2016 [67]	Multiple flavors of two e-cig brands	Some flavored e-liquids contain high doses of chemicals which are unsafe when inhaled.	Lab test, 1
Villanti, 2013 [68]	Young adult tobacco users and non-users (n = 4,196, age 18–34)	Young black adults with high school degree are more likely to use flavored tobacco products.	LYACSj 2
Villanti, 2017 [69]	Adults and youth (n = 45,971, age > = 12)	Flavor is the primary reason for using any tobacco product, especially for youth and young adults.	PATH^k^, 2
Wagoner, 2016 [70]	Adolescence and young adult tobacco users and non-users (n = 77, age 13–25)	Flavor variety, user control of nicotine content, and smoke trick facilitation are positive attributes of e-cig.	Focus groups, 1
Wang, 2015 [71]	E-cig flavor content Reddit posts (n = 493,994)	Fruit, cream flavors are the most popular e-cig flavor categories, and most often used in flavor mixing.	Reddit posts, 1
Yingst, 2015 [72]	Cigarette smokers with at least 30 days of using e-cig (n = 4,421, mean age 40)	Battery capabilities and e-liquid flavor influenced device choice. Current advanced generation of e-cigs produces a more satisfying hit.	Online survey, 1
Yingst, 2017 [73]	E-cig users (n = 3,716)	Most common e-liquids flavors used by experienced e-cig users are tobacco, menthol/mint, and fruit.	Online survey, 1

^a^Method is indicated using numbers in which: 1 = Descriptive, 2 = Regression, 3 = Concept Mapping.

^b^E-cig is used for E-cigarette in this table.

^c^National Youth Tobacco Survey.

^d^Youth Tobacco Policy Survey.

^e^Texas Adolescent Tobacco and Marketing Surveillance System.

^f^ Marketing and Promotions Across Colleges in Texas Project.

^g^ Tobacco Products and Risk Perceptions Survey.

^h^Adolescent Health and Lifestyle Survey.

^i^ ENDS: electronic nicotine delivery systems.

^j^Legacy Young Adult Cohort Study, 2012.

^k^ Population Assessment of Tobacco and Health.

### Consumer preference for e-cigarette flavor

A survey of U.S. young adult and adult tobacco users found that flavored e-cigarettes are the fifth most frequently used flavored tobacco products out of nine in total, after shisha, cigarillos/little cigars, snus/smokeless, and pipes, and ahead of menthol cigarettes [61]. Similar results (except that pipes were the second most popular) were reported in another study of U.S. young adults and adults using a different data source [68]. Another study showed that among U.S. youth, just flavoring (no nicotine) was the most commonly vaped substances [47]. Flavored e-cigarettes were also found to be the first e-cigarettes for most youth, young adults, and adults vapers [34]. Furthermore, vapers ranked the selection of flavors and unique flavors as two of the most important factors in choosing between competing vape shops [66]. Based on social media data, a study found that the most frequently discussed flavors are fruit, cream, tobacco, and menthol [71]. Another study found that tobacco, menthol/mint, and fruit are the top three flavors preferred by consumers [73].

In the following subsections, we discuss consumer preference by three age cohorts, the impact of flavors on quitting smoking, and the health implications of flavors. The three age cohorts are adolescent, young adults, and adults, commonly defined by younger than 18, between 18 and 24, and older than 24; age groups are defined based on the National Health Interview Survey age groups definition [74]. Not all reviewed studies follow the above age cutoffs, so we used some discretion in classifying studies by age, and sometimes will use the mean age to determine the appropriate age cohort. To make our results more easily understandable, we tabulate results in Table 2, with +,–, and 0 representing the results of a study that found a positive preference, a negative preference, and no preference, respectively. We also use subscripts to denote results specific to smokers and non-smokers preference whenever possible.

**Table 2:** A summary of preference for e-cigarette flavors.

	Age cohorts			Help quit smoking?	Health	
List of Flavors	Adolescents	Young adults	Adults		Increase toxicity?	Increase Harm perception?
**Bitterness/harshness**			–[36]			
**Candy**	0 ^NS^[52], + ^NS^[53]		+[15]			
**Cherry**		+[22]				
**Coffee**		+^NS^[22]		+[22]		
**Coolness**			+[36]			
**Fruit**	+^NS^ [31, 53], 0 ^NS^ [52], +[58]		+^S^[24], +[15]			–[31]
**Menthol**	+^NS^[53]	+^NS^[22], +[42]	+^S^[24, 56], +[15]	+[22, 45, 49]		
**Strawberry**					+[44]	
**Sweet**	+^NS^[31], +[41, 58]	+[33, 35]	+[36]		+[64]	–[31]
**Tobacco**	– [20]	–[34]	+^S^[13, 22, 24]			+[31, 53]
**Flavor in general**	+[11, 14, 23, 34, 40, 69, 70], 0[52], 0^NS^[59]	+[11, 19, 21, 38, 51, 69, 70]	+[27, 61–63] +^S, NS^[24]	–[23]	+[35, 44, 67]	+[23]

Note: +,–, and 0 denote that a study finds a positive preference, a negative preference, and no preference, respectively. Generally, these studies do not distinguish between smokers and nonsmokers. Superscripts S and NS are smokers and non-smokers respectively.

### Adolescents’ preference for flavor

Thirteen papers described adolescents’ preference for flavor [14, 20, 23, 31, 34, 40, 41, 52, 53, 58, 59, 69, 70]. A recent study showed that most adolescents started first e-cigarette with flavored ones [69]. Another study found that adolescents positively regarded e-cigarette flavor variety [70]. A study using longitudinal surveys from middle and high school students found flavoring is the second most important factor determining whether students try e-cigarettes, after curiosity and another study also reported the same findings [14, 40]. As to flavor and smoking initiation, flavored e-cigarette use was found to be associated with a higher intention to initiate cigarette use [23]. A study based on a national sample of U.K. adolescents found fruit and sweet flavors were more likely to be tried by adolescents who have never smoked than by smokers trying to quit [31]. A more recent study confirmed this using a choice experiment in the United States [58]. Another U.K. study also found tobacco flavor was less favorable compared with other ones such as fruit [20]. A phone survey reported that adolescents (mostly non-smokers) were more likely to try e-cigarettes with candy, fruit, and menthol flavors than tobacco or alcohol flavors [53]. A study reported that sweet flavors were most popular among Connecticut adolescents [41]. On the other hand, another study, conducted by mostly the same authors used an online survey to demonstrate that flavors (i.e., candy or fruit) did not increase adolescents’ willingness to try e-cigarettes, [52] offering a different view of U.S. adolescence preference for flavor. Another study also reported a similar finding, that e-cigarette flavors do not appeal much to nonsmoking teenagers [59].

### Young adults’ preference for flavor

Eleven papers studied young adults’ preference for flavor [11, 19, 21, 22, 34, 35, 38, 42, 51, 69, 70]. A study found that similar to adolescents, young adults also positively regarded e-cigarette flavor variety [70]. French-speaking students also ranked flavors as the third most important reason for trying e-cigarettes, after curiosity and being offered by someone to try [38]. Furthermore, young adults seemed more likely to cite flavoring as a reason for use, especially compared with much older adults [51]. For the United States, a study showed that Texas adolescents and young adults were more likely to consider their first e-cigarettes to taste different from tobacco, compared with adults [34]. A study using lab experiments found that flavoring reinforced the desire to vape e-cigarettes containing nicotine [11]. A study using a focus group found that flavors contributed to positive perceptions of new e-cigarette products [19, 21]. In terms of specific flavors, a study found that sweet-flavored solutions produced greater appeal [35]. Using an online discrete choice experiment a study found that non-smokers were more interested in trying coffee, cherry, and menthol flavors while smokers were more interested in trying cherry flavor compared with other flavors [22]. One study found that high concentration of menthol led to better e-cigarette liking and wanting [42].

### Adult’s preference for flavor

A total of thirteen studies described adults’ preference for flavor [13, 15, 22, 24, 27, 36, 49, 54, 56, 60–63]. Two studies of adults using a concept mapping approach found that the variety of e-cigarette flavors was one reason they used e-cigarettes, and flavors may enhance the experience of e-cigarette use, respectively [62, 63]. A study of 33 countries mostly on ex- and current smokers showed that the most popular (or preferred) e-cigarette flavors in descending order were tobacco, fruit, and menthol [24]. For the United States, a study found that the descending order was fruit, menthol/mint, and candy/chocolate/other sweet flavors [15]. Another study on Malaysia showed that variety of flavors leads to better enjoyment [27].

A study found the first use of a flavored tobacco product was related to current flavored tobacco use and polytobacco use [61]. A study showed that older smokers [22] and another one showed current smokers [13] were more interested in trying tobacco-flavored e-cigarettes. A study compared gender differences in flavor preference and showed that men preferred tobacco flavors more than women did [54]. Another study indicated that adults preferred flavors that elicit sweetness or coolness while flavors that elicit bitterness or harshness (most likely coming from nicotine) were less preferred [36]. Flavors also generate a price premium for e-cigarettes by increasing consumers’ willingness to pay. In particular, a study of Florida smokers (92% adults and the rest young adults) concluded that willingness to pay for a flavor-less e-cigarette was significantly less than that for flavored product [49].

### Flavors and smoking cessation

Only four studies touched on the relationship between e-cigarette flavors and quitting smoking [17, 22, 23, 49]. One found that menthol and coffee flavors were perceived as having greater quit efficacy [22]. Another study also had a similar finding but only for menthol [49]. A study also found that using a combination of two or more flavors mixed together was more likely to quit smoking [17]. However, in another study, flavored e-cigarette use was found to be associated with a lower intention to quit smoking [23].

### The impact of flavor on health and harm perception

Seven studies addressed the impact of flavor on health and harm perception [23, 31, 35, 44, 53, 64, 67]. An analysis of 28 e-cigarette liquids purchased in Germany identified the presence of a wide range of flavors and additives, including some compounds that are potentially allergenic [35]. Similarly, a study of 30 e-cigarette products in the U.S. market found that 13 were more than 1% flavor chemicals by weight, some of which were of potential toxicological concern (e.g., cause respiratory irritation) [67]. Another study found that the use of sweeteners in e-cigarettes can expose users to furans, toxic compounds [64]. Furthermore, a study of five flavors across six types of e-cigarettes found that flavors significantly affected the in vitro toxicity profile and the strawberry-flavored product is the most toxic [44].

In terms of harm perception, one study found that flavored e-cigarette use reduced the prevalence of perception of the dangers of tobacco use among youth [23]. Another study found more nuanced results, demonstrating that tobacco flavor increased harm perception while fruit and sweet flavors decreased harm perception among U.K. adolescents [31]. Similarly, a study in the United States found that, for U.S. adolescents, fruit-flavored e-cigarettes were perceived to be less harmful than tobacco flavored ones [53].

### Consumer preference for nicotine strength

Companies report nicotine strength in three ways: milligrams, percentages, or descriptors (e.g., low, medium, high) [75]. Nicotine strength depends on e-cigarette type and varies widely, for example, from 0.27 to 2.91 mg/15 puffs [26]. Nineteen studies addressed consumer preference for nicotine and/or the interaction of nicotine with flavors [16, 22, 24, 28, 29, 33, 37, 42, 43, 46–48, 50, 53–56, 65, 70]. One study showed that almost all e-cigarettes sold in most U.S. retail outlets (excluding vapor shops and online ones) contained nicotine [46]. Another study examined 33 countries and found that only 1% of the adult smokers exclusively used non-nicotine e-cigarettes and that the most popular concentration of nicotine was 18 mg/ml [24]. A study of Finnish adolescents found that e-liquids with nicotine were more popular with ever smokers while e-liquids without nicotine were more popular with never smokers [37]. A study found that nicotine was the second most commonly used vaped substance for U.S. youth, after pure flavoring and ahead of marijuana [47]. Despite this, about 20% of adolescents thought e-cigarettes had no nicotine or were unsure [53]. In another study, researchers reported the shares of Connecticut adolescents using nicotine-free e-liquid, nicotine e-liquid, and not knowing the nicotine concentration in their e-liquid were largely similar (about one-third each) [48].

One study showed that user control of nicotine content was a positive attribute of e-cigarettes [70]. Men were found to use higher nicotine doses, compared with women [54]. Amount of nicotine was found to be a leading reason for many European vapers to choose their brands of e-cigarettes (after flavor and price) [43]. A study found that low nicotine content increased intentions to try e-cigarettes, reduced harm perception, and was perceived as more effective at aiding in smoking cessation. Medium nicotine content was found to have the opposite effect of low nicotine content. They also found that younger non-smokers preferred no nicotine or low nicotine e-cigarettes while smokers preferred medium and high nicotine e-cigarettes [22], echoing the findings of another study in this area [37]. Another study also found smokers and heavier e-cigarettes users tended to prefer nicotine [48]. In contrary to findings from a study mentioned above [22], another study [28] found that e-cigarettes with a high level of nicotine provided stronger attenuation of craving for tobacco, based on e-cigarette users from over seven countries. A later study by the same author found that experienced vapers who are trying to quit smoking decreased the nicotine concentration by using refillable e-cigarettes but increased the overall consumption in the e-liquids overtime to compensate [29]. A similar finding of decreased use of nicotine strength was reported by another study as well [55]. However, the opposite was reported in another study and interpreted as a strong motivation to quit smoking rather than using e-cigarettes recreationally [16].

Several studies addressed potential interactions of flavors and nicotine strength/concentration. A study of young adult vapers showed that nicotine increased user reports of throat hit but did not enhance appeal or interact with flavor effects on appeal [33]. On the other hand, a recent study [42] found evidence (weakly statistically significant, p = 0.06) of positive nicotine*menthol interaction, echoed by another study as well [56]. Also, there is evidence that flavor may influence nicotine concentrations in women vapers (using nonpreferred flavors led to lower concentrations) [50]. The mechanism could be that flavors may influence the rate of nicotine absorption through an effect on pH [65].

### Consumer preference for types

Twelve studies touched on consumer preference for e-cigarette types [12, 18, 24, 25, 30, 32, 39, 41, 54, 57, 58, 72]. In general, e-cigarettes can be divided into three generations: cigarette resembling first generation, pen resembling second generation that uses larger batteries and tanks, and no-cigarette resembling third generation that features even larger-capacity batteries, more advanced atomizers, and adjustable power delivery [25]. There is an evidence that second-generation devices seemed to be more satisfying to U.K. e-cigarette users [25]. Similarly, another study found that newer-generation devices were more satisfactory and effective in smoke cessation [30].

A study of adult ever smokers found that consumer preference for e-cigarette types was associated with smoking cessation. Specifically, open systems were more likely to be used by former smokers than current smokers and were more likely to be used daily than closed systems. Interestingly, most users used either closed systems or open systems, and rarely used both [18]. Women were found to prefer disposable e-cigarettes, and young adults were found to pay more attention to modifiability [39, 54]. Modifiability also was found to increase the probability of initiating e-cigarettes among adolescents [58]. A study found that about three-fourths of smokers used a tank system, which allows users to choose flavors and strength to mix their own liquid [24]. Experienced e-cigarette users even ranked the ability to customize as the most important characteristic [12]. Also, a study reported that experienced users preferred rechargeable e-cigarettes over disposable ones [32]. A similar finding was reported for Connecticut adolescents [41].

A study that examined top-selling e-cigarette websites found that most independent e-cigarette brands offered advanced systems (as opposed to first-generation e-cigarettes) that might appeal more to experienced e-cigarette users or smokers wanting to quit. In contrast, this study found that e-cigarette brands developed or acquired by cigarette manufacturers did not offer advanced systems [57]. Another study used an online survey provided similar finding––e-cigarette users likely initiated use with a cigalike product and transitioned to an advanced system with more features [72].

## Discussion

### Principal findings

Several results emerge from our literature review. First, several studies have shown that consumers preferred flavored e-cigarettes and such preference varied with age group and smoking status. Adolescents could consider flavor the most important factor in their decision to try e-cigarettes and were more likely to initiate vaping through flavored e-cigarettes (especially fruit and sweet ones for non-smokers). Young adults overall preferred sweet, menthol, and cherry flavors, while non-smokers, in particular, preferred coffee and menthol flavors. Adults preferred sweet flavors, too and disliked flavors that elicit bitterness or harshness. Adult smokers (especially men) liked tobacco flavor the most, followed by menthol and fruit flavors. In terms of smoking cessation, we found inconclusive evidence on the role of flavored e-cigarettes.

Second, we also found that several flavors were associated with decreased harm perception (e.g., sweet and fruit) while tobacco flavor was associated with increased harm perception. Our review identified several studies showing that some flavor chemicals and sweeteners used in e-cigarettes could be of toxicological concern.

Third, in terms of nicotine strength, the literature demonstrated that nicotine increased throat hit and user control of nicotine content is a positive attribute of e-cigarettes. Consumer ranked nicotine strength as an important factor choosing among various e-cigarettes, though such preference could vary by smoking status, e-cigarette use history, and gender. Specifically, non-smokers and inexperienced e-cigarettes users tended to prefer no nicotine or low nicotine e-cigarettes while smokers and experienced e-cigarettes users preferred medium and high nicotine e-cigarettes. Men were found to prefer higher nicotine doses. The evidence on whether user increased or decreased nicotine strength over time seemed rather inconclusive.

Fourth, an interesting result that emerges from our review is the potential interactions between e-cigarette attributes. We identified a handful studies on the interactions between flavors and nicotine strength, and found weak evidence of positive interactions between the two (i.e., nicotine*menthol). Future studies on the interactions of e-cigarette attributes are warranted.

Finally, we found that newer-generation devices were more satisfying to consumers. Consumer preference for e-cigarette types could depend on smoking status, user experience, gender, and age. Women and inexperienced e-cigarette users were found to prefer disposable e-cigarettes, and experienced e-cigarette users and young adults were found to pay more attention to modifiability. Open systems were more likely used by former smokers than current smokers and were more likely used daily, compared with closed systems. E-cigarette users likely initiated use with a cigalike product and transitioned to an advanced system with more features.

### Limitations

This study is the first comprehensive review of e-cigarette attributes. However, there are a few limitations to this review. First, although most reviewed studies on e-cigarettes indicated ethnicity, education, and income level in sample characteristics, a few of them analyzed consumer preferences across different races, incomes or education levels; for example, we only found four studies on preference for flavored e-cigarette by race [15, 51, 61, 68]. Therefore, we were not able to discuss our results across these demographics the same way that we did for age cohorts. Second, because of heterogeneity in demographic age ranges in the studies, we had to use some discretion (e.g., using mean age) matching individual studies to particular age cohorts. For example, in one study the age range is 18-30-years-old, and we considered it as a young adult cohort (18-24-years-old) [11]. Also, another study reported a mean age of 35 [63], and we placed it in the adult cohort (more than 25 years old). Finally, this study was restricted to peer-reviewed articles available in English, and most of them focused only on the United States (53 out of 66 studies), which limits the external validity of this research.

### Implications for research, policy, and practice

Our research generates many results that might be useful to policymakers and other researchers. First, the results summarized here provide insightful information regarding the potential impact of a restriction on certain e-cigarette attribute(s). For example, Canada bans the sales of e-cigarettes containing nicotine. If such a policy were adopted in the United States, it is reasonable to assume smokers will be affected the most by such a policy. Similarly, if the FDA bans the sale of all flavored e-cigarettes, we might expect to see a drop in e-cigarette initiation rate and prevalence rate. Second, our results point to a contradiction between facts and perception. For example, sweet flavor was perceived as less harmful though several studies indicated otherwise due to certain flavor chemicals. If consumers were informed of the potential harm of using flavored e-cigarettes, their purchasing decisions might change. Finally, our results also provide insight into research gap. For example, certain flavors such as strawberry and coolness receive little examination. There is also no study conducted on the potential interaction between flavor and types, and between nicotine strength and types.

## Conclusions

In this paper, we systematically reviewed peer-reviewed articles on three key e-cigarette attributes (flavors, nicotine strength, and type). We summarized main findings of 66 identified studies in two tables. Overall, our results reveal that consumers preferred flavored e-cigarettes that such preference varied with age groups and smoking status, that flavoring could be associated with toxicity, though many consumers believed otherwise. Consumer considered nicotine strength an important factor when purchasing e-cigarettes and found newer-generation devices are more satisfying to consumers; however, such preferences might depend on smoking status, e-cigarette use history, and gender.

## Supporting information

S1 Appendix Search strategy, included, and excluded articles full list. (DOCX)

S1 Table PRISMA checklist. (DOC)
