# Supplementary material for: A systematic review of consumer preference for e-cigarette attributes: Flavor, nicotine strength, and type
Source: PLoS One. 2018 Mar 15;13(3):e0194145. doi: 10.1371/journal.pone.0194145 (PMC5854347; doi:10.1371/journal.pone.0194145)
Supplement: S1 Appendix — (DOCX) [file pone.0194145.s001.docx]

**S1 Appendix. Search strategy, included, and excluded articles full list**

Samane Zare^1*^, Mehdi Nemati^1^, Yuqing Zheng^1^

^1^ Department of Agricultural Economics, University of Kentucky, Lexington, Kentucky, United States of America

^*^Corresponding author

E-mail: [samane.zare@uky.edu](mailto:samane.zare@uky.edu) (SZ)

# **Article Search Strategy**

**All information is based on final searches performed on January 8, 2018**

Our search strategy uses the Boolean search strategy to identify and collect studies that examined consumer preferences for e-cigarette attributes including flavor, strength, and type.

We use the following databases to identify potential articles: PubMed, MEDLINE, PsycINFO, CINAHL. We used Boolean Search Strategy and following search terms:

| **Level 1: Electronic Cigarettes** |
| --- |
|  |
| Electronic Cigarettes or E-cigarettes or Electronic Nicotine Delivery Systems or E-cig or E-cigarette |

Results of articles from the above process of the search strategy appear in the following chart.

Flavor (N=48)

Strength (N=22)

Type (N=14)

Records excluded based on full article review (N=570)*

Studies included in review (N=66)

12,297 articles excluded based on title, abstract, or duplication

Total full articles reviewed (N=636)

Total Records (N= 12,933)

PubMed (N=3,802)

MEDLINE (N=3,277)

Web of Science (N=3,421)

PsycINFO (N=1,021)

CINAHL Plus (N=1,412)

Fig 1. Studies screened and selected for inclusion in the review of consumer preferences for E-cigarette attributes.

Notes: * These articles are reported in the excluded section of the appendix.

Search process started on October 1^st^, 2016 and finished on January 8^th^, 2018. A list of each excluded articles based on a review of the full text appears in this appendix along with an explanation for exclusion.

# **Included Articles List (N=66)**

1. Audrain-McGovern, Strasser, and Wileyto (2016)
2. Baweja et al. (2016)
3. Berg (2016)
4. Bold et al. (2016)
5. Bonhomme et al. (2016)
6. Browne and Todd (2018)
7. Camenga et al. (2017)
8. Chen, Zhuang, and Zhu (2016)
9. Choi et al. (2012)
10. Clarke and Lusher (2017)
11. Cooper, Harrell, and Perry (2016b)
12. Czoli et al. (2016)
13. Dai and Hao (2016)
14. Dawkins et al (2015)
15. Dawkins et al (2013)
16. EL-Hellani et al. (2016)
17. Elkalmi et al. (2016)
18. Etter (2015)
19. Etter (2016a)
20. Etter (2016b)
21. Fierman et al. (2016)
22. Ford et al. (2016)
23. Giovenco, Lewis, and Delnevo (2014)
24. Goldenson et al. (2016)
25. Harrell, Weaver et al. (2017)
26. Hoffman et al. (2016)
27. Huang et al. (2017)
28. Hutzler et al. (2014)
29. Kim et al. (2016)
30. Kinnunen et al. (2016)
31. Kinouani, Pereira, and Tzourio (2017)
32. Kistler et al. (2017)
33. Kong et al. (2015)
34. Krishnan-Sarin, Green, et al. (2017)
35. Krishnan-Sarin et al. (2015)
36. Laverty, Vardavas, and Filippidis (2016)
37. Leigh et al. (2016)
38. Litt, Duffy, and Oncken (2016)
39. Marynak et al. (2017)
40. Miech et al. (2017)
41. Morean, Kong, Cavallo, et al. (2016)
42. Nonnemaker, Kim, Lee, et al. (2016)
43. Oncken et al. (2015)
44. Patel et al. (2016)
45. Pepper et al. (2013)
46. Pepper, Ribisl, and Brewer (2016)
47. Pineiro et al. (2016)
48. Polosa et al. (2015)
49. Rosbrook and Green (2016)
50. Seidenberg, Jo, and Ribisl (2016)
51. Shang et al. (2017)
52. Shiffman et al. (2015)
53. Simmons et al. (2016)
54. Smith et al. (2016)
55. Soule et al. (2016)
56. Soule, Rosas, and Nasim (2016)
57. Soussy et al. (2016)
58. St.Helen et al. (2017)
59. Sussman et al. (2014)
60. Tierney et al. (2015)
61. Villanti et al. (2013)
62. Villanti, Johnson, et al. (2017)
63. Wagoner et al. (2016)
64. Wang, Zhan, et al. (2015)
65. Yingst et al. (2015)
66. Yingst et al. (2017)

# **Excluded Articles List (N=570)**

1. Abo-Elkheir and Sobh (2016)
2. not relevant, measures consumers awareness and perception of e-cig
3. Agaku et al. (2017)
4. Investigated the relationship between receptivity to electronic cigarette (e-cigarette) advertisements at baseline and e-cigarette use at follow-up among adult baseline non-users of cigarettes and e-cigarettes.
5. Ahern and Mechling (2014)
6. Not relevant
7. Aherrera et al. (2017)
8. Not relevant.
9. Ajjandaleh et al. (2017)
10. Not related to e-cig characteristics, it is related to consumer characteristics
11. Akre and Suris (2017)
12. Not related to the perceptions of e-cig attributes.
13. Alawsi, Nour, and Prabhu (2015)
14. Investigated Advantages and disadvantages of using e-cigarette but not related to the e-cigarette attributes that we focus in this study.
15. Alcalá, Albert, and Ortega (2016)
16. Consumer demographics, not preference for e-cig attributes
17. Al-Delaimy, Myers, and Strong (2015)
18. Factors effecting e-cig consumption drop, not related to consumer preferences for e-cig attributes
19. Alexander et al. (2016)
20. They explored the terminology of adult e-cigarette users in describing e-cigarette products and
21. Alexander et al. (2015)
22. The research is related to how nicotine in e-cig is highly addictive and may predispose to inflammatory, infectious, and neoplastic diseases.
23. Allem, Forster, et al. (2015)
24. E-cig users’ characteristics and reasons to use. Not mentioned e-cig attributes that are the focus of this review study.
25. Allem, Unger, et al. (2015)
26. Studies safety perception of e-cig.
27. Allen et al. (2016)
28. Chemicals in e-cig flavors, not consumers preference for flavors
29. Amato, Boyle, and Levy (2016)
30. Not relevant to a preference for e-cig attributes.
31. Ambrose et al. (2015)
32. Letter.
33. Ambrose et al. (2014)
34. Not related to consumer preference for e-cig attributes, related to consumers’ perceptions on e-cig harmfulness.
35. Amrock, Lee, and Weitzman (2016)
36. Not related to consumer preference for e-cig attributes, related to consumers perceptions on e-cig.
37. Amrock et al. (2015)
38. Not related to consumer preference for e-cig attributes, related to consumers perceptions on e-cig.
39. Anand et al. (2015)
40. Consumers demographics, nothing related to consumer preference for e-cig attributes.
41. Andler et al. (2016)
42. Consumers distribution, nothing related to consumer preference for e-cig attributes.
43. Arane and Goldman (2016)
44. Summary- not relevant.
45. Arrazola et al. (2015)
46. Students tobacco use distribution among different tobacco products, not a preference for e-cig attributes.
47. Ashford et al. (2017)
48. Advertisement exposer and e-cig consumption, not related.
49. Ashford et al. (2016)
50. Consumers distribution and demographics b, nothing related to consumer preference for e-cig attributes.
51. Aszyk et al. (2017)
52. Lab test for flavoring additives determinants, not related to preference for e-cig flavor.
53. Awan (2016)
54. Consumers demographics and e-cig users’ distribution, nothing related to consumer preference for e-cig attributes.
55. Ayers and Allem (2017)
56. Tweeters analysis on why consumers try e-cig, no information about e-cig attributes preference.
57. Ayers et al. (2016)
58. Consumers search for e-cig related terms and issues, not related to consumer preference for e-cig attributes
59. Azagba, Baskerville, and Foley (2017)
60. Cigarette consumption among e-cig users
61. Babineau, Taylor, and Clancy (2015)
62. e-cig users’ characteristics, not e-cig attributes
63. Baeza-Loya et al. (2014)
64. Study on the safety perceptions of e-cigs during pregnancy which is not related to our study.
65. Baggett et al. (2016)
66. e-cig user’s characteristics and survey responders’ demographics, not e-cig attributes preference
67. Ballbe et al. (2014)
68. Indoor pollution in smokers’ home
69. Bandiera et al. (2016)
70. Study on the relationship between use of e-cigs and other tobacco products with nicotine and depression which is unrelated to our study.
71. Barnett et al. (2015)
72. Comparing students, demographics and smoking status of e-cig users
73. Barrington-Trimis, Samet, and McConnell (2014)
74. Flavor health hazards, not consumer preference for flavor
75. Barrington-Trimis, Urman, Berhane, et al. (2016)
76. E-cig users’ distribution.
77. Barrington-Trimis, Urman, Leventhal, et al. (2016)
78. E-cig uses distribution among students.
79. Basch et al. (2016)
80. Related to e-cig advertisement.
81. Bauhoff, Montero, and Scharf (2017)
82. Smokers and non-smokers e-cig knowledge and perceptions. Not relevant features of e-cig studied.
83. Bauld (2016)
84. Not relevant- meta on relationship between e-cig and cig quitting
85. Bauld et al. (2017)
86. This paper highlights the current rates of e-cigarette use among youth in the UK which is unrelated to our study.
87. Bauld et al. (2016)
88. Meta-note relevant
89. Baumann et al. (2015)
90. This paper study the difference between white and black races on e-cigs awareness and use history and advertisement which is unrelated to our study.
91. Beard et al. (2016)
92. Unrelated to our study.
93. Benowitz, Donny, and Hatsukami (2017)
94. Editorial.
95. Berg et al. (2016)
96. Instrumentality, Social Context, Displacement, and Experimentation, not preference for attributes, not only e-cig
97. Berg, Haardoerfer et al. (2015)
98. Reasons for switching to e-cig not preferences for this product attributes. Users’ characteristics and knowledge.
99. Berg, Stratton, et al. (2015)
100. Risk perceptions of different tobacco products including e-cig.
101. Biener and Lee Hargraves (2015)
102. consumers demographic and smoking status, not e-cig attributes
103. Biener et al. (2015)
104. Not related to e-cig attributes, demographics of users
105. Bitzer et al. (2017)
106. Technical. Studies flavors toxicological effects not preference for the flavors.
107. Bold et al. (2017)
108. This paper study the association between impulsivity and e-cigarette frequency which is unrelated to our study.
109. Booth, Albery, and Frings (2017)
110. Related to e-cig advertisement.
111. Borderud et al. (2014)
112. e-cig use among Patients with cancer
113. Bostean et al. (2016)
114. Presence of retailers and e-cig use
115. Bostean, Trinidad, and McCarthy (2015)
116. Consumers characteristics and e-cig use
117. Boulay et al. (2017)
118. Not relevant
119. Breheny et al. (2017)
120. Technical.
121. Breland et al. (2014)
122. Review study- not on consumer preference for e-cig attributes
123. Brikmanis, Petersen, and Doran (2017)
124. Consumers distribution/characteristics and e-cig use relationship
125. Brose et al. (2015)
126. Cessation attempt and e-cig use. Not relevant to e-cig attributes.
127. Brown and Cheng (2014)
128. e-cig types and differences among them, technical, not about users preference for different types
129. Brown, Beard, et al. (2014)
130. Not relevant, e-cig effectiveness
131. Brown, West, et al. (2014)
132. Consumers characteristics and e-cig use
133. Buchting et al. (2017)
134. Transgender use of tobacco products including e-cig. Not preference for e-cig attributes studied.
135. Bullen (2017)
136. Editorial
137. Bullen et al. (2013)
138. E-cig for smoking cessation, not relevant.
139. Bullen et al. (2014)
140. Not relevant.
141. Bullen et al. (2010)
142. e-cig effect on the desire to smoke
143. Bunnell et al. (2015)
144. Consumers characteristics and e-cig use relationship
145. Busch et al. (2016)
146. Perceptions, consumers demographics
147. Bush and Goniewicz (2015)
148. Home pollution in e-cig versus cig users
149. Camenga et al. (2015)
150. Perceptions of Electronic Cigarettes for Smoking Cessation
151. Cameron et al. (2014)
152. Not relevant.
153. Campbell-Heider and Snow (2016)
154. Review- summary- not relevant
155. Caponnetto et al. (2014)
156. Short article, overview, policy options
157. Caraballo et al. (2016)
158. Distribution of e-cig users by demographics
159. Cardenas et al. (2015)
160. Family smoking habits and e-cig use relationship.
161. Cardenas et al. (2016)
162. Distribution of e-cig users by demographics
163. Carr (2014)
164. Marketing strategies, e-cig users by demographics, review
165. Case et al. (2016)
166. Perceptions, not preferences
167. Case et al. (2017)
168. Demographics, technical, not relevant
169. Cavalcante et al. (2017)
170. Not relevant, non-English language full text
171. Chaffee, Couch, and Gansky (2017)
172. Users characteristics and e-cig use relationship, user status on smoking
173. Chapman, Daube, and Maziak (2016)
174. Perspective, review, not relevant
175. Chen, Zhu, and Conway (2015)
176. Online discussion forums, not preference for e-cig attributes
177. Chen (2013)
178. Not relevant.
179. Cheney, Gowin, and Wann (2016)
180. This paper studies belief and reasons for using e-cig. There is no information about preference for e-cig type, strength and different types of e-cig flavors.
181. Cho, Shin, and Moon (2011)
182. e-cig experience and information, demo and environmental factors effect e-cig consumption, not e-cig attributes in the study.
183. Choi and Bernat (2016)
184. Asthma and e-cig consumption, not relevant
185. Choi and Forster (2013)
186. Perceptions, awareness, not preference for e-cig attributes
187. Choi and Forster (2014)
188. Perceptions, not preferences, smoking status and e-cig perception
189. Choi, Grana, and Bernat (2017)
190. Factors effects using e-cig, not e-cig attributes among these factors
191. Chu et al. (2015)
192. Twitter messages, not related to e-cig attributes
193. Clapp et al. (2017)
194. Not relevant
195. Cobb, Hendricks, and Eissenberg (2015)
196. Not relevant, technical, nicotine delivery of e-cig and its problems
197. Cobb and Sonti (2016)
198. Not relevant
199. Cole-Lewis et al. (2015)
200. Twitter analysis of e-cig related tweets, not relevant
201. Coleman (2016)
202. Dissertation, not peer-reviewed
203. Coleman et al. (2015)
204. Users distribution, users’ characteristics association with e-cig use, not e-cig attributes in the article
205. Coleman et al. (2016)
206. Perceptions, not preferences
207. Cooke et al. (2015)
208. Clinical commentary review, not review of preference for e-cig attributes
209. Cooper, Case, and Loukas (2015)
210. e-cig young users’ characteristics
211. Cooper, Case, et al. (2016)
212. E-cigarette dual users’ characteristics and perceptions, not preference
213. Cooper, Creamer, et al. (2016)
214. Dual and single users’ perception, not preference
215. Cooper et al. (2017)
216. Perceptions of dual and single e-cig users, not preferences
217. Cooper, Harrell, and Perry (2016a)
218. The paper is about perceptions and motivations for e-cig use. The preferences for e-cig attributes is not studied.
219. Copeland, Peltier, and Waldo (2017)
220. Perceptions of risks and benefits among different type of users, college students, not preferences
221. Copp et al. (2015)
222. Nicotine reduction effects of e-cig, technical, not preference for nicotine levels of e-cig
223. Cornuz (2015)
224. Prevention, regulation, not preference
225. Corsi and Lippert (2016)
226. Not relevant, schools differences in using e-cig
227. Costigan, Lang, and Collard (2014)
228. Different flavors risk, not related to preference for flavors
229. Cox and Jakes (2017)
230. Viewpoint, not preferences
231. Cummins et al. (2016)
232. Believes and knowledge, not preference
233. Cuomo, Miner, and Mackey (2016)
234. Online sale distribution and types, not users preference
235. Czoli et al. (2017)
236. Meta-analysis of risk perceptions among users of different tobacco products including e-cig. Not relevant.
237. Czoli et al. (2015)
238. e-cig consumers’ characteristics and distribution
239. Czoli, Hammond, and White (2014)
240. Perception and knowledge of e-cig, distribution of e-cig use
241. Dai and Hao (2017a)
242. Users demographics, distribution, not preference
243. Dai and Hao (2017b)
244. Tweeter analysis, not preference
245. Dautzenberg et al. (2015)
246. Factors associated with trying e-cig, e-cig attributes are not in the study
247. Davis et al. (2015)
248. Technical, nicotine levels of e-cig, not preference
249. Dawkins et al. (2012)
250. The paper studies the e-cig effects on the desire to smoke, nicotine withdrawal symptoms, attention and working memory.
251. Dawkins, Munafo, et al. (2016)
252. The effect of e-cig visual appearance on reducing craving and withdrawal symptoms.
253. De Lacy et al. (2017)

a. none of the e-cig attributes that are the focus of our study is studied as a consumers’ preferences.

1. Delnevo, Giovenco, et al. (2016)
2. E-cig use distribution among different groups.
3. Delnevo and Villanti et al. (2016)
4. Influence of different tobacco products including e-cig on the changes in the past year smoking.
5. Demissie et al. (2017)
6. Risky behaviors, e-cig users’ distribution/characteristics
7. Diamond (2016)
8. Different smoking cessation tools preference including e-cig, not preference for e-cig attributes
9. Dinakar and O'Connor (2016)
10. Not relevant
11. Dobbs, Hammig, and Henry (2017)
12. Perception, users’ characteristics
13. Dockrell et al. (2013)
14. Awareness, users distribution
15. Doran and Brikmanis (2016)
16. Users expectations, not about e-cig attributes
17. Doran et al. (2017)
18. e-cig and cigarette use relationship
19. Douptcheva et al. (2013)
20. Not relevant to e-cig attributes
21. Duderstadt (2015)
22. Policy brief, not peer-reviewed article
23. Duffy and Jenssen (2014)
24. Short review, not relevant
25. Dunlop et al. (2016)
26. users’ characteristics, distribution, age and e-cig use
27. Durmowicz (2014)
28. Not relevant
29. Durmowicz, Rudy, and Chen (2016)
30. Not relevant, e-cig adverse experiences.
31. Dutra and Glantz (2014a)
32. e-cig users’ characteristics, e-cig, and cig use
33. Dutra and Glantz (2014b)
34. Editorial, not related
35. Dutra and Glantz (2017)
36. Not related, distribution, smoking status.
37. Eastwood et al. (2015)
38. The study does not investigate any e-cig attributes.
39. Eastwood et al. (2017)
40. E-cig use trend. Not relevant.
41. Eggleston et al. (2016)
42. The study does not investigate any e-cig attributes.
43. Eissenberg and Shihadeh (2015)
44. Letter
45. Etter (2014)
46. Unrelated.
47. Etter (2017)
48. Unrelated.
49. Etter and Eissenberg (2015)
50. Compares dependence levels in e-cig users versus nicotine gums and tobacco cigarettes users.
51. Etter and Bullen (2011)
52. E-cig users’ characteristics, perceptions, and efficacy of e-cig to quit smoking.
53. Etter and Bullen (2014)
54. This paper studies the changes in the behavior of users of electronic cigarettes over time.
55. Fallin et al. (2016)
56. Perceptions and beliefs about e-cig use among pregnant and postpartum women.
57. Farsalinos et al. (2015)
58. Technical on nicotine levels of e-cig, not related.
59. Farsalinos and Polosa (2014)
60. Risks associated with e-cig, review, not relevant.
61. Farsalinos et al. (2016)
62. e-cig popularity and users’ distribution among smokers, never smokers, etc. not related to e-cig attributes.
63. Farsalinos et al. (2017a)
64. Letter to the editor.
65. Farsalinos et al. (2017b)
66. Nicotine delivery in e-cig, technical, not preference
67. Farsalinos, Romagna, and Voudris (2015)
68. Factors effect dual use of tobacco and e-cig. Preference for the e-cig attributes that are the focus of our study is not studied.
69. Fearon et al. (2017)
70. Technical. Nicotine delivery comparing cig and e-cig.
71. Ferkol (2017)
72. Keynote speaker
73. Filippidis et al. (2017)
74. Smokers and nonsmokers use of e-cig
75. Fillon (2016)
76. News
77. Flora et al. (2016)
78. Not related
79. Fotiou et al. (2015)
80. users’ characteristics, short communication
81. Foulds (2015)
82. Editorial
83. Foulds et al. (2015)
84. Nicotine dependence and e-cig type, not consumer preference for e-cig attributes
85. Franck et al. (2014)
86. Review, e-cig use and its effectiveness in reducing cigarette use, not related
87. Franks et al. (2017)
88. Knowledge and experience among students, not related to preference
89. Fulmer et al. (2015)
90. Advertisement and cig use. Not relevant.
91. Furlow (2017)
92. News.
93. Gallart-Mateu et al. (2016)
94. Unrelated.
95. Gallus et al. (2014)
96. E-cig awareness among different groups.
97. Ganz et al. (2015)
98. Related to e-cig advertisement.
99. General (2016)
    1. Not relevant.
100. Gilreath et al. (2016)
     1. This study examined patterns of adolescent use of cigarettes, e-cigarettes, cigars/cigarillo, hookah/waterpipe, and smokeless/dip/chewing tobacco in a population of southern California adolescents.
101. Giovenco et al. (2016)
102. Unrelated.
103. Giovenco and Delnevo (2018)
104. E-cig use and smoking [cessation.](https://www.google.com/search?rlz=1C1PRFC_enUS762US762&q=smoking+cessation.&spell=1&sa=X&ved=0ahUKEwiF58KqxdrYAhWC54MKHXeCCssQkeECCCQoAA)
105. Giovenco et al. (2015)
106. Not relevant.
107. Glasser et al. (2015)
108. Unrelated.
109. Glasser et al. (2017)
110. Not relevant.
111. Goh et al. (2017)
112. E-cig awareness and users characteristics.
113. Goney (2017)
114. Review study, not relevant.
115. Goldenson et al. (2017)
116. The effect of e-cig nicotine concentration on smoking behavior.
117. Goniewicz et al. (2013)
118. Technical, nicotine levels in different e-cigs.
119. Goniewicz et al. (2014)
120. It is about the growth of e-cigarette use in Poland.
121. Goniewicz et al. (2015)
122. Technical, comparing nicotine levels in e-cigs from U.S., Poland, and South Korea.
123. Goniewicz et al. (2016)
124. Users’ characteristics and distribution.
125. Goniewicz, Lingas, and Hajek (2013)
126. Users’ beliefs about e-cig safety and benefits. Not relevant.
127. Goniewicz and Zielinska-Danch (2012)
128. Not about any e-cigarette attributes.
129. Gonzalez-Roz, Secades-Villa, and Weidberg (2017)
130. Letter
131. Gorukanti et al. (2017)
132. Unrelated to the e-cig attributes we considered in our study.
133. Gostin and Glasner (2014)
134. Opinion
135. Gowin, Cheney, and Wann (2017)
136. Beliefs and knowledge about e-cig, none of the e-cig attributes that are the focus of our study included in this study.
137. Grace, Kivell, and Laugesen (2015a)
138. E-cig price elasticity of demand, not relevant.
139. Grace, Kivell, and Laugesen (2015b)
140. Nicotine takes after using e-cig in men and women.
141. Grana, Benowitz, and Glantz (2014)
142. Review study
143. Grana (2013)
144. Editorial
145. Gravely et al. (2014)
146. Unrelated.
147. Greenhill et al. (2016)
148. Review study
149. Gubner et al. (2016)
150. Unrelated to the e-cig attributes we considered in our study.
151. Guillet et al. (2015)
152. Unrelated to the e-cig attributes we considered in our study.
153. Guillory et al. (2016)
154. Unrelated
155. Haber and Ortiz (2014)
156. Research letter
157. Hajek et al. (2014)
158. Review, not relevant
159. Hall et al. (2016)
160. Reasons for using e-cig, not about preference for e-cig attributes
161. Hall and Gartner (2014)
162. Not relevant, policy options
163. Hammal and Finegan (2016)
164. Children attitude on e-cig, not preference for attributes
165. Hammett et al. (2017)
166. Users’ characteristics, not e-cig attributes
167. Hammig, Daniel-Dobbs, and Blunt-Vinti (2017)
168. User characteristics association with e-cig initiation, not e-cig attributes
169. Hammond et al. (2017)
170. e-cig users’ characteristics, smoker, and non-smokers
171. Hanewinkel and Isensee (2015)
172. e-cig users’ characteristics and distribution
173. Harrell, Marquinez, et al. (2015)
174. Consumers’ beliefs and risk perceptions on e-cig and cig.
175. Harrell, Simmons, et al. (2015)
176. Not relevant, user’s expectations
177. Harrington et al. (2014)
178. Users distribution by demographics and smoking status
179. Hartwell et al. (2016)
180. Review, not relevant, awareness
181. Hassan et al. (2016)
182. Not relevant, technical, burn injuries from e-cigs
183. Havel et al. (2017)
184. Aerosol variation in e-cigs with different attributes, not preference for the attributes
185. Hamilton et al. (2015)
186. E-cig users’ characteristics and distribution.
187. Hajek et al. (2017)
188. Technical.
189. Helen et al. (2017)
190. Technical.
191. Henningfield and Zaatari (2010)
192. Policy options, not relevant
193. Hershberger et al. (2017)
194. Believes in e-cig and cigarette
195. Hess, Antin, et al. (2017)
196. Not relevant, technical, chemical levels
197. Hess, Olmedo, et al. (2017)
198. Perceptions, not relevant
199. Higgins et al. (2015)
200. Review, gender differences, tobacco in general
201. Hildick-Smith et al. (2015)
202. Not relevant, e-cig general increases, not attributes
203. Hilton et al. (2016)
204. Teenagers views, they mention e-cig has a great flavor but did not mention which flavors are preferred to others.
205. Hinds et al. (2016)
206. How we can design better interview questionnaires’, which questions should be added to the surveys, not relevant
207. Hines, Fiala, and Hedberg (2017)
208. Weekly report, not peer-reviewed
209. Hirano et al. (2017)
210. Smoking cessation using e-cig
211. Hiratsuka et al. (2015)
212. Not relevant, views on e-cig in preventing smoking
213. Hiscock et al. (2015)
214. Knowledge and beliefs about e-cig. Not relevant.
215. Hitchman et al. (2015)
216. Distribution of e-cig users, users’ characteristics.
217. Hoong et al. (2017)
218. Users distribution by demographics and smoking status
219. Hooper and Kolar (2016)
220. Users demographics
221. Hooper and Kolar (2017)
222. Knowledge and perceptions of demographics
223. Huang, Tauras, and Chaloupka (2014)
224. Price elasticities, not preference for attributes we have in the article including type, flavor, and strength
225. Huang et al. (2016)
226. Users distribution and characteristics
227. Hubbs et al. (2015)
228. Commentary, not relevant.
229. Huerta et al. (2017)
230. Awareness and perception
231. Huh and Leventhal (2016)
232. Not relevant, cig and e-cig use covariance.
233. Hummel et al. (2015)
234. Awareness
235. James (2017)
236. Dissertation, not peer-reviewed
237. Jawad et al (2018)
238. Related to price elasticity of demand and not relevant to the preference for attributes.
239. Jeon et al. (2016)
240. Reasons for initiation of e-cig use
241. Jiang, Chen, et al. (2016)
242. Awareness, users’ distribution by demographics
243. Jiang, Wang, et al. (2016)
244. Users distribution and demographics
245. Jones et al. (2016)
246. vaping cannabis, not relevant
247. Jones et al. (2017)
248. Users distribution and demographics
249. Jorenby et al. (2017)
250. Nicotin levels on cig and e-cig users.
251. Kadimpati, Nolan, and Warner (2015)
252. Attitudes other than e-cig attributes.
253. Klager et al. (2017)
254. Technical.
255. Kaleta, Wojtysiak, and Polanska (2016)
256. It is about the use of e-cig among students and is unrelated to any of the e-cig attributes we considered in our study
257. Kalkhoran and Glantz (2016)
258. A systematic review of studies on the association between e-cig use and smoking quitting.
259. Kalkhoran et al. (2015)
260. Unrelated
261. Kalkhoran et al. (2016)
262. Unrelated
263. Kalousova (2015)
264. Comment.
265. Kamat and Van Dyke (2017)
266. Unrelated and not peer-reviewed journal.
267. Kanchustambham et al. (2017)
268. Unrelated to consumers and unrelated topic.
269. Kaplan (2015)
270. Not peer reviewed journal.
271. Kaufmann and Currie (2017)
272. Unrelated to the e-cig attributes we considered in our study.
273. Kenne et al. (2016)
274. E-cig users’ distribution and characteristics.
275. Khlystov and Samburova (2016)
276. Technical. E-cig flavors health risks and chemicals.
277. Khoury et al. (2016)
278. Unrelated to the e-cig attributes we considered in our study.
279. Kilibarda, Mravcik, and Martens (2016)
280. E-cig users’ distribution and characteristics.
281. Kinnunen et al. (2015)
282. E-cig users’ characteristics including socioeconomic conditions and type of the users. Preference for the e-cig type, flavor, or strength did not study.
283. Kim, Arnold, and Makarenko (2014)
284. E-cig advertising expenditures in the U.S. which are unrelated to our study.
285. Kim, Hopper, et al. (2015)
286. Unrelated topic.
287. Kim, Lee, et al. (2015)
288. Unrelated
289. Kim et al. (2017)
290. E-cig use behavior. Not relevant to e-cig attributes that are the focus of this study.
291. Kim and Baum (2015)
292. Not relevant. E-cig toxicity and need for regulations.
293. Kim, Kabir, and Jahan (2016)
294. E-cig health impacts.
295. King et al. (2016b)
296. Related to exposer to e-cig video and e-cig use.
297. King, Smith, et al. (2015)
298. Exposer to e-cig and desire to use.
299. King et al. (2013)
300. It is about awareness and ever-use of e-cig which is unrelated to our study.
301. King, Patel, et al. (2015)
302. E-cig effect of smoking a regular cig.
303. Kinnunen (2016)
304. Commentary
305. Kinnunen et al. (2017)
306. The frequency of use.
307. Kong et al. (2016)
308. Unrelated
309. Kong et al. (2017)
310. Unrelated to the e-cig attributes we considered in our study.
311. Kornfield et al. (2015)
312. It is an industry watch related to rapidly increasing promotional expenditures for e-cigs which is unrelated to our study.
313. Korry (2017)
314. News.
315. Kosmider et al. (2014)
316. Technical, lab-based research. Not relevant.
317. Kosmider et al. (2016)
318. Letter
319. Kotecha, Jawad, and Iliffe (2016)
320. Waterpipes and e-shisha, not e-cig. Not relevant.
321. Kralikova et al. (2013)
322. Potential competition between e-cig and cig and consumers experiences.
323. Krishnan-Sarin, Morean, et al. (2017)
324. Studied reasons for dripping.
325. Kristjansson et al. (2017)
326. Not relevat.
327. Kruse, Kalkhoran, and Rigotti (2017)
328. The relationship between comorbidities and e-cig use.
329. Kusiak et al. (2017)
330. Use of e-cig among Polish dental students and reasons for use are unrelated to e-cig attributes which are the focus of our study.
331. Lam and West (2015)
332. Not relevant.
333. Lanza, Russell, and Braymiller (2017)
334. The study shows that E-cig and traditional cigarette use are strongly associated throughout adolescence which is unrelated to our study.
335. Larson and Pearlman (2016)
336. The Study is about the characteristics of adolescents who do not smoke conventional cigarettes.
337. Lechner et al. (2015)
338. Unrelated.
339. Lee et al. (2015)
340. Unrelated.
341. Lee et al. (2017)
342. Reasons for adopting e-cig. Not a preference for e-cig attributes.
343. Lee et al. (2018)
344. E-cig perceptions and warning labels.
345. Lee, Kim, and Cho (2016)
346. E-cig users’ characteristics and history of smoking and drinking.
347. Lee, Grana, and Glantz (2014)
348. They studied the prevalence of e-cigarette use among Korean adolescents and the relationship between e-cigarette use and current smoking, cigarettes, attempts to quit conventional cigarettes, and cigarettes cessation.
349. Lee et al. (2014)
350. The study is about the multiple tobaccos uses among adults in the U.S.
351. Lehmann, Kuhn, and Reimer (2017a, 2017b)
352. Not in English.
353. Levy et al. (2017)
354. The paper provides a framework to evaluate the public health impact of e-cig which is unrelated to our study.
355. Levy, Yuan, and Li (2017)
356. The paper discusses the characteristics of e-cig users and does not cover their preferences for e-cigs attributes.
357. Li et al. (2015)
358. E-cig users charactristics.
359. Li, Newcombe, and Walton (2014)
360. It is related to use of e-cigs and attitude of consumers toward e-cig advertising, so it is unrelated to our study.
361. Li, Newcombe, and Walton (2015)
362. Letter
363. Li, Newcombe, and Walton (2016)
364. The paper does not discuss consumer preferences for e-cigs attributes.
365. Liang et al. (2016)
366. Conference paper
367. Lindblom (2015)
368. Unrelated
369. Liozidou et al. (2016)
370. The paper investigates prevalence and predictors of cigarette and e-cigarette smoking among adolescents. E-cigs attributes are not among the predictors.
371. Lippert (2015)
372. Unrelated
373. Lisko et al. (2017)
374. Caffeine concentration in different e-cig flavors. Not a preference for the e-cig flavors.
375. Littlefield et al. (2015)
376. This paper examines e-cigarette use and the relation of such use with gender, race/ethnicity, traditional tobacco use, and heavy drinking.
377. Loomis et al. (2016)
378. The paper assesses state-specific annual sales and average prices for e-cigs in the U.S.
379. Lotrean (2015)
380. E-cigs use among Romanian University students, none of the attributes that we considered in our paper is discussed in the Lotrean’s paper.
381. Loukas et al. (2018)
382. None of the attributes that we considered in our paper is discussed in this paper.
383. Lozano et al. (2017)
384. This study evaluated whether e-cigarette trial among Mexican adolescents increased the likelihood of trial and use of conventional cigarettes or marijuana use at follow-up which is unrelated to our paper.
385. Lund (2016)
386. Commentary.
387. Majeed et al. (2017)
388. This article study whether perceived relative harm of e-cigarettes and perceived addictiveness have changed during 2012–2015 among U.S. adults. So, it is unrelated to our study.
389. Malas et al. (2016)
390. Irrelevant review study.
391. Maloney and Cappella (2016)
392. The study is about the effect of visual e-cigs advertising on the urge to smoke and smoking behavior which is unrelated to our study.
393. Mantey et al. (2016)
394. Unrelated. This article studies the relationship between exposure to e-cigarette marketing and susceptibility and use of e-cigarettes in youth.
395. Margolis et al. (2016)
396. None of the attributes that we considered in our paper is discussed in this paper.
397. Mark et al. (2015)
398. None of the attributes that we considered in our paper is discussed in this paper.
399. Martinez (2018)
400. Dissertation.
401. Martinez-Sanchez, Ballbe, Fu, Carlos Martin-Sanchez, et al. (2014)
402. The study is about the use of e-cigs among adults in Spain, and none of the attributes that we considered in our paper is discussed in this paper.
403. Martinez-Sanchez, Fu, Carlos Martin-Sanchez, et al. (2015)
404. The study is about the perception of e-cigs in the general population, and none of the attributes that we considered in our paper is discussed in this paper.
405. Mayor (2015)
406. News.
407. Mays et al. (2016)
408. None of the attributes that we considered in our paper is discussed in this paper.
409. Mazza, McGrath-Morrow, and Collaco (2017)
410. Unrelated.
411. McCabe et al. (2017)
412. Unrelated. The study is about the e-cig use and its associations with some risky behaviors.
413. McCarthy (2014a)
414. News.
415. McCarthy (2014b)
416. News.
417. McCarthy (2015)
418. News.
419. McCarthy (2016)
420. News.
421. McCubbin et al. (2017)
422. It is about the use and perception of e-cigs during pregnancy which is unrelated to our study.
423. McDonald (2013)
424. Book review.
425. McDonald and Ling (2015)
426. Beliefs and opinions of e-cig users, not preferences for e-cig attributes.
427. McGraw (2015)
428. Not relevant.
429. McKeganey and Dickson (2017)
430. Unrelated
431. McMillen et al. (2015)
432. E-cig users characteristics and distribution, the frequency of use.
433. McPherson et al. (2016)
434. Unrelated.
435. Meernik et al. (2017)
436. Unrelated.
437. Mello et al. (2016)
438. The perceived harm to e-cigs and support for regulations.
439. Meltzer et al. (2017)
440. Unrelated.
441. Meyers, Delucchi, and Halpern-Felsher (2017)
442. Unrelated. The study determines from whom and where adolescents obtained tobacco, including cigarettes, e-cigarettes, and hookah.
443. Miao et al. (2016)
444. Technical. The comparison of sweeteners in alternative tobacco products.
445. Miller (2014)
446. News.
447. Moheimani et al. (2017)
448. Unrelated. The study determines the role of nicotine versus non-nicotine constituents in e-cigarette emissions in causing these pathologies in otherwise healthy humans.
449. Moore et al. (2016)
450. Unrelated. prevalence of e-cigarette uses in non-smoking children or associations with intentions to smoke.
451. Moore, McKee, and Daube (2016)
452. Viewpoint.
453. Morean et al. (2015)
454. Unrelated. The relationship between e-cig use and cannabis use.
455. Morean, Kong, Camenga, et al. (2016)
     1. Unrelated. The study is about adolescents’ use of e-cigarettes and other substances.
456. Morean et al. (2017)
     1. Unrelated. The study is about Predictors of Adult E-Cigarette Users Vaporizing Cannabis Using E-Cigarettes and Vape-Pens.
457. Morphett et al. (2016)
     1. Unrelated.
458. Mowls (2018)
     1. Dissertation.
459. Murthy (2017)
     1. Viewpoint.
460. Nădăşan et al. (2016)
     1. Unrelated. Use of electronic cigarettes and alternative tobacco products.
461. Nagelhout et al. (2016)
     1. Unrelated. The study examines whether noticing e-cigarette advertisements is associated with current use of e-cigarettes, disapproval of smoking, quit smoking attempts, and quit smoking success.
462. Naughton (2016)
     1. Unrelated survey.
463. Nayak, Kemp, and Redmon (2016)
     1. Unrelated. The study examines vape shop operators’ perceptions of benefits and risk of ENDS use, not consumers attitudes toward e-cig attributes.
464. Nayak et al. (2016)
     1. Electronic nicotine delivery system dual-use and intention to quit smoking.
465. Nichols et al. (2016)
     1. Unrelated. The study examines the relationship between e-cigarette cues and craving.
466. Nicksic et al. (2017)
     1. The study investigates the impact of tobacco marketing on e-cig susceptibility and perceptions.
467. Noland et al. (2016)
     1. Unrelated. The study is about Social Influences on Use of Cigarettes, E-Cigarettes, and Hookah by College Students.
468. Nowariak et al. (2018)
     1. Unrelated. The study examines the relationship between e-cig use frequency and abstinence among a sample of treatment-seeking tobacco users.
469. O'Connor et al. (2017)
     1. Effect of advertisement of e-cig demand.
470. Ooms et al. (2016)
     1. E-cig users’ characteristics.
471. Orr (2014)
     1. Not relevant review study.
472. Owotomo, Maslowsky, and Loukas (2017)
     1. Unrelated. The study examines how e-cig users compared with nonusers, conventional cigarette smokers, and dual users on perceptions of harm and the addictiveness of conventional cigarette smoking and on other known predictors of cigarette smoking. such as peer smoking, the influence of antismoking ads, and risk-taking propensity. None of the attributes that we considered in our paper is discussed in this paper.
473. Owusu et al. (2017)
     1. Unrelated. The study estimates the prevalence of e-cigarette use and examines the association of e-cigarette use with two tobacco products among school-going adolescents. None of the attributes that we considered in our paper is discussed in this paper.
474. Paek et al. (2014)
     1. Unrelated. The study examines message, source, and health information characteristics of e-cig videos on YouTube.
475. Palipudi et al. (2016)
     1. Unrelated. The study is about the awareness and current use of e-cig in middle and low-income countries
476. Park, Seo, and Lin (2016)
     1. Unrelated. The study examines the association of e-cig use with the intention to initiate or quit smoking.
477. Park, Lee, and Min (2017)
     1. Unrelated. The study examines the characteristics adults who have switched to e-cigs from traditional cigarettes.
478. Park, Duncan, Shahawy, et al. (2017)
     1. None of the attributes that we considered in our paper is discussed in this paper.
479. Park, Lee, et al. (2017)
     1. Unrelated. This study examines the association between individuals' levels of psychological distress and e-cig use.
480. Pasquereau et al. (2017)
     1. Unrelated. This paper investigates whether regular use of e-cigs for those who are smoking is associated with smoking cessation.
481. Patrick et al. (2016)
     1. The difference of students’ reasons for vaping based on their characteristics. Not preferences for different attributes of e-cig which are the focus of our study.
482. Pearson et al. (2017)
     1. How e-cig users refer to e-cig and users’ characteristics.
483. Pearson et al. (2012)
     1. Unrelated. The study estimates e-cig awareness, use, and harm perceptions among US adults.
484. Pearson et al. (2015)
     1. Unrelated. The study reports the associations between e-cigarette use and smoking cessation
485. Pénzes et al. (2016)
     1. Not preferences for different attributes of e-cig which are the focus of our study.
486. Pepper and Brewer (2014)
     1. Review study
487. Pepper, Emery, Ribisl, and Brewer (2014)
     1. Unrelated. The paper investigates how that U.S. adult hears about e-cigs.
488. Pepper et al. (2015)
     1. The paper examines the relationship between cigarettes and non-cigarette tobacco products use and smokers’ perceived likelihood of health problems.
489. Pepper, Emery, Ribisl, Southwell, et al. (2014)
     1. Unrelated. It is about the effects of advertisements on smokers’ interest in trying e-cigs.
490. Pepper, Ribisl, et al. (2014)
     1. The main reasons for starting e-cig are not the attributes of e-cig which are the focus of our study.
491. Persoskie et al. (2017)
     1. Unrelated. This study investigates the difference between youth beliefs about the harm of e-cigs and cigarettes.
492. Pesko, Huang, et al. (2017)
     1. Association between e-cig price and e-cig use.
493. Pesko et al. (2016)
     1. Unrelated. This paper estimates the effect of potential regulations of ENDS among adult smokers.
494. Pesko and Robarts (2017)
     1. Unrelated. This study evaluates the influence of sociodemographic and tobacco control policy environments on adolescent tobacco use in urban versus rural areas.
495. Peters et al. (2015)
     1. This paper is about awareness, perceptions, use, and reasons for use among adults seeking substance use treatment. None of the attributes that we considered in our paper is discussed in this paper.
496. Peters et al. (2013)
     1. E-cig attributes that are the focus of our study are not included in the study.
497. Pokhrel et al. (2016)
     1. Unrelated. E-cig advertising effect on non-smokers’ attitude.
498. Pokhrel, Fagan, et al. (2015)
     1. Unrelated. This study examines the relationship between exposure and receptivity to e-cigarette marketing and e-cig use.
499. Pokhrel, Herzog, et al. (2015)
     1. Young adults attitudes and experience.
500. Pokhrel et al. (2014)
501. Short Communication.
502. Popova and Ling (2014)
503. Warning labels on e-cig and smokers response.
504. Porter et al. (2015)
505. E-cig and cig use among students.
506. Postolache et al. (2015)
507. Smoking cessation and e-cig.
508. Potera (2015)
509. News.
510. Pratt et al. (2016)
511. The appeal of e-cig use among people with the serious mental illness.
512. Printz (2015)
513. Not relevant.
514. Printz (2017)
515. News.
516. Prochaska and Grana (2014)
517. E-cig use among people with the serious mental illness.
518. Pu and Zhang (2017)
519. Exposer to advertising and e-cig use.
520. Pulvers et al. (2015)
521. E-cig use and quitting behavior.
522. Rahman et al. (2015)
523. E-cig and smoking cessation.
524. Meta-analysis.
525. Rahman et al. (2014)
526. Use, health effects and smoking cessation of e-cig.
527. Ramo, Young-Wolff, and Prochaska (2015)
528. E-cig use trend using three studies.
529. Rankin (2017)
530. News.
531. Rass et al. (2015)
532. Perceptions and relative harm of e-cig and cig among users.
533. Rayens et al. (2017)
534. Not relevant.
535. Regan et al. (2013)
536. Awareness.
537. Reid et al. (2015)
538. E-cig users’ distribution.
539. Reinhold et al. (2017)
540. Advertisement and e-cig use by demographics.
541. Rennie, Bazillier-Bruneau, and Rouëssé (2016)
542. Frequecncy and demographics.
543. Richardson, Ganz, et al. (2014)
544. Related to e-cig advertisement.
545. Richardson, Ganz, and Vallone (2015)
546. Online advertising.
547. Richardson, Pearson, et al. (2014)
548. Perceptions and reasons for using cig in general. Not relevant.
549. Richter (2015)
550. Correspondence.
551. Riggs and Pentz (2016)
552. Not relevant.
553. Rigotti (2015)
554. Editorial.
555. Rigotti et al. (2015)
556. E-cig users characteristics and distribution by demographics.
557. Rigotti and Wu (2015)
558. Not relevant.
559. Robertson et al. (2016)
560. Tobacco promotion and smoking.
561. Roditis et al. (2016)
562. Perception of risks of e-cigs.
563. Roditis and Halpern-Felsher (2015)
564. Perceptions of risks of e-cigs.
565. Rodriguez, Parron, and Alarcon (2017)
566. Not English. Only the title/abstract is in English.
567. Rogers (2014)
568. Not relevant.
569. Rom et al. (2015)
570. E-cigs safeness.
571. Rooke and Amos (2014)
572. Media coverage of e-cig.
573. Rosbrook et al. (2017)
574. The effect of sucralose on flavor sweetness in e-cigs. Technical.
575. Rose et al. (2014)
576. Availability of e-cig in the retailer stores.
577. Rowell et al. (2017)
578. Technical. Flavor e-cig and its health implications.
579. Rther et al. (2014)
580. Not relevant.
581. Ruether et al. (2016)
582. E-cig users’ attitudes towards e-cig. None of the e-cig attributes that are the focus of our study mentioned in the study.
583. Rutten et al. (2015)
584. Reasons to use e-cig other than e-cig attributes.
585. Saddleson et al. (2016)
586. Nothing about e-cig attributes preference by users.
587. Saddleson (2015)
588. Dissertation.
589. Sanders‐Jackson et al. (2015)
590. Knowledge of e-cig regulations and constitutions.
591. Sanders-Jackson et al. (2015)
592. Warning statements effectiveness on reduction of e-cig use.
593. Schneller et al. (2017)
     1. Pereference for mint snus flavor in cig not e-cig.
594. Schmidt et al. (2014)
     1. Not preferences for different attributes of e-cig which are the focus of our study.
595. Schmitt et al. (2014)
596. Related to regulation.
597. Schneider and Diehl (2016)
598. Not relevant.
599. Schoenborn and Clarke (2017)
600. Weekly report.
601. Sears et al. (2017)
602. Perception on flavor safteness.
603. Seto, Davis, and Taira (2016)
604. Demographics and e-cig use association.
605. Shah, Paliwal, and Holdford (2017)
606. Not relevant.
607. Sharfstein (2015)
608. Not relevant.
609. Sherratt et al. (2016)
610. E-cig and smoking cessation perception.
611. Shih et al. (2017)
612. Not relevant.
613. Shu-Hong et al. (2014)
614. Technical, not relevant. Use in our study as motivation, not in the review.
615. Siegel, Tanwar, and Wood (2011)
616. The effectiveness of e-cigarettes for smoking cessation.
617. Simonavicius et al. (2017)
618. Short communication.
619. Singh, Kennedy, et al. (2016)
620. Weekly report.
621. Singh, Marynak, et al. (2016)
622. Exposer to e-cig advertisement.
623. Weekly report.
624. Smiley et al. (2017)
625. Not relevant.
626. Smith et al. (2015)
627. Association between perception and advertising.
628. Snider, Cummings, and Bickel (2017)
629. Demand for e-cig and frequency of use.
630. Sokolovsky (2017)
631. Seminar.
632. Soneji et al. (2017)
633. Letter.
634. Soule et al. (2017)
635. Not relevant.
636. Soule, Nasim, and Rosas (2016)
637. Negative experiences using e-cig.
638. Spears et al. (2016)
639. E-cig users and Adults with mental health conditions
640. Spindle et al. (2017)
641. E-cig and cig use relationship.
642. Stanbrook (2016)
643. Editorial.
644. Stein et al. (2015)
645. Attitudes and knowledge of e-cig.
646. Stenger and Chailleux (2016)
647. Not relevant.
648. Stoklosa, Drope, and Chaloupka (2016)
649. Price elasticity of e-cig demand.
650. Strong et al. (2015)
651. Gender and overweight in e-cig users.
652. Surís, Berchtold, and Akre (2015)
653. Users distribution and characteristics.
654. The study was done in Switzerland
655. Sussan et al. (2017)
656. E-cig users behavior and reasons for initiation. Not relevant.
657. Sutfin et al. (2013)
658. E-cig use and users characteristics.
659. Sutfin et al. (2015)
660. Not relevant. E-cig users and past smoking relationship.
661. Sutherland et al. (2016)
662. E-cig use patterns among illicit drug users.
663. Syamlal, King, and Mazurek (2017)
664. Weekly report.
665. Talih et al. (2015)
666. Technical on nicotine yield. Not related to e-cig nicotine level preference.
667. Tamimi (2017)
668. E-cig perceived risks and benefits by smokers and non-smokers.
669. Tan and Bigman (2014)
670. Awareness and harm perception.
671. Tan and Bigman (2016)
672. Correction.
673. Tan, Bigman, and Sanders-Jackson (2015)
674. Exposer to different types of e-cig communications and e-cig usage relationship.
675. Tan, Lee, and Bigman (2016)
676. Factors associated with e-cig use other than e-cig attributes.
677. Temple et al. (2017)
678. E-cig use relationship with other tobacco products use.
679. E-cig users demographics.
680. Thatcher (2015)
681. News.
682. Thirlway (2015)
683. Letters.
684. Thrasher et al. (2016)
685. Perceptions and beliefs towards e-cig.
686. The study was done in Mexico.
687. Thrul and Ramo (2017)
688. Not relevant. Quitting and social media.
689. Tomashefski (2016)
690. Systematic review.
691. E-cig users perception of harm.
692. Torjesen (2016)
693. News.
694. Trtchounian and Talbot (2011)
695. Related to regulation and e-cig use.
696. Trtchounian, Williams, and Talbot (2010)
697. Smoking characteristics of cig and e-cig.
698. Trumbo and Harper (2013)
699. Users perception and awareness. Not related to e-cig attributes.
700. Trumbo and Harper (2015)
701. Related to the advertisement.
702. Trumbo and Kim (2015)
703. Related to the advertisement.
704. Tuchman (2016)
705. Related to the advertisement.
706. Not peer reviewed.
707. Twyman et al. (2016)
708. Attitudes and perception other than e-cig attributes.
709. Unger et al. (2017)
710. Related to regulations.
711. Unger, Soto, and Leventhal (2016)
712. Short communication.
713. Urrutia-Pereira et al. (2017)
714. Smoking in general, not specific to e-cig.
715. Valero-Juan and Suarez del Arco (2014)
716. Non-English.
717. van der Tempel et al. (2016)
718. E-cig use and smoking cessation conversions from Twitter.
719. Vansickel and Eissenberg (2013)
720. Technical. Not relevant.
721. Vardavas, Filippidis, and Agaku (2015)
722. E-cig users’ distribution and the relationship between e-cig use and past smoking behavior.
723. Varlet et al. (2015)
724. Toxicity assesment.
725. Technical.
726. Vasiljevic, Petrescu, and Marteau (2016)
727. Effect of advertisement on candy like e-cig preference. Not relevant to consumers preference for different flavors.
728. Veliz et al. (2017)
729. Type of physical activities that reduce e-cig harm among users.
730. Venkatesan (2017)
731. News.
732. Vickerman, Beebe, et al. (2017)
733. Not relevant, reasons for using e-cig, e-cig attributes not included.
734. Vickerman, Schauer, et al. (2017)
735. Not relevant, reasons for using e-cig, e-cig attributes not included.
736. Villanti, Pearson, et al. (2017)
737. E-cig and cig use relationship.
738. Villanti et al. (2016)
739. Impact of exposer to e-cig advertisement.
740. Voigt (2015)
741. Not relevant, related to regulation need.
742. Volesky et al. (2016)
743. Reasons for using e-cigs, e-cig attributes not included.
744. Wackowski and Delnevo (2016)
745. E-cig users risk perceptions.
746. Wackowski, Delnevo, and Pearson (2015)
747. Letter.
748. Wada et al. (2017)
749. Comparing English and non-English speakers e-cig use patterns.
750. Wadsworth et al. (2016)
751. Not related to e-cig attributes. More on e-cig users’ attitudes.
752. Wagener, Siegel, and Borrelli (2012)
753. Not relevant.
754. Wagner, Camerota, and Propper (2017)
755. E-cig harms and /or benefits perceptions in pregnant women.
756. Wang et al. (2014)
757. Awareness.
758. Wang, Wang, et al. (2016)
759. E-cig uses distribution and probability of using e-cigs among different groups.
760. Wang, Ho, et al. (2015)
761. Short Communication
762. Wang, Ho, et al. (2016)
763. E-cig users’ in hong kong distribution and characteristics.
764. Wang, Li, et al. (2015)
765. E-cig users awareness and perception.
766. Wang et al. (2017)
767. E-cig users characteristics.
768. Wang, Wilson, et al. (2016)
769. Immigrants use of e-cig. Summary stat.
770. Wasowicz, Feleszko, and Goniewicz (2015)
771. Expert review.
772. Waters et al. (2017)
773. Perceptions of harms by demographics
774. Weaver, Kemp, et al. (2017)
775. Health impacts of e-cig nicotine.
776. Weaver, Kim, et al. (2017)
777. Commentary.
778. Weaver et al. (2016)
779. Awareness.
780. Webb Hooper and Kolar (2016)
781. Demographics relationship with e-cig use.
782. Webb Hooper and Kolar (2017)
783. Demographics relationship with e-cig use.
784. Werse et al. (2017)
785. Non-English, the only title is English.
786. Westling et al. (2017)
787. E-cig users distribution by their characteristics.
788. White et al. (2015)
789. E-cig users characteristics and distribution.
790. The study was done in New Zealand.
791. Willemsen et al. (2015)
792. Not-English.
793. Williams, Ghai, and Talbot (2015)
794. characterize the performance of disposable button activated and disposable airflow-activated e-cigs.
795. Williams and Knight (2015)
796. Insights in Public Health
797. Williams (2015)
798. Not relevant.
799. Willis, Haught, and Morris II (2016)
800. E-cigs advertisement.
801. Wills et al. (2017)
802. How e-cigarette use among adolescents is related to subsequent smoking behavior.
803. Wills and Sargent (2017)
804. Commentary.
805. Wills et al. (2016)
806. E-cig users willingness to smoke.
807. Wilson and Wang (2017)
808. Users characteristics and distribution.
809. Winickoff and Winickoff (2016)
810. Summary. reasons that students use e-cig more than other groups. Not relevant to users’ preference for e-cig attributes.
811. Wise (2014)
812. News.
813. Wong et al. (2016)
814. E-cig perception and reasons to use. E-cig attributes did not include in this study.
815. The study is done in Malaysia.
816. Wong et al. (2017)
817. Attitudes and perceptions.
818. Xu et al. (2016)
819. E-cig users’ awareness.
820. Yang et al. (2016)
821. E-cig users’ distribution. Studies the differences in ever and current use of e-cigarettes among non-U.S. citizens, naturalized U.S. citizens, and U.S. natives.
822. Yao et al. (2017)
823. Not relevant. Studies the relationship between spending on e-cig and disease symptoms.
824. Yong et al. (2015)
825. E-cig awareness among users. Comapriview summary statistics among different type of users.
826. Yu and Lippert (2017)
827. E-cig users’ characteristics, demographics.
828. Zarobkiewicz et al. (2016)
829. E-cig users’ distribution among university students.
830. Zhan et al. (2017)
831. E-cig users’ discusion in social media .
832. They mentioned flavor was one of the main topics to discuss in social media but did not mention which flavors preferred by e-cig users.
833. Zhang and Pu (2016)
834. Exposer to smoking at home and e-cig use relationship.
835. Zheng et al. (2017)
836. Analysis of price elasticity of demand, substitution elasticity between different types of tobacco products including e-cig.
837. Zhong et al. (2016)
838. Yong adults intention to smoke e-cigs. Comparative summary statistics.
839. Meta-analysis of six studies.
840. Zhou et al. (2015)
841. This study investigated medical students’ use, knowledge, and beliefs about cigarettes and ATPs.
842. Zhu et al. (2013)
843. Users’ characteristics and distribution.
844. Zhu et al. (2014)
845. examines how the online market for e-cig has changed over time.
846. Zhu et al. (2017)
847. examined whether the increase in the use of e-cigs in the USA was associated with a change in overall smoking cessation rate at the population level.
848. Zhuang et al. (2016)
849. Difference between long term and short term e-cig users. Attributes of e-cig are not considered.

# **References**

1. Abo-Elkheir OI, Sobh E. Knowledge about electronic cigarettes and its perception: a community survey, Egypt. Respiratory Research. 2016;17(1):1-7. Epub 2016/05/18. doi: 10.1186/s12931-016-0365-0. PubMed PMID: 27183972; PubMed Central PMCID: PMCPMC4869382.

2. Agaku IT, Davis K, Patel D, Shafer P, Cox S, Ridgeway W, et al. A longitudinal study of the relationship between receptivity to e-cigarette advertisements and e-cigarette use among baseline non-users of cigarettes and e-cigarettes, United States. Tobacco Induced Diseases. 2017;15:1-7. doi: 10.1186/s12971-017-0145-8. PubMed PMID: WOS:000414609800001.

3. Ahern NR, Mechling B. E-cigarettes: a rising trend among youth. Journal of psychosocial nursing and mental health services. 2014;52(6):27-31. Epub 2014/05/13. doi: 10.3928/02793695-20140506-01. PubMed PMID: 24815950.

4. Aherrera A, Olmedo P, Grau-Perez M, Tanda S, Goessler W, Jarmul S, et al. The association of e-cigarette use with exposure to nickel and chromium: A preliminary study of non-invasive biomarkers. Environmental Research. 2017;159:313-20. doi: 10.1016/j.envres.2017.08.014. PubMed PMID: WOS:000413280500035.

5. Ajjandaleh H, Bolze C, Khoury FEL, Melchior M, Mary-Krause M. What are the factors for electronic cigarette use in French young adults? European Journal of Public Health. 2017;27. PubMed PMID: WOS:000414389804200.

6. Akre C, Suris J-C. Adolescents and young adults' perceptions of electronic cigarettes as a gateway to smoking: a qualitative study in Switzerland. International Journal of Environmental Research and Public Health. 2017;32(5):448-54. doi: 10.1093/her/cyx054. PubMed PMID: 125250987. Language: English. Entry Date: 20170926. Revision Date: 20171108. Publication Type: Article.

7. Alawsi F, Nour R, Prabhu S. Are e-cigarettes a gateway to smoking or a pathway to quitting? British dental journal. 2015;219(3):111-5. Epub 2015/08/15. doi: 10.1038/sj.bdj.2015.591. PubMed PMID: 26271862.

8. Alcalá HE, Albert SL, Ortega AN. E-cigarette use and disparities by race, citizenship status and language among adolescents. Addictive Behaviors. 2016;57:30-4. doi: 10.1016/j.addbeh.2016.01.014. PubMed PMID: 113373711. Language: English. Entry Date: 20161223. Revision Date: 20170604. Publication Type: journal article. Journal Subset: Biomedical.

9. Al-Delaimy WK, Myers MG, Strong DR. E-cigarettes are losing ground among smokers and non-smokers. American Journal of Public Health. 2015;105(11):e1-2. Epub 2015/09/18. doi: 10.2105/ajph.2015.302896. PubMed PMID: 26378864; PubMed Central PMCID: PMCPMC4605184.

10. Alexander JP, Coleman BN, Johnson SE, Tessman GK, Tworek C, Dickinson DM. Smoke and Vapor: Exploring the Terminology Landscape among Electronic Cigarette Users. Tobacco Regulatory Science. 2016;2(3):204-13. PubMed PMID: 27430008.

11. Alexander LEC, Vyas A, Schraufnagel DE, Malhotra A. Electronic cigarettes: the new face of nicotine delivery and addiction. Journal of Thoracic Disease. 2015;7(8):E248-E51. doi: 10.3978/j.issn.2072-1439.2015.07.37. PubMed PMID: WOS:000362069800014.

12. Allem JP, Forster M, Neiberger A, Unger JB. Characteristics of emerging adulthood and e-cigarette use: Findings from a pilot study. Addictive Behaviors. 2015;50:40-4. Epub 2015/06/22. doi: 10.1016/j.addbeh.2015.06.023. PubMed PMID: 26093505; PubMed Central PMCID: PMCPMC4515365.

13. Allem J-P, Unger JB, Garcia R, Baezconde-Garbanati L, Sussman S. Tobacco attitudes and behaviors of vape shop retailers in Los Angeles. American Journal of Health Behavior. 2015;39(6):794-8. doi: 10.5993/ajhb.39.6.7. PubMed PMID: 2016-21518-007.

14. Allen JG, Flanigan SS, LeBlanc M, Vallarino J, MacNaughton P, Stewart JH, et al. Flavoring Chemicals in E-Cigarettes: Diacetyl, 2,3-Pentanedione, and Acetoin in a Sample of 51 Products, Including Fruit-, Candy-, and Cocktail-Flavored E-Cigarettes. Environmental health perspectives. 2016;124(6):733-9. Epub 2015/12/09. doi: 10.1289/ehp.1510185. PubMed PMID: 26642857; PubMed Central PMCID: PMCPMC4892929.

15. Amato MS, Boyle RG, Levy D. How to define e-cigarette prevalence? Finding clues in the use frequency distribution. Tobacco Control. 2016;25(e1):e24-9. Epub 2015/06/19. doi: 10.1136/tobaccocontrol-2015-052236. PubMed PMID: 26085124; PubMed Central PMCID: PMCPMC4683118.

16. Ambrose BK, Day HR, Rostron B, Conway KP, Borek N, Hyland A, et al. Flavored tobacco product use among US youth aged 12-17 years, 2013-2014. JAMA. 2015;314(17):1871-3.

17. Ambrose BK, Rostron BL, Johnson SE, Portnoy DB, Apelberg BJ, Kaufman AR, et al. Perceptions of the relative harm of cigarettes and e-cigarettes among U.S. youth. American Journal of Preventive Medicine. 2014;47(2, Suppl 1):S53-S60. doi: 10.1016/j.amepre.2014.04.016. PubMed PMID: 2014-31191-009.

18. Amrock SM, Lee L, Weitzman M. Perceptions of e-cigarettes and noncigarette tobacco products among US youth. Pediatrics. 2016;138(5):1-10. PubMed PMID: 2017-01396-001.

19. Amrock SM, Zakhar J, Zhou S, Weitzman M. Perception of e-cigarette harm and its correlation with use among U.S. adolescents. Nicotine & Tobacco Research. 2015;17(3):330-6. doi: 10.1093/ntr/ntu156. PubMed PMID: 2015-08695-009.

20. Anand V, McGinty KL, O'Brien K, Guenthner G, Hahn E, Martin CA. E-cigarette use and beliefs among urban public high school students in North Carolina. Journal of Adolescent Health. 2015;57(1):46-51. doi: 10.1016/j.jadohealth.2015.03.018. PubMed PMID: 2015-44086-009.

21. Andler R, Guignard R, Wilquin J-L, Beck F, Richard J-B, Nguyen-Thanh V. Electronic cigarette use in France in 2014. International Journal of Public Health. 2016;61(2):159-65. doi: 10.1007/s00038-015-0773-9. PubMed PMID: 2015-58039-001.

22. Arane K, Goldman RD. Electronic cigarettes and adolescents. Canadian Family Physician. 2016;62(11):897-8. PubMed PMID: WOS:000388737400014.

23. Arrazola RA, Singh T, Corey CG, Husten CG, Neff LJ, Apelberg BJ, et al. Tobacco Use Among Middle and High School Students — United States, 2011–2014. MMWR Morb Mortal Wkly Rep: 2015.

24. Ashford K, Rayens E, Wiggins AT, Rayens MK, Fallin A, Sayre MM. Advertising exposure and use of e-cigarettes among female current and former tobacco users of childbearing age. Public Health Nursing. 2017;34(5):430-6. doi: 10.1111/phn.12334. PubMed PMID: WOS:000411189100004.

25. Ashford K, Wiggins A, Butler K, Ickes M, Rayens MK, Hahn E. E-cigarette use and perceived harm among women of childbearing age who reported tobacco use during the past year. Nursing Research. 2016;65(5):408-14. doi: 10.1097/nnr.0000000000000176. PubMed PMID: 2016-45301-009.

26. Aszyk J, Wozniak MK, Kubica P, Kot-Wasik A, Namiesnik J, Wasik A. Comprehensive determination of flavouring additives and nicotine in e-cigarette refill solutions. Part II: Gas-chromatography-mass spectrometry analysis. Journal of Chromatography A. 2017;1517:156-64. doi: 10.1016/j.chroma.2017.08.057. PubMed PMID: WOS:000411771500017.

27. Audrain-McGovern J, Strasser AA, Wileyto EP. The impact of flavoring on the rewarding and reinforcing value of e-cigarettes with nicotine among young adult smokers. Drug and Alcohol Dependence. 2016;166:263-7. doi: 10.1016/j.drugalcdep.2016.06.030. PubMed PMID: 2016-35218-001.

28. Awan KH. Experimentation and correlates of electronic nicotine delivery system (electronic cigarettes) among university students - A cross sectional study. Saudi Dental Journal. 2016;28(2):91-5. doi: 10.1016/j.sdentj.2015.12.002. PubMed PMID: WOS:000382176200006.

29. Ayers J, Allem J-P. WHY DO PEOPLE USE ELECTRONIC NICOTINE DELIVERY SYSTEMS ( ELECTRONIC CIGARETTES)? A CONTENT ANALYSIS OF TWITTER, 2012-2015. Annals of Behavioral Medicine. 2017;51:S862-S. PubMed PMID: WOS:000398947201102.

30. Ayers JW, Althouse BM, Allem J-P, Leas EC, Dredze M, Williams RS. Revisiting the Rise of Electronic Nicotine Delivery Systems Using Search Query Surveillance. American Journal of Preventive Medicine. 2016;50(6):E173-E81. doi: 10.1016/j.amepre.2015.12.008. PubMed PMID: WOS:000376509900002.

31. Azagba S, Baskerville NB, Foley K. Susceptibility to cigarette smoking among middle and high school e-cigarette users in Canada. Preventive Medicine. 2017;103:14-9. doi: 10.1016/j.ypmed.2017.07.017. PubMed PMID: WOS:000411483200003.

32. Babineau K, Taylor K, Clancy L. Electronic Cigarette Use among Irish Youth: A Cross Sectional Study of Prevalence and Associated Factors. PLOS ONE. 2015;10(5):e0126419. Epub 2015/05/29. doi: 10.1371/journal.pone.0126419. PubMed PMID: 26018542; PubMed Central PMCID: PMCPMC4446031.

33. Baeza-Loya S, Viswanath H, Carter A, Molfese DL, Velasquez KM, Baldwin PR, et al. Perceptions about e-cigarette safety may lead to e-smoking during pregnancy. Bulletin of the Menninger Clinic. 2014;78(3):243-52. Epub 2014/09/24. doi: 10.1521/bumc.2014.78.3.243. PubMed PMID: 25247743; PubMed Central PMCID: PMCPMC4458373.

34. Baggett TP, Campbell EG, Chang Y, Rigotti NA. Other tobacco product and electronic cigarette use among homeless cigarette smokers. Addictive Behaviors. 2016;60:124-30. doi: 10.1016/j.addbeh.2016.04.006. PubMed PMID: WOS:000377731500021.

35. Ballbe M, Martinez-Sanchez JM, Sureda X, Fu M, Perez-Ortuno R, Pascual JA, et al. Cigarettes vs. e-cigarettes: Passive exposure at home measured by means of airborne marker and biomarkers. Environmental Research. 2014;135:76-80. Epub 2014/09/30. doi: 10.1016/j.envres.2014.09.005. PubMed PMID: 25262078.

36. Bandiera FC, Loukas A, Wilkinson AV, Perry CL. Associations between tobacco and nicotine product use and depressive symptoms among college students in Texas. Addictive Behaviors. 2016;63:19-22. doi: 10.1016/j.addbeh.2016.06.024. PubMed PMID: 2016-41608-004.

37. Barnett TE, Soule EK, Forrest JR, Porter L, Tomar SL. Adolescent Electronic Cigarette Use Associations With Conventional Cigarette and Hookah Smoking. American Journal of Preventive Medicine. 2015;49(2):199-206. doi: 10.1016/j.amepre.2015.02.013. PubMed PMID: WOS:000357989000006.

38. Barrington-Trimis JL, Samet JM, McConnell R. Flavorings in electronic cigarettes: an unrecognized respiratory health hazard? JAMA. 2014;312(23):2493-4. Epub 2014/11/11. doi: 10.1001/jama.2014.14830. PubMed PMID: 25383564; PubMed Central PMCID: PMCPMC4361011.

39. Barrington-Trimis JL, Urman R, Berhane K, Unger JB, Cruz TB, Pentz MA, et al. E-Cigarettes and Future Cigarette Use. Pediatrics. 2016;138(1). Epub 2016/06/15. doi: 10.1542/peds.2016-0379. PubMed PMID: 27296866; PubMed Central PMCID: PMCPMC4925085.

40. Barrington-Trimis JL, Urman R, Leventhal AM, Gauderman WJ, Cruz TB, Gilreath TD, et al. E-cigarettes, Cigarettes, and the Prevalence of Adolescent Tobacco Use. Pediatrics. 2016;138(2). doi: 10.1542/peds.2015-3983. PubMed PMID: WOS:000381002500010.

41. Basch CH, Mongiovi J, Hillyer GC, Ethan D, Hammond R. An analysis of electronic cigarette and cigarette advertising in US women's magazines. International Journal of Preventive Medicine. 2016;7.

42. Bauhoff S, Montero A, Scharf D. Perceptions of e-cigarettes: A comparison of adult smokers and non-smokers in a Mechanical Turk sample. The American Journal of Drug and Alcohol Abuse. 2017;43(3):311-23. doi: 10.1080/00952990.2016.1207654. PubMed PMID: 2017-19354-011.

43. Bauld L. Electronic cigarettes and smoking cessation. Nicotine & Tobacco Research. 2016;18(10):1925-. doi: 10.1093/ntr/ntw207. PubMed PMID: 2016-46012-001.

44. Bauld L, MacKintosh AM, Eastwood B, Ford A, Moore G, Dockrell M, et al. Young People's Use of E-Cigarettes across the United Kingdom: Findings from Five Surveys 2015-2017. International Journal of Environmental Research and Public Health. 2017;14(9). doi: 10.3390/ijerph14090973. PubMed PMID: WOS:000411574400025.

45. Bauld L, MacKintosh AM, Ford A, McNeill A. E-cigarette uptake amongst UK youth: Experimentation, but little or no regular use in nonsmokers. Nicotine & Tobacco Research. 2016;18(1):102-3. PubMed PMID: 2015-57108-017.

46. Baumann AW, Kohler C, Kim YI, Cheong J, Hendricks P, Bailey WC, et al. Differences in Electronic Cigarette Awareness, Use History, and Advertisement Exposure Between Black and White Hospitalized Cigarette Smokers. Journal of Cancer Education. 2015;30(4):648-54. Epub 2014/12/17. doi: 10.1007/s13187-014-0767-y. PubMed PMID: 25503053; PubMed Central PMCID: PMCPMC4466094.

47. Baweja R, Curci KM, Yingst J, Veldheer S, Hrabovsky S, Wilson SJ, et al. Views of experienced electronic cigarette users. Addiction Research & Theory. 2016;24(1):80-8. doi: 10.3109/16066359.2015.1077947. PubMed PMID: 2016-08848-011.

48. Beard E, West R, Michie S, Brown J. Association between electronic cigarette use and changes in quit attempts, success of quit attempts, use of smoking cessation pharmacotherapy, and use of stop smoking services in England: Time series analysis of population trends. BMJ. 2016;354. PubMed PMID: 2016-44842-001.

49. Benowitz NL, Donny EC, Hatsukami DK. Reduced nicotine content cigarettes, e-cigarettes and the cigarette end game. Addiction. 2017;112(1):6-7. doi: 10.1111/add.13534. PubMed PMID: WOS:000393904300002.

50. Berg CJ. Preferred flavors and reasons for e-cigarette use and discontinued use among never, current, and former smokers. International Journal of Public Health. 2016;61(2):225-36. doi: 10.1007/s00038-015-0764-x. PubMed PMID: WOS:000373180100011.

51. Berg CJ, Haardoerfer R, Escoffery C, Zheng P, Kegler M. Cigarette users’ interest in using or switching to electronic nicotine delivery systems for smokeless tobacco for harm reduction, cessation, or novelty: A cross-sectional survey of US adults. Nicotine & Tobacco Research. 2015;17(2):245-55. doi: 10.1093/ntr/ntu103. PubMed PMID: 2015-03033-020.

52. Berg CJ, Haardörfer R, Schauer G, Betelihem G, Masters M, McDonald B, et al. Reasons for Polytobacco Use among Young Adults: Scale Development and Validation. Tobacco prevention & cessation. 2016;2.

53. Berg CJ, Stratton E, Schauer GL, Lewis M, Wang Y, Windle M, et al. Perceived harm, addictiveness, and social acceptability of tobacco products and marijuana among young adults: Marijuana, hookah, and electronic cigarettes win. Substance Use & Misuse. 2015;50(1):79-89. doi: 10.3109/10826084.2014.958857. PubMed PMID: 2014-52360-010.

54. Biener L, Lee Hargraves J. A longitudinal study of electronic cigarette use among a population-based sample of adult smokers: Association with smoking cessation and motivation to quit. Nicotine & Tobacco Research. 2015;17(2):127-33. doi: 10.1093/ntr/ntu200. PubMed PMID: 2015-03033-002.

55. Biener L, Song E, Sutfin EL, Spangler J, Wolfson M. Electronic Cigarette Trial and Use among Young Adults: Reasons for Trial and Cessation of Vaping. International Journal of Environmental Research and Public Health. 2015;12(12):16019-26. doi: 10.3390/ijerph121215039. PubMed PMID: 26694438; PubMed Central PMCID: PMCPMC4690975.

56. Bitzer ZT, Goel R, Reilly SM, Elias RJ, Foulds JT, Muscat JT, et al. Effect of Flavoring Chemicals on Free Radical Formation in Electronic Cigarette Aerosols. Free Radical Biology and Medicine. 2017;112:200-. doi: 10.1016/j.freeradbiomed.2017.10.315. PubMed PMID: WOS:000415805100324.

57. Bold KW, Kong G, Cavallo DA, Camenga DR, Krishnan-Sarin S. Reasons for Trying E-cigarettes and Risk of Continued Use. Pediatrics. 2016;138(3). Epub 2016/08/10. doi: 10.1542/peds.2016-0895. PubMed PMID: 27503349; PubMed Central PMCID: PMCPMC5005025 conflicts of interest to disclose.

58. Bold KW, Morean ME, Kong G, Simon P, Camenga DR, Cavallo DA, et al. Early age of e-cigarette use onset mediates the association between impulsivity and e-cigarette use frequency in youth. Drug and Alcohol Dependence. 2017;181:146-51. doi: 10.1016/j.drugalcdep.2017.09.025. PubMed PMID: WOS:000416496600022.

59. Bonhomme MG, Holder-Hayes E, Ambrose BK, Tworek C, Feirman SP, King BA, et al. Flavoured non-cigarette tobacco product use among US adults: 2013–2014. Tobacco Control. 2016;25(Suppl 2):4-13. doi: 10.1136/tobaccocontrol-2016-053373. PubMed PMID: 2016-61125-002.

60. Booth P, Albery IP, Frings D. Effect of e-cigarette advertisements and antismoking messages on explicit and implicit attitudes towards tobacco and e-cigarette smoking in 18-65-year-olds: a randomised controlled study protocol. BMJ open. 2017;7(6). doi: 10.1136/bmjopen-2016-014361. PubMed PMID: WOS:000406391200058.

61. Borderud SP, Li Y, Burkhalter JE, Sheffer CE, Ostroff JS. Electronic cigarette use among patients with cancer: characteristics of electronic cigarette users and their smoking cessation outcomes. Cancer. 2014;120(22):3527-35. Epub 2014/09/25. doi: 10.1002/cncr.28811. PubMed PMID: 25252116; PubMed Central PMCID: PMCPMC5642904.

62. Bostean G, Crespi CM, Vorapharuek P, McCarthy WJ. E-cigarette use among students and e-cigarette specialty retailer presence near schools. Health & Place. 2016;42:129-36. doi: 10.1016/j.healthplace.2016.09.012. PubMed PMID: 2016-58372-017.

63. Bostean G, Trinidad DR, McCarthy WJ. E-cigarette use among never-smoking California students. American Journal of Public Health. 2015;105(12):2423-5. doi: 10.2105/ajph.2015.302899. PubMed PMID: 2015-51572-009.

64. Boulay M-E, Henry C, Bosse Y, Boulet L-P, Morissette MC. Acute effects of nicotine-free and flavour-free electronic cigarette use on lung functions in healthy and asthmatic individuals. Respiratory Research. 2017;18. doi: 10.1186/s12931-017-0518-9. PubMed PMID: WOS:000396086100001.

65. Breheny D, Adamson J, Azzopardi D, Baxter A, Bishop E, Carr T, et al. A novel hybrid tobacco product that delivers a tobacco flavour note with vapour aerosol (Part 2): In vitro biological assessment and comparison with different tobacco-heating products. Food and Chemical Toxicology. 2017;106:533-46. doi: 10.1016/j.fct.2017.05.023. PubMed PMID: WOS:000405160600054.

66. Breland AB, Spindle T, Weaver M, Eissenberg T. Science and electronic cigarettes: current data, future needs. Journal of Addiction Medicine. 2014;8(4):223-33. Epub 2014/08/05. doi: 10.1097/adm.0000000000000049. PubMed PMID: 25089952; PubMed Central PMCID: PMCPMC4122311.

67. Brikmanis K, Petersen A, Doran N. E-Cigarette Use, Perceptions, and Cigarette Smoking Intentions in a Community Sample of Young Adult Nondaily Cigarette Smokers. Psychology of Addictive Behaviors. 2017;31(3):336-42. doi: 10.1037/adb0000257. PubMed PMID: WOS:000400899700011.

68. Brose LS, Hitchman SC, Brown J, West R, McNeill A. Is the use of electronic cigarettes while smoking associated with smoking cessation attempts, cessation and reduced cigarette consumption? A survey with a 1‐year follow‐up. Addiction. 2015;110(7):1160-8. doi: 10.1111/add.12917. PubMed PMID: 2015-18665-001.

69. Brown CJ, Cheng JM. Electronic cigarettes: product characterisation and design considerations. Tobacco Control. 2014;23:4-10. doi: 10.1136/tobaccocontrol-2013-051476. PubMed PMID: WOS:000334635400002.

70. Brown J, Beard E, Kotz D, Michie S, West R. Real-world effectiveness of e-cigarettes when used to aid smoking cessation: a cross-sectional population study. Addiction. 2014;109(9):1531-40. Epub 2014/05/23. doi: 10.1111/add.12623. PubMed PMID: 24846453; PubMed Central PMCID: PMCPMC4171752.

71. Brown J, West R, Beard E, Michie S, Shahab L, McNeill A. Prevalence and characteristics of e-cigarette users in Great Britain: Findings from a general population survey of smokers. Addictive Behaviors. 2014;39(6):1120-5. doi: 10.1016/j.addbeh.2014.03.009. PubMed PMID: 103817207. Language: English. Entry Date: 20150116. Revision Date: 20161125. Publication Type: journal article.

72. Browne M, Todd DG. Then and now: Consumption and dependence in e-cigarette users who formerly smoked cigarettes. Addictive Behaviors. 2018;76:113-21. doi: 10.1016/j.addbeh.2017.07.034. PubMed PMID: WOS:000412959900017.

73. Buchting FO, Emory KT, Scout, Kim Y, Fagan P, Vera LE, et al. Transgender use of cigarettes, cigars, and e-cigarettes in a national study. American Journal of Preventive Medicine. 2017;53(1):e1-e7. doi: 10.1016/j.amepre.2016.11.022. PubMed PMID: 2017-27580-017.

74. Bullen C. Rise in e-cigarette use linked to increase in smoking cessation rates. BMJ. 2017;358:j3506. Epub 2017/07/28. doi: 10.1136/bmj.j3506. PubMed PMID: 28747300.

75. Bullen C, Howe C, Laugesen M, McRobbie H, Parag V, Williman J, et al. Electronic cigarettes for smoking cessation: A randomised controlled trial. The Lancet. 2013;382(9905):1629-37. doi: 10.1016/s0140-6736(13)61842-5. PubMed PMID: 2013-41203-024.

76. Bullen C, Knight-West O, O'Brien B, Walker N. DETRIMENTAL EFFECTS OF E-CIGARETTES Evidence, not conjecture, should guide clinical practice and policies on e-cigarettes. BMJ. 2014;348. doi: 10.1136/bmj.g2008. PubMed PMID: WOS:000333031700022.

77. Bullen C, McRobbie H, Thornley S, Glover M, Lin R, Laugesen M. Effect of an electronic nicotine delivery device (e cigarette) on desire to smoke and withdrawal, user preferences and nicotine delivery: randomised cross-over trial. Tobacco Control. 2010;19(2):98-103. doi: 10.1136/tc.2009.031567. PubMed PMID: 105201017. Language: English. Entry Date: 20100625. Revision Date: 20150711. Publication Type: Journal Article.

78. Bunnell RE, Agaku IT, Arrazola RA, Apelberg BJ, Caraballo RS, Corey CG, et al. Intentions to Smoke Cigarettes Among Never-Smoking US Middle and High School Electronic Cigarette Users: National Youth Tobacco Survey, 2011-2013. Nicotine & Tobacco Research. 2015;17(2):228-35. doi: 10.1093/ntr/ntu166. PubMed PMID: WOS:000350142300018.

79. Busch AM, Leavens EL, Wagener TL, Buckley ML, Tooley EM. Prevalence, Reasons for Use, and Risk Perception of Electronic Cigarettes Among Post-Acute Coronary Syndrome Smokers. Journal of Cardiopulmonary Rehabilitation and Prevention. 2016;36(5):352-7. doi: 10.1097/hcr.0000000000000179. PubMed PMID: WOS:000384117600006.

80. Bush D, Goniewicz ML. A pilot study on nicotine residues in houses of electronic cigarette users, tobacco smokers, and non-users of nicotine-containing products. International Journal of Drug Policy. 2015;26(6):609-11. Epub 2015/04/15. doi: 10.1016/j.drugpo.2015.03.003. PubMed PMID: 25869751; PubMed Central PMCID: PMCPMC4457620.

81. Camenga DR, Cavallo DA, Kong G, Morean ME, Connell CM, Simon P, et al. Adolescents' and Young Adults' Perceptions of Electronic Cigarettes for Smoking Cessation: A Focus Group Study. Nicotine & Tobacco Research. 2015;17(10):1235-41. doi: 10.1093/ntr/ntv020. PubMed PMID: WOS:000363175500008.

82. Camenga DR, Kong G, Cavallo DA, Krishnan-Sarin S. Current and Former Smokers' Use of Electronic Cigarettes for Quitting Smoking: An Exploratory Study of Adolescents and Young Adults. Nicotine & Tobacco Research. 2017;19(12):1531-5. doi: 10.1093/ntr/ntw248. PubMed PMID: WOS:000414562900018.

83. Cameron JM, Howell DN, White JR, Andrenyak DM, Layton ME, Roll JM. Variable and potentially fatal amounts of nicotine in e-cigarette nicotine solutions. Tobacco Control. 2014;23(1):77-8. doi: 10.1136/tobaccocontrol-2012-050604. PubMed PMID: WOS:000328550800025.

84. Campbell-Heider N, Snow D. Teen Use of Electronic Cigarettes: What Does the Research Tell Us? Journal of addictions nursing. 2016;27(1):56-61. Epub 2016/03/08. doi: 10.1097/jan.0000000000000114. PubMed PMID: 26950845.

85. Caponnetto P, Alamo A, Maglia M, Saitta D, Benfatto F, Polosa R. [Electronic cigarette: current overview and future perspectives]. Epidemiologia e prevenzione. 2014;38(2):138-41. Epub 2014/07/06. PubMed PMID: 24986413.

86. Caraballo RS, Jamal A, Nguyen KH, Kuiper NM, Arrazola RA. Electronic Nicotine Delivery System Use Among U.S. Adults, 2014. American Journal of Preventive Medicine. 2016;50(2):226-9. Epub 2015/12/22. doi: 10.1016/j.amepre.2015.09.013. PubMed PMID: 26687190.

87. Cardenas VM, Breen PJ, Compadre CM, Delongchamp RR, Barone CP, Phillips MM, et al. The smoking habits of the family influence the uptake of e-cigarettes in US children. Annals of epidemiology. 2015;25(1):60-2. Epub 2014/12/03. doi: 10.1016/j.annepidem.2014.09.013. PubMed PMID: 25453726.

88. Cardenas VM, Evans VL, Balamurugan A, Faramawi MF, Delongchamp RR, Wheeler JG. Use of electronic nicotine delivery systems and recent initiation of smoking among US youth. International Journal of Public Health. 2016;61(2):237-41. doi: 10.1007/s00038-015-0783-7. PubMed PMID: 2016-05817-001.

89. Carr ER. E-Cigarettes: Facts, Perceptions, and Marketing Messages. Clinical Journal of Oncology Nursing. 2014;18(1):112-6. doi: 10.1188/14.cjon.112-116. PubMed PMID: WOS:000331594400018.

90. Case K, Crook B, Lazard A, Mackert M. Formative research to identify perceptions of e-cigarettes in college students: Implications for future health communication campaigns. Journal of American College Health. 2016;64(5):380-9. doi: 10.1080/07448481.2016.1158180. PubMed PMID: WOS:000381367000004.

91. Case KR, Harrell MB, Pérez A, Loukas A, Wilkinson AV, Springer AE, et al. The relationships between sensation seeking and a spectrum of e-cigarette use behaviors: Cross-sectional and longitudinal analyses specific to Texas adolescents. Addictive Behaviors. 2017;73:151-7. doi: 10.1016/j.addbeh.2017.05.007. PubMed PMID: 2017-26639-027.

92. Cavalcante TM, Szklo AS, Perez CA, Thrasher JF, Szklo M, Ouimet J, et al. Electronic cigarette awareness, use, and perception of harmfulness in Brazil: findings from a country that has strict regulatory requirements. Cadernos de saude publica. 2017;33Suppl 3(Suppl 3):e00074416. Epub 2017/09/28. doi: 10.1590/0102-311x00074416. PubMed PMID: 28954048.

93. Chaffee BW, Couch ET, Gansky SA. Trends in characteristics and multi-product use among adolescents who use electronic cigarettes, United States 2011-2015. PLOS ONE. 2017;12(5). doi: 10.1371/journal.pone.0177073. PubMed PMID: WOS:000400649500042.

94. Chapman S, Daube M, Maziak W. Should e-cigarette use be permitted in smoke-free public places? No. Tobacco Control. 2016:tobaccocontrol-2016-053359.

95. Chen AT, Zhu SH, Conway M. What Online Communities Can Tell Us About Electronic Cigarettes and Hookah Use: A Study Using Text Mining and Visualization Techniques. Journal of Medical Internet Research. 2015;17(9):e220. Epub 2015/10/01. doi: 10.2196/jmir.4517. PubMed PMID: 26420469; PubMed Central PMCID: PMCPMC4642380.

96. Chen C, Zhuang Y-L, Zhu S-H. E-cigarette design preference and smoking cessation: A U.S. Population study. American Journal of Preventive Medicine. 2016;51(3):356-63. doi: 10.1016/j.amepre.2016.02.002. PubMed PMID: 2016-41616-012.

97. Chen IL. FDA summary of adverse events on electronic cigarettes. Nicotine & Tobacco Research. 2013;15(2):615-6. doi: 10.1093/ntr/nts145. PubMed PMID: 22855883.

98. Cheney MK, Gowin M, Wann TF. Electronic Cigarette Use in Straight-to-Work Young Adults. American Journal of Health Behavior. 2016;40(2):268-79. doi: 10.5993/ajhb.40.2.12. PubMed PMID: WOS:000401113000012.

99. Cho JH, Shin E, Moon S-S. Electronic-Cigarette Smoking Experience Among Adolescents. Journal of Adolescent Health. 2011;49(5):542-6. doi: 10.1016/j.jadohealth.2011.08.001. PubMed PMID: WOS:000296390000016.

100. Choi K, Bernat D. E-cigarette use among Florida youth with and without asthma. American Journal of Preventive Medicine. 2016;51(4):446-53. doi: 10.1016/j.amepre.2016.03.010. PubMed PMID: 2016-46069-008.

101. Choi K, Fabian L, Mottey N, Corbett A, Forster J. Young adults’ favorable perceptions of snus, dissolvable tobacco products, and electronic cigarettes: findings from a focus group study. American Journal of Public Health. 2012;102(11):2088-93.

102. Choi K, Forster J. Characteristics Associated With Awareness, Perceptions, and Use of Electronic Nicotine Delivery Systems Among Young US Midwestern Adults. American Journal of Public Health. 2013;103(3):556-61. doi: 10.2105/ajph.2012.300947. PubMed PMID: WOS:000330762600044.

103. Choi K, Forster JL. Beliefs and Experimentation with Electronic Cigarettes A Prospective Analysis Among Young Adults. American Journal of Preventive Medicine. 2014;46(2):175-8. doi: 10.1016/j.amepre.2013.10.007. PubMed PMID: WOS:000329859200012.

104. Choi K, Grana R, Bernat D. Electronic Nicotine Delivery Systems and Acceptability of Adult Cigarette Smoking Among Florida Youth: Renormalization of Smoking? Journal of Adolescent Health. 2017;60(5):592-8. doi: 10.1016/j.jadohealth.2016.12.001. PubMed PMID: WOS:000401110800017.

105. Chu KH, Unger JB, Allem JP, Pattarroyo M, Soto D, Cruz TB, et al. Diffusion of Messages from an Electronic Cigarette Brand to Potential Users through Twitter. PLOS ONE. 2015;10(12):e0145387. Epub 2015/12/20. doi: 10.1371/journal.pone.0145387. PubMed PMID: 26684746; PubMed Central PMCID: PMCPMC4694088.

106. Clapp PW, Pawlak EA, Lackey JT, Keating JE, Reeber SL, Glish GL, et al. Flavored e-cigarette liquids and cinnamaldehyde impair respiratory innate immune cell function. American Journal of Physiology-Lung Cellular and Molecular Physiology. 2017;313(2):L278-L92. doi: 10.1152/ajplung.00452.2016. PubMed PMID: WOS:000406746900008.

107. Clarke TN, Lusher JM. Willingness to try electronic cigarettes among UK adolescents. Journal of Child & Adolescent Substance Abuse. 2017;26(3):175-82. doi: 10.1080/1067828x.2016.1242098. PubMed PMID: 2017-15455-001.

108. Cobb CO, Hendricks PS, Eissenberg T. Electronic cigarettes and nicotine dependence: evolving products, evolving problems. BMC medicine. 2015;13:119. Epub 2015/05/23. doi: 10.1186/s12916-015-0355-y. PubMed PMID: 25998379; PubMed Central PMCID: PMCPMC4440602.

109. Cobb NK, Sonti R. E-Cigarettes: The Science Behind the Smoke and Mirrors. Respiratory Care. 2016;61(8):1122-8. doi: 10.4187/respcare.04944. PubMed PMID: WOS:000382381800017.

110. Cole-Lewis H, Varghese A, Sanders A, Schwarz M, Pugatch J, Augustson E. Assessing Electronic Cigarette-Related Tweets for Sentiment and Content Using Supervised Machine Learning. Journal of Medical Internet Research. 2015;17(8):e208. Epub 2015/08/27. doi: 10.2196/jmir.4392. PubMed PMID: 26307512; PubMed Central PMCID: PMCPMC4642404.

111. Coleman BN. The association between electronic cigarette use and cigarette smoking behavior among young adults in the United States. US: ProQuest Information & Learning; 2016.

112. Coleman BN, Apelberg BJ, Ambrose BK, Green KM, Choiniere CJ, Bunnell R, et al. Association Between Electronic Cigarette Use and Openness to Cigarette Smoking Among US Young Adults. Nicotine & Tobacco Research. 2015;17(2):212-8. doi: 10.1093/ntr/ntu211. PubMed PMID: WOS:000350142300016.

113. Coleman BN, Johnson SE, Tessman GK, Tworek C, Alexander J, Dickinson DM, et al. 'it's not smoke. It's not tar. It's not 4000 chemicals. Case closed': Exploring attitudes, beliefs, and perceived social norms of e-cigarette use among adult users. Drug and Alcohol Dependence. 2016;159:80-5. doi: 10.1016/j.drugalcdep.2015.11.028. PubMed PMID: 2015-57763-001.

114. Cooke A, Fergeson J, Bulkhi A, Casale TB. The Electronic Cigarette: The Good, the Bad, and the Ugly. Journal of Allergy and Clinical Immunology: In Practice. 2015;3(4):498-505. Epub 2015/07/15. doi: 10.1016/j.jaip.2015.05.022. PubMed PMID: 26164573.

115. Cooper M, Case KR, Loukas A. E-cigarette use among Texas youth: Results from the 2014 Texas Youth Tobacco Survey. Addictive Behaviors. 2015;50:173-7. doi: 10.1016/j.addbeh.2015.06.034. PubMed PMID: 109606494. Language: English. Entry Date: 20150923. Revision Date: 20161203. Publication Type: journal article. Journal Subset: Biomedical.

116. Cooper M, Case KR, Loukas A, Creamer MR, Perry CL. E-cigarette dual users, exclusive users and perceptions of tobacco products. American Journal of Health Behavior. 2016;40(1):108-16. doi: 10.5993/ajhb.40.1.12. PubMed PMID: 2016-21516-012.

117. Cooper M, Creamer MR, Ly C, Crook B, Harrell MB, Perry CL. Social norms, perceptions and dual/poly tobacco use among Texas youth. American Journal of Health Behavior. 2016;40(6):761-70. doi: 10.5993/ajhb.40.6.8. PubMed PMID: 2016-58440-008.

118. Cooper M, Harrell MB, Perry CL. Comparing young adults to older adults in e-cigarette perceptions and motivations for use: implications for health communication. Health Education Research. 2016;31(4):429-38. doi: 10.1093/her/cyw030. PubMed PMID: WOS:000383240400001.

119. Cooper M, Harrell MB, Perry CL. A Qualitative Approach to Understanding Real-World Electronic Cigarette Use: Implications for Measurement and Regulation. Preventing Chronic Disease. 2016;13. doi: 10.5888/pcd13.150502. PubMed PMID: WOS:000375199500007.

120. Cooper M, Loukas A, Harrell MB, Perry CL. College students' perceptions of risk and addictiveness of e-cigarettes and cigarettes. Journal of American College Health. 2017;65(2):103-11. doi: 10.1080/07448481.2016.1254638. PubMed PMID: 2017-03396-003.

121. Copeland AL, Peltier MR, Waldo K. Perceived risk and benefits of e-cigarette use among college students. Addictive Behaviors. 2017;71:31-7. doi: 10.1016/j.addbeh.2017.02.005. PubMed PMID: 2017-24399-007.

122. Copp SR, Collins JL, Dar R, Barrett SP. The effects of nicotine stimulus and response expectancies on male and female smokers' responses to nicotine-free electronic cigarettes. Addictive Behaviors. 2015;40:144-7. doi: 10.1016/j.addbeh.2014.09.013. PubMed PMID: WOS:000344824100024.

123. Cornuz J. [Prevention of smoking due to electronic cigarettes and new cigarette products: it is getting more complicated!]. Revue medicale suisse. 2015;11(478):1267-8. Epub 2015/07/28. PubMed PMID: 26211082.

124. Corsi DJ, Lippert AM. An examination of the shift in school-level clustering of US adolescent electronic cigarette use and its multilevel correlates, 2011–2013. Health & Place. 2016;38:30-8. doi: 10.1016/j.healthplace.2015.12.007. PubMed PMID: 2016-12810-005.

125. Costigan S, Lang B, Collard J. Risk assessment approach for e-cigarette flavours. Toxicology Letters. 2014;229:S127-S8. doi: 10.1016/j.toxlet.2014.06.455. PubMed PMID: WOS:000341134000402.

126. Cox S, Jakes S. Nicotine and e-cigarettes: Rethinking addiction in the context of reduced harm. International Journal of Drug Policy. 2017;44:84-5. doi: 10.1016/j.drugpo.2017.03.009. PubMed PMID: 2017-29613-009.

127. Cummins S, Leischow S, Bailey L, Bush T, Wassum K, Copeland L, et al. Knowledge and beliefs about electronic cigarettes among quitline cessation staff. Addictive Behaviors. 2016;60:78-83. doi: 10.1016/j.addbeh.2016.03.031. PubMed PMID: WOS:000377731500014.

128. Cuomo RE, Miner A, Mackey TK. Pricing and sales tax collection policies for e‐cigarette starter kits and disposable products sold online. Drug and Alcohol Review. 2016;35(1):110-4. doi: 10.1111/dar.12353. PubMed PMID: 2016-02863-008.

129. Czoli CD, Fong GT, Mays D, Hammond D. How do consumers perceive differences in risk across nicotine products? A review of relative risk perceptions across smokeless tobacco, e-cigarettes, nicotine replacement therapy and combustible cigarettes. Tobacco Control. 2017;26(E1):E49-E58. doi: 10.1136/tobaccocontrol-2016-053060. PubMed PMID: WOS:000397005400013.

130. Czoli CD, Goniewicz M, Islam T, Kotnowski K, Hammond D. Consumer preferences for electronic cigarettes: results from a discrete choice experiment. Tobacco Control. 2016;25(e1):e30-e6. doi: 10.1136/tobaccocontrol-2015-052422.

131. Czoli CD, Hammond D, Reid JL, Cole AG, Leatherdale ST. Use of Conventional and Alternative Tobacco and Nicotine Products Among a Sample of Canadian Youth. Journal of Adolescent Health. 2015;57(1):123-5. Epub 2015/05/06. doi: 10.1016/j.jadohealth.2015.03.006. PubMed PMID: 25937469.

132. Czoli CD, Hammond D, White CM. Electronic cigarettes in Canada: prevalence of use and perceptions among youth and young adults. Canadian Journal of Public Health. 2014;105(2):e97-e102. Epub 2014/06/03. PubMed PMID: 24886856.

133. Dai H, Hao J. Flavored Electronic Cigarette Use and Smoking Among Youth. Pediatrics. 2016;138(6). doi: 10.1542/peds.2016-2513. PubMed PMID: WOS:000388924800056.

134. Dai H, Hao J. Mining social media data for opinion polarities about electronic cigarettes. Tobacco Control. 2017;26(2):175-80. doi: 10.1136/tobaccocontrol-2015-052818. PubMed PMID: 2017-32076-009.

135. Dai HY, Hao JQ. Electronic cigarette and marijuana use among youth in the United States. Addictive Behaviors. 2017;66:48-54. doi: 10.1016/j.addbeh.2016.11.005. PubMed PMID: WOS:000392684300008.

136. Dautzenberg B, Berlin I, Tanguy M-L, Rieu N, Birkui P. Factors associated with experimentation of electronic cigarettes among Parisian teenagers in 2013. Tobacco Induced Diseases. 2015;13. doi: 10.1186/s12971-015-0065-4. PubMed PMID: WOS:000366450000001.

137. Davis B, Dang M, Kim J, Talbot P. Nicotine concentrations in electronic cigarette refill and do-it-yourself fluids. Nicotine & Tobacco Research. 2015;17(2):134-41. doi: 10.1093/ntr/ntu080. PubMed PMID: 2015-03033-003.

138. Dawkins L, Kimber C, Puwanesarasa Y, Soar K. First‐ versus second‐generation electronic cigarettes: Predictors of choice and effects on urge to smoke and withdrawal symptoms. Addiction. 2015;110(4):669-77. doi: 10.1111/add.12807. PubMed PMID: 2015-11906-012.

139. Dawkins L, Munafò M, Christoforou G, Olumegbon N, Soar K. The effects of e-cigarette visual appearance on craving and withdrawal symptoms in abstinent smokers. Psychology of Addictive Behaviors. 2016;30(1):101-5. doi: 10.1037/adb0000112. PubMed PMID: 2015-44635-001.

140. Dawkins L, Turner J, Hasna S, Soar K. The electronic-cigarette: Effects on desire to smoke, withdrawal symptoms and cognition. Addictive Behaviors. 2012;37(8):970-3. doi: 10.1016/j.addbeh.2012.03.004. PubMed PMID: 2012-10117-001.

141. Dawkins L, Turner J, Roberts A, Soar K. ‘Vaping’profiles and preferences: an online survey of electronic cigarette users. Addiction. 2013;108(6):1115-25.

142. de lacy E, Fletcher A, Hewitt G, Murphy S, Moore G. Cross-sectional study examining the prevalence, correlates and sequencing of electronic cigarette and tobacco use among 11-16-year olds in schools in Wales. BMJ open. 2017;7(2). doi: 10.1136/bmjopen-2016-012784. PubMed PMID: WOS:000397872400038.

143. Delnevo CD, Giovenco DP, Steinberg MB, Villanti AC, Pearson JL, Niaura RS, et al. Patterns of Electronic Cigarette Use Among Adults in the United States. Nicotine & Tobacco Research. 2016;18(5):715-9. doi: 10.1093/ntr/ntv237. PubMed PMID: WOS:000376350700029.

144. Delnevo CD, Villanti AC, Wackowski OA, Gundersen DA, Giovenco DP. The influence of menthol, e-cigarettes and other tobacco products on young adults’ self-reported changes in past year smoking. Tobacco Control. 2016;25(5):571-4. doi: 10.1136/tobaccocontrol-2015-052325. PubMed PMID: 2016-47762-003.

145. Demissie Z, Everett Jones S, Clayton HB, King BA. Adolescent Risk Behaviors and Use of Electronic Vapor Products and Cigarettes. Pediatrics. 2017;139(2). Epub 2017/01/25. doi: 10.1542/peds.2016-2921. PubMed PMID: 28115539.

146. Diamond WD. Consumer perceptions and intentions toward smoking cessation tools. Journal of Consumer Marketing. 2016;33(5):324-31. doi: 10.1108/jcm-06-2015-1452. PubMed PMID: 2016-37610-002.

147. Dinakar C, O'Connor GT. The Health Effects of Electronic Cigarettes. The New England journal of medicine. 2016;375(26):2608-9. Epub 2016/12/29. doi: 10.1056/NEJMc1613869. PubMed PMID: 28029910.

148. Dobbs PD, Hammig B, Henry LJ. E-cigarette use among US adolescents: Perceptions of relative addiction and harm. Health Education Journal. 2017;76(3):293-301. doi: 10.1177/0017896916671762. PubMed PMID: WOS:000408776700004.

149. Dockrell M, Morrison R, Bauld L, McNeill A. E-Cigarettes: Prevalence and Attitudes in Great Britain. Nicotine & Tobacco Research. 2013;15(10):1737-44. doi: 10.1093/ntr/ntt057. PubMed PMID: WOS:000326977100013.

150. Doran N, Brikmanis K. Expectancies for and use of e-cigarettes and hookah among young adult non-daily smokers. Addictive Behaviors. 2016;60:154-9. doi: 10.1016/j.addbeh.2016.04.008. PubMed PMID: 2016-26226-027.

151. Doran N, Brikmanis K, Petersen A, Delucchi K, Al-Delaimy WK, Luczak S, et al. Does e-cigarette use predict cigarette escalation? A longitudinal study of young adult non-daily smokers. Preventive Medicine. 2017;100:279-84. doi: 10.1016/j.ypmed.2017.03.023. PubMed PMID: WOS:000405677000040.

152. Douptcheva N, Gmel G, Studer J, Deline S, Etter J-F. Use of electronic cigarettes among young Swiss men. Journal of Epidemiology and Community Health. 2013;67(12):1075-6. doi: 10.1136/jech-2013-203152. PubMed PMID: 2013-42213-014.

153. Duderstadt KG. E-Cigarettes: Youth and Trends in Vaping. Journal of Pediatric Health Care. 2015;29(6):555-7. doi: 10.1016/j.pedhc.2015.07.008. PubMed PMID: WOS:000367511400011.

154. Duffy EK, Jenssen BP. Electronic Cigarettes: The New Face of Nicotine. Pediatrics. 2014;134(1):1-3. doi: 10.1542/peds.2013-3182. PubMed PMID: WOS:000338774800042.

155. Dunlop S, Lyons C, Dessaix A, Currow D. How are tobacco smokers using e-cigarettes? Patterns of use, reasons for use and places of purchase in New South Wales. Medical Journal of Australia. 2016;204(9). doi: 10.5694/mja15.01156. PubMed PMID: WOS:000410552500019.

156. Durmowicz EL. The impact of electronic cigarettes on the paediatric population. Tobacco Control. 2014;23:41-6. doi: 10.1136/tobaccocontrol-2013-051468. PubMed PMID: WOS:000334635400008.

157. Durmowicz EL, Rudy SF, Chen IL. Electronic cigarettes: analysis of FDA adverse experience reports in non-users. Tobacco Control. 2016;25(2):242. Epub 2015/04/25. doi: 10.1136/tobaccocontrol-2015-052235. PubMed PMID: 25908596; PubMed Central PMCID: PMCPMC4852954.

158. Dutra LM, Glantz SA. High international electronic cigarette use among never smoker adolescents. Journal of Adolescent Health. 2014;55(5):595-7. doi: 10.1016/j.jadohealth.2014.08.010. PubMed PMID: 2014-44637-001.

159. Dutra LM, Glantz SA. Electronic Cigarettes and Conventional Cigarette Use Among US Adolescents A Cross-sectional Study. JAMA Pediatrics. 2014;168(7):610-7. doi: 10.1001/jamapediatrics.2013.5488. PubMed PMID: WOS:000339492400009.

160. Dutra LM, Glantz SA. E-cigarettes and National Adolescent Cigarette Use: 2004-2014. Pediatrics. 2017;139(2). doi: 10.1542/peds.2016-2450. PubMed PMID: WOS:000393035100002.

161. Eastwood B, Dockrell MJ, Arnott D, Britton J, Cheeseman H, Jarvis MJ, et al. Electronic cigarette use in young people in Great Britain 2013-2014. Public Health. 2015;129(9):1150-6. doi: 10.1016/j.puhe.2015.07.009. PubMed PMID: WOS:000361631800003.

162. Eastwood B, East K, Brose LS, Dockrell MJ, Arnott D, Cheeseman H, et al. Electronic cigarette use in young people in Great Britain 2015-2016. Public Health. 2017;149:45-8. doi: 10.1016/j.puhe.2017.03.019. PubMed PMID: WOS:000407538900008.

163. Eggleston W, Nacca N, Stork CM, Marraffa JM. Pediatric death after unintentional exposure to liquid nicotine for an electronic cigarette. Clinical toxicology (Philadelphia, Pa). 2016;54(9):890-1. Epub 2016/07/08. doi: 10.1080/15563650.2016.1207081. PubMed PMID: 27383772.

164. Eissenberg T, Shihadeh A. Nicotine flux: a potentially important tool for regulating electronic cigarettes. Nicotine & Tobacco Research. 2015;17(2):165-7. Epub 2014/10/22. doi: 10.1093/ntr/ntu208. PubMed PMID: 25332456; PubMed Central PMCID: PMCPMC4838002.

165. EL-Hellani A, Salman R, El-Hage R, Talih S, Malek N, Baalbaki R, et al. Nicotine and carbonyl emissions from popular electronic cigarette products: correlation to liquid composition and design characteristics. Nicotine & Tobacco Research. 2018;20(2):215-23. doi: https://doi.org/10.1093/ntr/ntw280.

166. Elkalmi RM, Bhagavathul AS, Ya'u A, Al-Dubai SAR, Elsayed TM, Ahmad A, et al. Familiarity, perception, and reasons for electronic-cigarette experimentation among the general public in Malaysia: Preliminary insight. Journal of Pharmacy and Bioallied Sciences. 2016;8(3):240-7. doi: 10.4103/0975-7406.180768. PubMed PMID: WOS:000384438300011.

167. Etter J-F. Characteristics of users and usage of different types of electronic cigarettes: findings from an online survey. Addiction. 2016;111(4):724-33. doi: 10.1111/add.13240. PubMed PMID: WOS:000372907400025.

168. Etter J-F. A longitudinal study of cotinine in long-term daily users of e-cigarettes. Drug and Alcohol Dependence. 2016;160:218-21. doi: 10.1016/j.drugalcdep.2016.01.003. PubMed PMID: WOS:000371837200029.

169. Etter JF. Levels of saliva cotinine in electronic cigarette users. Addiction. 2014;109(5):825-9. Epub 2014/01/10. doi: 10.1111/add.12475. PubMed PMID: 24401004.

170. Etter JF. Explaining the effects of electronic cigarettes on craving for tobacco in recent quitters. Drug and Alcohol Dependence. 2015;148:102-8. Epub 2015/01/17. doi: 10.1016/j.drugalcdep.2014.12.030. PubMed PMID: 25592454.

171. Etter JF. Gateway effects and electronic cigarettes. Addiction. 2017. doi: 10.1111/add.13924. PubMed PMID: 2017-34368-001.

172. Etter JF, Bullen C. Electronic cigarette: Users profile, utilization, satisfaction and perceived efficacy. Addiction. 2011;106(11):2017-28. doi: 10.1111/j.1360-0443.2011.03505.x. PubMed PMID: 2011-23262-026.

173. Etter JF, Bullen C. A longitudinal study of electronic cigarette users. Addictive Behaviors. 2014;39(2):491-4. doi: 10.1016/j.addbeh.2013.10.028. PubMed PMID: WOS:000329479400021.

174. Etter JF, Eissenberg T. Dependence levels in users of electronic cigarettes, nicotine gums and tobacco cigarettes. Drug and Alcohol Dependence. 2015;147:68-75. Epub 2015/01/07. doi: 10.1016/j.drugalcdep.2014.12.007. PubMed PMID: 25561385; PubMed Central PMCID: PMCPMC4920051.

175. Fallin A, Miller A, Assef S, Ashford K. Perceptions of Electronic Cigarettes Among Medicaid-Eligible Pregnant and Postpartum Women. JOGNN. 2016;45(3):320-5. doi: 10.1016/j.jogn.2016.02.009. PubMed PMID: 116760209. Language: English. Entry Date: 20160715. Revision Date: 20160715. Publication Type: Article.

176. Farsalinos KE, Kistler KA, Gillman G, Voudris V. Evaluation of electronic cigarette liquids and aerosol for the presence of selected inhalation toxins. Nicotine & Tobacco Research. 2015;17(2):168-74. doi: 10.1093/ntr/ntu176. PubMed PMID: 2015-03033-009.

177. Farsalinos KE, Polosa R. Safety evaluation and risk assessment of electronic cigarettes as tobacco cigarette substitutes: a systematic review. Therapeutic advances in drug safety. 2014;5(2):67-86.

178. Farsalinos KE, Poulas K, Voudris V, Le Houezec J. Electronic cigarette use in the European Union: Analysis of a representative sample of 27460 Europeans from 28 countries. Addiction. 2016;111(11):2032-40. doi: 10.1111/add.13506. PubMed PMID: 2016-40813-001.

179. Farsalinos KE, Poulas K, Voudris V, Le Houezec J. E‐cigarette use in the European Union: Millions of smokers claim e‐cigarettes helped them quit. Addiction. 2017;112(3):545-6. doi: 10.1111/add.13722. PubMed PMID: 2017-05934-018.

180. Farsalinos KE, Romagna G, Voudris V. Factors associated with dual use of tobacco and electronic cigarettes: A case control study. International Journal of Drug Policy. 2015;26(6):595-600. doi: 10.1016/j.drugpo.2015.01.006. PubMed PMID: 2015-26026-011.

181. Fearon IM, Eldridge A, Gale N, Shepperd CJ, McEwan M, Camacho OM, et al. E-cigarette Nicotine Delivery: Data and Learnings from Pharmacokinetic Studies. American Journal of Health Behavior. 2017;41(1):16-32. doi: 10.5993/ajhb.41.1.2. PubMed PMID: WOS:000404465200002.

182. Feirman SP, Lock D, Cohen JE, Holtgrave DR, Li T. Flavored tobacco products in the United States: a systematic review assessing use and attitudes. Nicotine & Tobacco Research. 2015;18(5):739-49.

183. Ferkol T. E-Cigarettes - The New Smoking. Pediatric Pulmonology. 2017;52:S2-S3. doi: 10.1002/ppul.23727. PubMed PMID: WOS:000403792500003.

184. Filippidis FT, Laverty AA, Gerovasili V, Vardavas CI. Two-year trends and predictors of e-cigarette use in 27 European Union member states. Tobacco Control. 2017;26(1):98-104. doi: 10.1136/tobaccocontrol-2015-052771. PubMed PMID: WOS:000391439000022.

185. Fillon M. E-Cigarettes May Lead to Youth Tobacco Use: Evidence Mounts. Journal of the National Cancer Institute. 2016;108(2). Epub 2016/02/13. doi: 10.1093/jnci/djw016. PubMed PMID: 26864926.

186. Flora JW, Wilkinson CT, Sink KM, McKinney DL, Miller JH. Nicotine-related impurities in e-cigarette cartridges and refill e-liquids. Journal of Liquid Chromatography & Related Technologies. 2016;39(17-18):821-9. doi: 10.1080/10826076.2016.1266500. PubMed PMID: WOS:000395038100005.

187. Ford A, MacKintosh AM, Bauld L, Moodie C, Hastings G. Adolescents’ responses to the promotion and flavouring of e-cigarettes. International Journal of Public Health. 2016;61(2):215-24. doi: 10.1007/s00038-015-0769-5. PubMed PMID: 2015-56148-001.

188. Fotiou A, Kanavou E, Stavrou M, Richardson C, Kokkevi A. Prevalence and correlates of electronic cigarette use among adolescents in Greece: A preliminary cross-sectional analysis of nationwide survey data. Addictive Behaviors. 2015;51:88-92. doi: 10.1016/j.addbeh.2015.07.021. PubMed PMID: WOS:000361580900015.

189. Foulds J. Use of electronic cigarettes by adolescents. Journal of Adolescent Health. 2015;57(6):569-70. doi: 10.1016/j.jadohealth.2015.09.004. PubMed PMID: 2015-53973-001.

190. Foulds J, Veldheer S, Yingst J, Hrabovsky S, Wilson SJ, Nichols TT, et al. Development of a questionnaire for assessing dependence on electronic cigarettes among a large sample of ex-smoking e-cigarette users. Nicotine & Tobacco Research. 2015;17(2):186-92. doi: 10.1093/ntr/ntu204. PubMed PMID: 2015-03033-012.

191. Franck C, Budlovsky T, Windle SB, Filion KB, Eisenberg MJ. Electronic Cigarettes in North America. Circulation. 2014;129(19):1945-52. doi: 10.1161/circulationaha.113.006416. PubMed PMID: WOS:000335638500011.

192. Franks AM, Hawes WA, McCain KR, Payakachat N. Electronic cigarette use, knowledge, and perceptions among health professional students. Currents in Pharmacy Teaching and Learning. 2017;9(6):1003-9. doi: 10.1016/j.cptl.2017.07.023. PubMed PMID: WOS:000415595900010.

193. Fulmer EB, Neilands TB, Dube SR, Kuiper NM, Arrazola RA, Glantz SA. Protobacco Media Exposure and Youth Susceptibility to Smoking Cigarettes, Cigarette Experimentation, and Current Tobacco Use among US Youth. PLOS ONE. 2015;10(8):e0134734.

194. Furlow B. Are e-cigarettes and tobacco products losing their allure for US teenagers? Lancet Respiratory Medicine. 2017;5(8):612-. doi: 10.1016/s2213-2600(17)30267-9. PubMed PMID: WOS:000406042400013.

195. Gallart-Mateu D, Elbal L, Armenta S, de la Guardia M. Passive exposure to nicotine from e-cigarettes. Talanta. 2016;152:329-34. Epub 2016/03/20. doi: 10.1016/j.talanta.2016.02.014. PubMed PMID: 26992528.

196. Gallus S, Lugo A, Pacifici R, Pichini S, Colombo P, Garattini S, et al. E-Cigarette Awareness, Use, and Harm Perceptions in Italy: A National Representative Survey. Nicotine & Tobacco Research. 2014;16(12):1541-8. doi: 10.1093/ntr/ntu124. PubMed PMID: WOS:000345773300004.

197. Ganz O, Cantrell J, Moon-Howard J, Aidala A, Kirchner TR, Vallone D. Electronic cigarette advertising at the point-of-sale: a gap in tobacco control research. Tobacco control. 2015;24(e1):e110-e2.

198. General OotS. E-Cigarette Use Among Youth and Young Adults: A Report of the Surgeon General. Washington, DC: US Department of Health and Human Services, 2016.

199. Gilreath TD, Leventhal A, Barrington-Trimis JL, Unger JB, Cruz TB, Berhane K, et al. Patterns of alternative tobacco product use: Emergence of hookah and e-cigarettes as preferred products amongst youth. Journal of Adolescent Health. 2016;58(2):181-5. doi: 10.1016/j.jadohealth.2015.10.001. PubMed PMID: 2015-52716-001.

200. Giovenco DP, Casseus M, Duncan DT, Coups EJ, Lewis MJ, Delnevo CD. Association between electronic cigarette marketing near schools and e-cigarette use among youth. Journal of Adolescent Health. 2016;59(6):627-34. doi: 10.1016/j.jadohealth.2016.08.007. PubMed PMID: 2016-49005-001.

201. Giovenco DP, Delnevo CD. Prevalence of population smoking cessation by electronic cigarette use status in a national sample of recent smokers. Addictive Behaviors. 2018;76:129-34. doi: 10.1016/j.addbeh.2017.08.002. PubMed PMID: WOS:000412959900019.

202. Giovenco DP, Hammond D, Corey CG, Ambrose BK, Delnevo CD. E-Cigarette Market Trends in Traditional U.S. Retail Channels, 2012-2013. Nicotine & Tobacco Research. 2015;17(10):1279-83. doi: 10.1093/ntr/ntu282. PubMed PMID: 25542918.

203. Giovenco DP, Lewis MJ, Delnevo CD. Factors associated with e-cigarette use: A national population survey of current and former smokers. American Journal of Preventive Medicine. 2014;47(4):476-80. doi: 10.1016/j.amepre.2014.04.009. PubMed PMID: 2014-41295-018.

204. Glasser AM, Cobb CO, Teplitskaya L, Ganz O, Katz L, Rose SW, et al. Electronic nicotine delivery devices, and their impact on health and patterns of tobacco use: a systematic review protocol. BMJ open. 2015;5(4):e007688. Epub 2015/05/01. doi: 10.1136/bmjopen-2015-007688. PubMed PMID: 25926149; PubMed Central PMCID: PMCPMC4420972.

205. Glasser AM, Collins L, Pearson JL, Abudayyeh H, Niaura RS, Abrams DB, et al. Overview of Electronic Nicotine Delivery Systems: A Systematic Review. American Journal of Preventive Medicine. 2017;52(2):E33-E66. doi: 10.1016/j.amepre.2016.10.036. PubMed PMID: WOS:000396989700001.

206. Goh YH, Dujaili JA, Blebil AQ, Ahmed SI. Awareness and use of electronic cigarettes: Perceptions of health science programme students in Malaysia. Health Education Journal. 2017;76(8):1000-8. doi: 10.1177/0017896917732363. PubMed PMID: WOS:000416366600009.

207. Goldenson NI, Kirkpatrick MG, Barrington-Trimis JL, Pang RD, McBeth JF, Pentz MA, et al. Effects of sweet flavorings and nicotine on the appeal and sensory properties of e-cigarettes among young adult vapers: Application of a novel methodology. Drug & Alcohol Dependence. 2016;168:176-80. doi: 10.1016/j.drugalcdep.2016.09.014.

208. Goldenson NI, Leventhal AM, Stone MD, McConnell RS, Barrington-Trimis JL. Associations of Electronic Cigarette Nicotine Concentration With Subsequent Cigarette Smoking and Vaping Levels in Adolescents. JAMA Pediatrics. 2017;171(12):1192-9. doi: 10.1001/jamapediatrics.2017.3209. PubMed PMID: WOS:000416971000017.

209. Goney G. Electronic Cigarette (E-Cigarette) Using: Toxicological Aspects. Eurasian Journal of Pulmonology. 2017;19(1):1-7. doi: 10.5152/ejp.2016.49358. PubMed PMID: WOS:000399412200001.

210. Goniewicz ML, Gawron M, Nadolska J, Balwicki L, Sobczak A. Rise in Electronic Cigarette Use Among Adolescents in Poland. Journal of Adolescent Health. 2014;55(5):713-5. doi: 10.1016/j.jadohealth.2014.07.015. PubMed PMID: WOS:000344236300020.

211. Goniewicz ML, Gupta R, Lee YH, Reinhardt S, Kim S, Kim B, et al. Nicotine levels in electronic cigarette refill solutions: A comparative analysis of products from the US, Korea, and Poland. International Journal of Drug Policy. 2015;26(6):583-8. doi: 10.1016/j.drugpo.2015.01.020. PubMed PMID: 2015-26026-009.

212. Goniewicz ML, Kuma T, Gawron M, Knysak J, Kosmider L. Nicotine levels in electronic cigarettes. Nicotine & Tobacco Research. 2013;15(1):158-66. doi: 10.1093/ntr/nts103. PubMed PMID: 2013-09839-020.

213. Goniewicz ML, Leigh NJ, Gawron M, Nadolska J, Balwicki L, McGuire C, et al. Dual use of electronic and tobacco cigarettes among adolescents: A cross-sectional study in Poland. International Journal of Public Health. 2016;61(2):189-97. doi: 10.1007/s00038-015-0756-x. PubMed PMID: 2015-49989-001.

214. Goniewicz ML, Lingas EO, Hajek P. Patterns of electronic cigarette use and user beliefs about their safety and benefits: An Internet survey. Drug and Alcohol Review. 2013;32(2):133-40. doi: 10.1111/j.1465-3362.2012.00512.x. PubMed PMID: WOS:000315853300004.

215. Goniewicz ML, Zielinska-Danch W. Electronic Cigarette Use Among Teenagers and Young Adults in Poland. Pediatrics. 2012;130(4):E879-E85. doi: 10.1542/peds.2011-3448. PubMed PMID: WOS:000309412100012.

216. Gonzalez-Roz A, Secades-Villa R, Weidberg S. Evaluating nicotine dependence levels in e-cigarette users. Adicciones. 2017;29(2):136-8. doi: 10.20882/adicciones.905. PubMed PMID: WOS:000400804000009.

217. Gorukanti A, Delucchi K, Ling P, Fisher-Travis R, Halpern-Felsher B. Adolescents' attitudes towards e-cigarette ingredients, safety, addictive properties, social norms, and regulation. Preventive Medicine. 2017;94:65-71. doi: 10.1016/j.ypmed.2016.10.019. PubMed PMID: 2017-01131-012.

218. Gostin LO, Glasner AY. E-Cigarettes, Vaping, and Youth. JAMA. 2014;312(6):595-6. doi: 10.1001/jama.2014.7883. PubMed PMID: WOS:000340136400014.

219. Gowin M, Cheney MK, Wann TF. Knowledge and beliefs about e-cigarettes in straight-to-work young adults. Nicotine & Tobacco Research. 2017;19(2):208-14.

220. Grace RC, Kivell BM, Laugesen M. Gender differences in satisfaction ratings for nicotine electronic cigarettes by first-time users. Addictive Behaviors. 2015;50:140-3. doi: 10.1016/j.addbeh.2015.06.027. PubMed PMID: 2015-35054-025.

221. Grace RC, Kivell BM, Laugesen M. Estimating Cross-Price Elasticity of E-Cigarettes Using a Simulated Demand Procedure. Nicotine & Tobacco Research. 2015;17(5):592-8. doi: 10.1093/ntr/ntu268. PubMed PMID: WOS:000353904000011.

222. Grana R, Benowitz N, Glantz SA. E-Cigarettes A Scientific Review. Circulation. 2014;129(19):1972-86. doi: 10.1161/circulationaha.114.007667. PubMed PMID: WOS:000335638500014.

223. Grana RA. Electronic Cigarettes: A New Nicotine Gateway? Journal of Adolescent Health. 2013;52(2):135-6.

224. Gravely S, Fong GT, Cummings KM, Yan M, Quah ACK, Borland R, et al. Awareness, Trial, and Current Use of Electronic Cigarettes in 10 Countries: Findings from the ITC Project. International Journal of Environmental Research and Public Health. 2014;11(11):11691-704. doi: 10.3390/ijerph111111691. PubMed PMID: WOS:000345532000041.

225. Greenhill R, Dawkins L, Notley C, Finn MD, Turner JJD. Adolescent Awareness and Use of Electronic Cigarettes: A Review of Emerging Trends and Findings. Journal of Adolescent Health. 2016;59(6):612-9. doi: 10.1016/j.jadohealth.2016.08.005. PubMed PMID: WOS:000389534900002.

226. Gubner NR, Andrews KB, Mohammad-Zadeh A, Lisha NE, Guydish J. Electronic-cigarette use by individuals in treatment for substance abuse: A survey of 24 treatment centers in the United States. Addictive Behaviors. 2016;63:45-50. doi: 10.1016/j.addbeh.2016.06.025. PubMed PMID: 2016-41608-008.

227. Guillet S, Sicard S, Meynard J-B, Mayet A. Electronic cigarette: use and perceptions among French military nurses in 2013. Swiss Medical Weekly. 2015;145. doi: 10.4414/smw.2015.14137. PubMed PMID: WOS:000366561800003.

228. Guillory J, Kim A, Murphy J, Bradfield B, Nonnemaker J, Hsieh Y. Comparing Twitter and Online Panels for Survey Recruitment of E-Cigarette Users and Smokers. Journal of Medical Internet Research. 2016;18(11):e288. Epub 2016/11/17. doi: 10.2196/jmir.6326. PubMed PMID: 27847353; PubMed Central PMCID: PMCPMC5128722.

229. Haber LA, Ortiz GM. Clearing the air: inpatient providers' knowledge, perspectives, and experience with electronic cigarettes. Journal of hospital medicine. 2014;9(12):805-7. Epub 2014/10/30. doi: 10.1002/jhm.2279. PubMed PMID: 25352497.

230. Hajek P, Etter J-F, Benowitz N, Eissenberg T, McRobbie H. Electronic cigarettes: review of use, content, safety, effects on smokers and potential for harm and benefit. Addiction. 2014;109(11):1801-10. doi: 10.1111/add.12659. PubMed PMID: 103900964. Language: English. Entry Date: 20141013. Revision Date: 20151102. Publication Type: Journal Article.

231. Hajek P, Przulj D, Phillips A, Anderson R, McRobbie H. Nicotine delivery to users from cigarettes and from different types of e-cigarettes. Psychopharmacology. 2017;234(5):773-9. doi: 10.1007/s00213-016-4512-6. PubMed PMID: WOS:000394498200004.

232. Hall MG, Pepper JK, Morgan JC, Brewer NT. Social Interactions as a Source of Information about E-Cigarettes: A Study of US Adult Smokers. International Journal of Environmental Research and Public Health. 2016;13(8). doi: 10.3390/ijerph13080788. PubMed PMID: WOS:000382462900043.

233. Hall W, Gartner C. Should Australia reconsider its ban on the sale of electronic nicotine delivery systems? Lancet Respiratory Medicine. 2014;2(8):602-4. doi: 10.1016/s2213-2600(14)70155-9. PubMed PMID: WOS:000340337300007.

234. Hamilton HA, Ferrence R, Boak A, Schwartz R, Mann RE, O'Connor S, et al. Ever Use of Nicotine and Nonnicotine Electronic Cigarettes Among High School Students in Ontario, Canada. Nicotine & Tobacco Research. 2015;17(10):1212-8. doi: 10.1093/ntr/ntu234. PubMed PMID: WOS:000363175500005.

235. Hammal F, Finegan BA. Exploring attitudes of children 12–17 years of age toward electronic cigarettes. Journal of Community Health: The Publication for Health Promotion and Disease Prevention. 2016;41(5):962-8. doi: 10.1007/s10900-016-0178-6. PubMed PMID: 2016-43355-009.

236. Hammett E, Veldheer S, Yingst J, Hrabovsky S, Foulds J. Characteristics, use patterns and perceptions of electronic cigarette users who were never traditional cigarette smokers. Addictive Behaviors. 2017;65:92-7. doi: 10.1016/j.addbeh.2016.10.007. PubMed PMID: WOS:000390074000015.

237. Hammig B, Daniel-Dobbs P, Blunt-Vinti H. Electronic cigarette initiation among minority youth in the United States. American Journal of Drug and Alcohol Abuse. 2017;43(3):306-10. doi: 10.1080/00952990.2016.1203926. PubMed PMID: WOS:000400342400010.

238. Hammond D, Reid JL, Cole AG, Leatherdale ST. Electronic cigarette use and smoking initiation among youth: a longitudinal cohort study. Canadian Medical Association Journal. 2017;189(43):E1328-E36. doi: 10.1503/cmaj.161002. PubMed PMID: WOS:000414021600002.

239. Hanewinkel R, Isensee B. Risk factors for e-cigarette, conventional cigarette, and dual use in German adolescents: a cohort study. Preventive Medicine. 2015;74:59-62. Epub 2015/03/17. doi: 10.1016/j.ypmed.2015.03.006. PubMed PMID: 25770433.

240. Harrell M, Weaver S, Loukas A, Creamer M, Marti C, Jackson C, et al. Flavored e-cigarette use: Characterizing youth, young adult, and adult users. Preventive Medicine Reports. 2017;5:33-40. doi: https://doi.org/10.1016/j.pmedr.2016.11.001.

241. Harrell PT, Marquinez NS, Correa JB, Meltzer LR, Unrod M, Sutton SK, et al. Expectancies for Cigarettes, E-Cigarettes, and Nicotine Replacement Therapies Among E-Cigarette Users (aka Vapers). Nicotine & Tobacco Research. 2015;17(2):193-200. doi: 10.1093/ntr/ntu149. PubMed PMID: WOS:000350142300013.

242. Harrell PT, Simmons VN, Pineiro B, Correa JB, Menzie NS, Meltzer LR, et al. E-cigarettes and expectancies: why do some users keep smoking? Addiction. 2015;110(11):1833-43. doi: 10.1111/add.13043. PubMed PMID: WOS:000363329100024.

243. Harrington KF, Hull NC, Akindoju O, Kim Y, Hendricks PS, Kohler C, et al. Electronic Cigarette Awareness, Use History, and Expected Future Use Among Hospitalized Cigarette Smokers. Nicotine & Tobacco Research. 2014;16(11):1512-7. doi: 10.1093/ntr/ntu054. PubMed PMID: WOS:000344627800013.

244. Hartwell G, Thomas S, Egan M, Gilmore A, Petticrew M. E-cigarettes and equity: a systematic review of differences in awareness and use between sociodemographic groups. Tobacco Control. 2016;(0):1-7.

245. Hassan S, Anwar MU, Muthayya P, Jivan S. Burn injuries from exploding electronic cigarette batteries: An emerging public health hazard. Journal of plastic, reconstructive & aesthetic surgery : JPRAS. 2016;69(12):1716-8. Epub 2016/10/04. doi: 10.1016/j.bjps.2016.09.014. PubMed PMID: 27692636.

246. Havel CM, Benowitz NL, Jacob P, III, Helen GS. An Electronic Cigarette Vaping Machine for the Characterization of Aerosol Delivery and Composition. Nicotine & Tobacco Research. 2017;19(10):1224-31. doi: 10.1093/ntr/ntw147. PubMed PMID: WOS:000409185400014.

247. Helen GS, Dempsey DA, Havel CM, Jacob P, III, Benowitz NL. Impact of e-liquid flavors on nicotine intake and pharmacology of e-cigarettes. Drug and Alcohol Dependence. 2017;178:391-8. doi: 10.1016/j.drugalcdep.2017.05.042. PubMed PMID: WOS:000409152300055.

248. Henningfield JE, Zaatari GS. Electronic nicotine delivery systems: emerging science foundation for policy. Tobacco Control. 2010;19(2):89-90. doi: 10.1136/tc.2009.035279. PubMed PMID: 105201014. Language: English. Entry Date: 20100625. Revision Date: 20150711. Publication Type: Journal Article.

249. Hershberger AR, Karyadi KA, VanderVeen JD, Cyders MA. Beliefs about the direct comparison of e-cigarettes and cigarettes. Substance Use & Misuse. 2017;52(8):982-91. doi: 10.1080/10826084.2016.1268628. PubMed PMID: 2017-19675-003.

250. Hess CA, Antin TMJ, Annechino R, Hunt G. Perceptions of E-Cigarettes among Black Youth in California. International Journal of Environmental Research and Public Health. 2017;14(1). doi: 10.3390/ijerph14010060. PubMed PMID: WOS:000392578200060.

251. Hess CA, Olmedo P, Navas-Acien A, Goessler W, Cohen JE, Rule AM. E-cigarettes as a source of toxic and potentially carcinogenic metals. Environmental Research. 2017;152:221-5. Epub 2016/11/05. doi: 10.1016/j.envres.2016.09.026. PubMed PMID: 27810679; PubMed Central PMCID: PMCPMC5135636.

252. Higgins ST, Kurti AN, Redner R, White TJ, Gaalema DE, Roberts ME, et al. A literature review on prevalence of gender differences and intersections with other vulnerabilities to tobacco use in the United States, 2004–2014. Preventive Medicine. 2015;80:89-100. doi: 10.1016/j.ypmed.2015.06.009. PubMed PMID: 2015-45516-019.

253. Hildick-Smith GJ, Pesko MF, Shearer L, Hughes JM, Chang J, Loughlin GM, et al. A practitioner's guide to electronic cigarettes in the adolescent population. Journal of Adolescent Health. 2015;57(6):574-9. doi: 10.1016/j.jadohealth.2015.07.020. PubMed PMID: 2015-53973-003.

254. Hilton S, Weishaar H, Sweeting H, Trevisan F, Katikireddi SV. E-cigarettes, a safer alternative for teenagers? A UK focus group study of teenagers' views. BMJ open. 2016;6(11). doi: 10.1136/bmjopen-2016-013271. PubMed PMID: WOS:000391303400078.

255. Hinds JT, III, Loukas A, Chow S, Pasch KE, Harrell MB, Perry CL, et al. Using cognitive interviewing to better assess young adult e-cigarette use. Nicotine & Tobacco Research. 2016;18(10):1998-2005. doi: 10.1093/ntr/ntw096. PubMed PMID: 2016-46012-011.

256. Hines JZ, Fiala SC, Hedberg K. Electronic Cigarettes as an Introductory Tobacco Product Among Eighth and 11th Grade Tobacco Users - Oregon, 2015. MMWR Morb Mortal Wkly Rep. 2017;66(23):604-6. Epub 2017/06/16. doi: 10.15585/mmwr.mm6623a2. PubMed PMID: 28617772.

257. Hirano T, Tabuchi T, Nakahara R, Kunugita N, Mochizuki-Kobayashi Y. Electronic Cigarette Use and Smoking Abstinence in Japan: A Cross-Sectional Study of Quitting Methods. International Journal of Environmental Research and Public Health. 2017;14(2). Epub 2017/02/22. doi: 10.3390/ijerph14020202. PubMed PMID: 28218695; PubMed Central PMCID: PMCPMC5334756.

258. Hiratsuka VY, Avey JP, Trinidad SB, Beans JA, Robinson RF. Views on electronic cigarette use in tobacco screening and cessation in an Alaska Native healthcare setting. International journal of circumpolar health. 2015;74:27794. Epub 2015/10/22. doi: 10.3402/ijch.v74.27794. PubMed PMID: 26487575; PubMed Central PMCID: PMCPMC4612470.

259. Hiscock R, Bauld L, Arnott D, Dockrell M, Ross L, McEwen A. Views from the Coalface: What Do English Stop Smoking Service Personnel Think about E-Cigarettes? International Journal of Environmental Research and Public Health. 2015;12(12):16157-67. Epub 2015/12/26. doi: 10.3390/ijerph121215048. PubMed PMID: 26703638; PubMed Central PMCID: PMCPMC4690984.

260. Hitchman SC, Brose LS, Brown J, Robson D, McNeill A. Associations Between E-Cigarette Type, Frequency of Use, and Quitting Smoking: Findings From a Longitudinal Online Panel Survey in Great Britain. Nicotine & Tobacco Research. 2015;17(10):1187-94. doi: 10.1093/ntr/ntv078. PubMed PMID: WOS:000363175500002.

261. Hoffman AC, Salgado RV, Dresler C, Faller RW, Bartlett C. Flavour preferences in youth versus adults: A review. Tobacco Control. 2016;25(Suppl 2):32-9. doi: 10.1136/tobaccocontrol-2016-053192. PubMed PMID: 2016-61125-005.

262. Hoong GY, Dujaili JA, Blebil AQ, Ahmed SI. CHARACTERISTICS ASSOCIATED WITH AWARENESS AND USE OF ELECTRONIC NICOTINE DELIVERY SYSTEM (ENDS) AMONG STUDENTS ENROLLED IN HEALTH SCIENCE PROGRAMS. Value in Health. 2017;20(5):A201-A. PubMed PMID: WOS:000405448002355.

263. Hooper MW, Kolar SK. Racial/Ethnic Differences in Electronic Cigarette Use and Reasons for Use among Current and Former Smokers: Findings from a Community-Based Sample. International Journal of Environmental Research and Public Health. 2016;13(10). doi: 10.3390/ijerph13101009. PubMed PMID: WOS:000389570100076.

264. Hooper MW, Kolar SK. Racial/ethnic differences in electronic cigarette knowledge, social norms, and risk perceptions among current and former smokers. Addictive Behaviors. 2017;67:86-91. doi: 10.1016/j.addbeh.2016.12.013. PubMed PMID: WOS:000393004200015.

265. Huang J, Tauras J, Chaloupka FJ. The impact of price and tobacco control policies on the demand for electronic nicotine delivery systems. Tobacco Control. 2014;23:41-7. doi: 10.1136/tobaccocontrol-2013-051515. PubMed PMID: WOS:000339270700008.

266. Huang L-L, Baker HM, Meernik C, Ranney LM, Richardson A, Goldstein AO. Impact of non-menthol flavours in tobacco products on perceptions and use among youth, young adults and adults: a systematic review. Tobacco Control. 2017;26(6):709-19. doi: 10.1136/tobaccocontrol-2016-053196. PubMed PMID: WOS:000413529500026.

267. Huang L-L, Kowitt SD, Sutfin EL, Patel T, Ranney LM, Goldstein AO. Electronic cigarette use among high school students and its association with cigarette use and smoking cessation, North Carolina Youth Tobacco Surveys, 2011 and 2013. Preventing Chronic Disease. 2016;13. PubMed PMID: 2016-40193-001.

268. Hubbs AF, Cummings KJ, McKernan LT, Dankovic DA, Park RM, Kreiss K. Comment on Farsalinos et al., "Evaluation of Electronic Cigarette Liquids and Aerosol for the Presence of Selected Inhalation Toxins". Nicotine & Tobacco Research. 2015;17(10):1288-9. doi: 10.1093/ntr/ntu338. PubMed PMID: 25586777.

269. Huerta TR, Walker DM, Mullen D, Johnson TJ, Ford EW. Trends in e-cigarette awareness and perceived harmfulness in the U. S. American Journal of Preventive Medicine. 2017;52(3):339-46. doi: 10.1016/j.amepre.2016.10.017. PubMed PMID: 2017-08647-012.

270. Huh J, Leventhal AM. Intraindividual covariation between e-cigarette and combustible cigarette use in Korean American emerging adults. Psychology of Addictive Behaviors. 2016;30(2):246-51. doi: 10.1037/adb0000141. PubMed PMID: 2015-53537-001.

271. Hummel K, Hoving C, Nagelhout GE, de Vries H, van den Putte B, Candel MJJM, et al. Prevalence and reasons for use of electronic cigarettes among smokers: Findings from the International Tobacco Control (ITC) Netherlands Survey. International Journal of Drug Policy. 2015;26(6):601-8. doi: 10.1016/j.drugpo.2014.12.009. PubMed PMID: 2015-26026-012.

272. Hutzler C, Paschke M, Kruschinski S, Henkler F, Hahn J, Luch A. Chemical hazards present in liquids and vapors of electronic cigarettes. Archives of Toxicology. 2014;88(7):1295-308.

273. James SA. Electronic nicotine delivery systems and smoking reduction, substitution, and cessation. US: ProQuest Information & Learning; 2017.

274. Jawad M, Lee JT, Glantz S, Millett C. Price elasticity of demand of non-cigarette tobacco products: a systematic review and meta-analysis. Tobacco Control. 2018:tobaccocontrol-2017-054056.

275. Jeon C, Jung KJ, Kimm H, Lee S, Barrington-Trimis JL, McConnell R, et al. E-cigarettes, conventional cigarettes, and dual use in Korean adolescents and university students: Prevalence and risk factors. Drug and Alcohol Dependence. 2016;168:99-103. doi: 10.1016/j.drugalcdep.2016.08.636. PubMed PMID: 2016-52240-014.

276. Jiang N, Chen J, Wang M-P, McGhee SM, Kwong ACS, Lai VWY, et al. Electronic cigarette awareness and use among adults in Hong Kong. Addictive Behaviors. 2016;52:34-8. doi: 10.1016/j.addbeh.2015.08.008. PubMed PMID: WOS:000365377100006.

277. Jiang N, Wang MP, Ho SY, Leung LT, Lam TH. Electronic cigarette use among adolescents: a cross-sectional study in Hong Kong. BMC public health. 2016;16. doi: 10.1186/s12889-016-2719-4. PubMed PMID: WOS:000371856700001.

278. Jones CB, Hill ML, Pardini DA, Meier MH. Prevalence and correlates of vaping cannabis in a sample of young adults. Psychology of Addictive Behaviors. 2016;30(8):915-21. doi: 10.1037/adb0000217 10.1037/adb0000217.supp (Supplemental). PubMed PMID: 2016-44358-001.

279. Jones DM, Majeed BA, Weaver SR, Sterling K, Pechacek TF, Eriksen MP. Prevalence and Factors Associated with Smokeless Tobacco Use, 2014-2016. American Journal of Health Behavior. 2017;41(5):608-17. doi: 10.5993/ajhb.41.5.10. PubMed PMID: WOS:000407973600010.

280. Jorenby DE, Smith SS, Fiore MC, Baker TB. Nicotine levels, withdrawal symptoms, and smoking reduction success in real world use: A comparison of cigarette smokers and dual users of both cigarettes and E-cigarettes. Drug and Alcohol Dependence. 2017;170:93-101. doi: 10.1016/j.drugalcdep.2016.10.041. PubMed PMID: 2016-62577-014.

281. Kadimpati S, Nolan M, Warner DO. Attitudes, beliefs, and practices regarding electronic nicotine delivery systems in patients scheduled for elective surgery. Mayo Clinic Proceedings. 2015;90(1):71-6. doi: 10.1016/j.mayocp.2014.11.005. PubMed PMID: 103874930. Language: English. Entry Date: 20150410. Revision Date: 20150710. Publication Type: Journal Article.

282. Kaleta D, Wojtysiak P, Polanska K. Use of electronic cigarettes among secondary and high school students from a socially disadvantaged rural area in Poland. BMC public health. 2016;16. doi: 10.1186/s12889-016-3417-y. PubMed PMID: WOS:000381004800002.

283. Kalkhoran S, Glantz SA. E-cigarettes and smoking cessation in real-world and clinical settings: a systematic review and meta-analysis. Lancet Respiratory Medicine. 2016;4(2):116-28. doi: 10.1016/s2213-2600(15)00521-4. PubMed PMID: WOS:000370113400019.

284. Kalkhoran S, Grana RA, Neilands TB, Ling PM. Dual Use of Smokeless Tobacco or E-cigarettes with Cigarettes and Cessation. American Journal of Health Behavior. 2015;39(2):276-83. doi: 10.5993/ajhb.39.2.14. PubMed PMID: WOS:000352542300014.

285. Kalkhoran S, Padilla JL, Neilands TB, Ling PM. Multiple tobacco product use among young adult bar patrons in New Mexico. Preventive Medicine. 2016;83:16-21. Epub 2015/12/17. doi: 10.1016/j.ypmed.2015.11.024. PubMed PMID: 26666395; PubMed Central PMCID: PMCPMC5433518.

286. Kalousova L. E-cigarettes: a harm-reduction strategy for socioeconomically disadvantaged smokers? Lancet Respiratory Medicine. 2015;3(8):598-600. Epub 2015/06/28. doi: 10.1016/s2213-2600(15)00239-8. PubMed PMID: 26116291.

287. Kamat AD, Van Dyke AL. Use of Electronic Nicotine Delivery Systems Among Adolescents: Status of the Evidence and Public Health Recommendations. Pediatric annals. 2017;46(2):e69-e77. Epub 2017/02/14. doi: 10.3928/19382359-20170111-01. PubMed PMID: 28192582.

288. Kanchustambham V, Saladi S, Rodrigues J, Fernandes H, Patolia S, Santosh S. The knowledge, concerns and healthcare practices among physicians regarding electronic cigarettes. Journal of Community Hospital Internal Medicine Perspectives. 2017;7(3):144-50. doi: 10.1080/20009666.2017.1343076. PubMed PMID: WOS:000405898000002.

289. Kaplan A. Rebuttal: Can electronic cigarettes assist patients with smoking cessation? Yes. Canadian Family Physician. 2015;61(6):e255, e7. Epub 2015/06/14. PubMed PMID: 26071163; PubMed Central PMCID: PMCPMC4463904.

290. Kaufmann N, Currie D. The Scottish adolescent e-cigarette user: profiling from the Scottish Schools Adolescent Lifestyle and Substance Use Survey (SALSUS). Public Health. 2017;147:69-71. doi: 10.1016/j.puhe.2017.02.004. PubMed PMID: WOS:000405833500012.

291. Kenne DR, Mix D, Banks M, Fischbein R. Electronic cigarette initiation and correlates of use among never, former, and current tobacco cigarette smoking college students. Journal of Substance Use. 2016;21(5):491-4. doi: 10.3109/14659891.2015.1068387. PubMed PMID: WOS:000377931500010.

292. Khlystov A, Samburova V. Flavoring Compounds Dominate Toxic Aldehyde Production during E-Cigarette Vaping. Environmental Science & Technology. 2016;50(23):13080-5. doi: 10.1021/acs.est.6b05145. PubMed PMID: WOS:000389557100062.

293. Khoury M, Manlhiot C, Fan C-PS, Gibson D, Stearne K, Chahal N, et al. Reported electronic cigarette use among adolescents in the Niagara region of Ontario. Canadian Medical Association Journal. 2016;188(11):794-800. doi: 10.1503/cmaj.151169. PubMed PMID: 2016-49609-003.

294. Kilibarda B, Mravcik V, Martens MS. E-cigarette use among serbian adults: Prevalence and user characteristics. International Journal of Public Health. 2016. doi: 10.1007/s00038-016-0787-y. PubMed PMID: 2016-05497-001.

295. Kim AE, Arnold KY, Makarenko O. E-cigarette advertising expenditures in the US, 2011–2012. American Journal of Preventive Medicine. 2014;46(4):409-12.

296. Kim AE, Hopper T, Simpson S, Nonnemaker J, Lieberman AJ, Hansen H, et al. Using Twitter Data to Gain Insights into E-cigarette Marketing and Locations of Use: An Infoveillance Study. Journal of Medical Internet Research. 2015;17(11):e251. Epub 2015/11/08. doi: 10.2196/jmir.4466. PubMed PMID: 26545927; PubMed Central PMCID: PMCPMC4642798.

297. Kim AE, Lee YO, Shafer P, Nonnemaker J, Makarenko O. Adult smokers' receptivity to a television advert for electronic nicotine delivery systems. Tobacco Control. 2015;24(2):132-5. Epub 2013/10/05. doi: 10.1136/tobaccocontrol-2013-051130. PubMed PMID: 24092599.

298. Kim H, Davis AH, Dohack JL, Clark PI. E-cigarettes use behavior and experience of adults: Qualitative research findings to inform e-cigarette use measure development. Nicotine & Tobacco Research. 2017;19(2):190-6. doi: 10.1093/ntr/ntw175. PubMed PMID: 2017-30934-008.

299. Kim H, Lim J, Buehler SS, Brinkman MC, Johnson NM, Wilson L, et al. Role of sweet and other flavours in liking and disliking of electronic cigarettes. Tobacco Control. 2016;25(Suppl 2):55-61. doi: 10.1136/tobaccocontrol-2016-053221. PubMed PMID: 2016-61125-008.

300. Kim JW, Baum CR. Liquid Nicotine Toxicity. Pediatric Emergency Care. 2015;31(7):517-21. doi: 10.1097/pec.0000000000000486. PubMed PMID: WOS:000357943600012.

301. Kim KH, Kabir E, Jahan SA. Review of electronic cigarettes as tobacco cigarette substitutes: Their potential human health impact. Journal of environmental science and health Part C, Environmental carcinogenesis & ecotoxicology reviews. 2016;34(4):262-75. Epub 2016/09/17. doi: 10.1080/10590501.2016.1236604. PubMed PMID: 27635466.

302. King AC, Smith LJ, Fridberg DJ, Matthews AK, McNamara PJ, Cao D. Exposure to Electronic Nicotine Delivery Systems (ENDS) Visual Imagery Increases Smoking Urge and Desire. Psychology of Addictive Behaviors. 2016;30(1):106-12. doi: 10.1037/adb0000123. PubMed PMID: WOS:000370570100012.

303. King AC, Smith LJ, McNamara PJ, Matthews AK, Fridberg DJ. Passive exposure to electronic cigarette (e-cigarette) use increases desire for combustible and e-cigarettes in young adult smokers. Tobacco Control. 2015;24(5):501-4. doi: 10.1136/tobaccocontrol-2014-051563. PubMed PMID: 2015-46371-014.

304. King BA, Alam S, Promoff G, Arrazola R, Dube SR. Awareness and ever-use of electronic cigarettes among U.S. adults, 2010–2011. Nicotine & Tobacco Research. 2013;15(9):1623-7. doi: 10.1093/ntr/ntt013. PubMed PMID: 2013-29320-019.

305. King BA, Patel R, Nguyen KH, Dube SR. Trends in awareness and use of electronic cigarettes among US adults, 2010–2013. Nicotine & Tobacco Research. 2015;17(2):219-27. doi: 10.1093/ntr/ntu191. PubMed PMID: 2015-03033-017.

306. Kinnunen JM. Electronic cigarettes and adolescents. Nordic Studies on Alcohol and Drugs. 2016;33(3):243-5. doi: 10.1515/nsad-2016-0019. PubMed PMID: 2016-45910-004.

307. Kinnunen JM, Minkkinen JLK, Ollila H, Rimpela A. Follow-up of adolescent electronic cigarette use in Finland. European Journal of Public Health. 2017;27. PubMed PMID: WOS:000414389801083.

308. Kinnunen JM, Ollila H, El-Amin SE-T, Pere LA, Lindfors PL, Rimpelä AH. Awareness and determinants of electronic cigarette use among Finnish adolescents in 2013: A population-based study. Tobacco Control. 2015;24(e4):e264-e70. doi: 10.1136/tobaccocontrol-2013-051512. PubMed PMID: 2016-60341-005.

309. Kinnunen JM, Ollila H, Lindfors PL, Rimpelae AH. Changes in Electronic Cigarette Use from 2013 to 2015 and Reasons for Use among Finnish Adolescents. International Journal of Environmental Research and Public Health. 2016;13(11). doi: 10.3390/ijerph13111114. PubMed PMID: WOS:000389571300073.

310. Kinouani S, Pereira E, Tzourio C. Electronic Cigarette Use in Students and Its Relation with Tobacco-Smoking: A Cross-Sectional Analysis of the i-Share Study. International Journal of Environmental Research and Public Health. 2017;14(11). doi: 10.3390/ijerph14111345. PubMed PMID: WOS:000416545200061.

311. Kistler CE, Crutchfield TM, Sutfin EL, Ranney LM, Berman ML, Zarkin GA, et al. Consumers' Preferences for Electronic Nicotine Delivery System Product Features: A Structured Content Analysis. International Journal of Environmental Research and Public Health. 2017;14(6). Epub 2017/06/08. doi: 10.3390/ijerph14060613. PubMed PMID: 28590444; PubMed Central PMCID: PMCPMC5486299.

312. Klager S, Vallarino J, MacNaughton P, Christian DC, Lu Q, Allen JG. Flavoring Chemicals and Aldehydes in E-Cigarette Emissions. Environmental Science & Technology. 2017;51(18):10806-13. doi: 10.1021/acs.est.7b02205. PubMed PMID: WOS:000411549800059.

313. Kong G, Cavallo DA, Camenga DR, Morean ME, Krishnan-Sarin S. Preference for gain- or loss-framed electronic cigarette prevention messages. Addictive Behaviors. 2016;62:108-13. doi: 10.1016/j.addbeh.2016.06.015. PubMed PMID: 116862596. Language: English. Entry Date: In Process. Revision Date: 20171105. Publication Type: journal article. Journal Subset: Biomedical.

314. Kong G, Idrisov B, Galimov A, Masagutov R, Sussman S. Electronic cigarette use among adolescents in the Russian Federation. Substance Use & Misuse. 2017;52(3):332-9. doi: 10.1080/10826084.2016.1225766. PubMed PMID: 2017-02628-007.

315. Kong G, Morean ME, Cavallo DA, Camenga DR, Krishnan-Sarin S. Reasons for Electronic Cigarette Experimentation and Discontinuation Among Adolescents and Young Adults. Nicotine & Tobacco Research. 2015;17(7):847-54. doi: 10.1093/ntr/ntu257. PubMed PMID: WOS:000357888500012.

316. Kornfield R, Huang J, Vera L, Emery SL. Rapidly increasing promotional expenditures for e-cigarettes. Tobacco Control. 2015;24(2):110-1.

317. Korry E. San Francisco Seeks To Ban Sale Of Menthol Cigarettes, Flavored Tobacco Products2017 04/19/2017 [cited 2017 04/19/2017]. Available from: http://californiahealthline.org/news/sf-seeks-to-ban-sale-of-menthol-cigarettes-and-flavored-tobacco-products/.

318. Kosmider L, Sobczak A, Fik M, Knysak J, Zaciera M, Kurek J, et al. Carbonyl compounds in electronic cigarette vapors: effects of nicotine solvent and battery output voltage. Nicotine & Tobacco Research. 2014;16(10):1319-26. Epub 2014/05/17. doi: 10.1093/ntr/ntu078. PubMed PMID: 24832759; PubMed Central PMCID: PMCPMC4838028.

319. Kosmider L, Sobczak A, Prokopowicz A, Kurek J, Zaciera M, Knysak J, et al. Cherry-flavoured electronic cigarettes expose users to the inhalation irritant, benzaldehyde. Thorax. 2016;71(4):376-7. doi: 10.1136/thoraxjnl-2015-207895. PubMed PMID: WOS:000372285700013.

320. Kotecha S, Jawad M, Iliffe S. Knowledge, attitudes and beliefs towards waterpipe tobacco smoking and electronic shisha (e-shisha) among young adults in London: a qualitative analysis. Primary Health Care Research & Development. 2016;17(2):166-74. doi: 10.1017/s1463423615000237. PubMed PMID: 25864374.

321. Kralikova E, Novak J, West O, Kmetova A, Hajek P. Do e-cigarettes have the potential to compete with conventional cigarettes?: a survey of conventional cigarette smokers' experiences with e-cigarettes. Chest. 2013;144(5):1609-14. doi: 10.1378/chest.12-2842. PubMed PMID: 107917821. Language: English. Entry Date: 20140124. Revision Date: 20170928. Publication Type: journal article.

322. Krishnan-Sarin S, Green BG, Kong G, Cavallo DA, Jatlow P, Gueorguieva R, et al. Studying the interactive effects of menthol and nicotine among youth: An examination using e-cigarettes. Drug and Alcohol Dependence. 2017;180:193-9. doi: 10.1016/j.drugalcdep.2017.07.044. PubMed PMID: WOS:000414816600026.

323. Krishnan-Sarin S, Morean M, Kong G, Bold KW, Camenga DR, Cavallo DA, et al. E-Cigarettes and "Dripping" Among High-School Youth. Pediatrics. 2017;139(3). Epub 2017/02/09. doi: 10.1542/peds.2016-3224. PubMed PMID: 28167512; PubMed Central PMCID: PMCPMC5330405.

324. Krishnan-Sarin S, Morean ME, Camenga DR, Cavallo DA, Kong G. E-cigarette use among high school and middle school adolescents in Connecticut. Nicotine & Tobacco Research. 2015;17(7):810-8. doi: 10.1093/ntr/ntu243. PubMed PMID: 2015-26024-007.

325. Kristjansson AL, Mann MJ, Smith ML, Sigfusdottir ID. Social profile of middle school-aged adolescents who use electronic cigarettes: Implications for primary prevention. Prevention Science. 2017. doi: 10.1007/s11121-017-0825-x. PubMed PMID: 2017-31872-001.

326. Kruse GR, Kalkhoran S, Rigotti NA. Use of Electronic Cigarettes Among US Adults With Medical Comorbidities. American Journal of Preventive Medicine. 2017;52(6):798-804. doi: 10.1016/j.amepre.2016.12.004. PubMed PMID: WOS:000405284100016.

327. Kusiak A, Wojtaszek-Slominska A, Chomyszyn-Gajewska M, Pietruska M, Maj A, Szkarlat B, et al. Analysis of electronic cigarette use among Polish dental students. Dental and Medical Problems. 2017;54(3):263-6. doi: 10.17219/dmp/76708. PubMed PMID: WOS:000417388900008.

328. Lam C, West A. Are electronic nicotine delivery systems an effective smoking cessation tool? Canadian Journal of Respiratory Therapy. 2015;51(4):93-8. PubMed PMID: 110709577. Language: English. Entry Date: 20151109. Revision Date: 20151130. Publication Type: Article.

329. Lanza ST, Russell MA, Braymiller JL. Emergence of electronic cigarette use in US adolescents and the link to traditional cigarette use. Addictive Behaviors. 2017;67:38-43. doi: 10.1016/j.addbeh.2016.12.003. PubMed PMID: WOS:000393004200007.

330. Larson E, Pearlman DN. Use of emerging tobacco products among adolescents who do not smoke conventional cigarettes. Rhode Island medical journal (2013). 2016;99(6):45-7. Epub 2016/06/02. PubMed PMID: 27247974.

331. Laverty AA, Vardavas CI, Filippidis FT. Design and marketing features influencing choice of e-cigarettes and tobacco in the EU. European Journal of Public Health. 2016;26(5):838-41. doi: 10.1093/eurpub/ckw109. PubMed PMID: 2017-26386-012.

332. Lechner WV, Meier E, Wiener JL, Grant DM, Gilmore J, Judah MR, et al. The comparative efficacy of first- versus second-generation electronic cigarettes in reducing symptoms of nicotine withdrawal. Addiction. 2015;110(5):862-7. Epub 2015/02/03. doi: 10.1111/add.12870. PubMed PMID: 25639148.

333. Lee AH, Stater BJ, Close L, Rahmati R. Are e-cigarettes effective in smoking cessation? The Laryngoscope. 2015;125(4):785-7. Epub 2014/11/13. doi: 10.1002/lary.24954. PubMed PMID: 25387447.

334. Lee H-Y, Lin H-C, Seo D-C, Lohrmann DK. Determinants associated with E-cigarette adoption and use intention among college students. Addictive Behaviors. 2017;65:102-10. doi: 10.1016/j.addbeh.2016.10.023. PubMed PMID: WOS:000390074000017.

335. Lee H-Y, Lin H-C, Seo D-C, Lohrmann DK. The effect of e-cigarette warning labels on college students' perception of e-cigarettes and intention to use e-cigarettes. Addictive Behaviors. 2018;76:106-12. doi: 10.1016/j.addbeh.2017.07.033. PubMed PMID: WOS:000412959900016.

336. Lee JA, Kim SH, Cho H-J. Electronic cigarette use among Korean adults. International Journal of Public Health. 2016;61(2):151-7. doi: 10.1007/s00038-015-0763-y. PubMed PMID: 2015-52204-001.

337. Lee S, Grana RA, Glantz SA. Electronic Cigarette Use Among Korean Adolescents: A Cross-Sectional Study of Market Penetration, Dual Use, and Relationship to Quit Attempts and Former Smoking. Journal of Adolescent Health. 2014;54(6):684-90. doi: 10.1016/j.jadohealth.2013.11.003. PubMed PMID: WOS:000336517400009.

338. Lee YO, Hebert CJ, Nonnemaker JM, Kim AE. Multiple tobacco product use among adults in the United States: cigarettes, cigars, electronic cigarettes, hookah, smokeless tobacco, and snus. Preventive Medicine. 2014;62:14-9. doi: 10.1016/j.ypmed.2014.01.014. PubMed PMID: 109666350. Language: English. Entry Date: 20150923. Revision Date: 20150924. Publication Type: journal article. Journal Subset: Biomedical.

339. Lehmann K, Kuhn S, Reimer J. Consumer's Point of View of Individual and Social Aspects of E-Cigarette use: A Qualitative Approach. Suchttherapie. 2017;18(3):126-33. doi: 10.1055/s-0043-113858. PubMed PMID: WOS:000407379600010.

340. Lehmann K, Kuhn S, Reimer J. Electronic Cigarettes in Germany: Patterns of Use and Perceived Health Improvement. European Addiction Research. 2017;23(3):136-47. doi: 10.1159/000475986. PubMed PMID: WOS:000405751500004.

341. Leigh NJ, Lawton RI, Hershberger PA, Goniewicz ML. Flavourings significantly affect inhalation toxicity of aerosol generated from electronic nicotine delivery systems (ENDS). Tobacco Control. 2016;25(Suppl 2):ii81-ii7.

342. Levy DT, Cummings KM, Villanti AC, Niaura R, Abrams DB, Fong GT, et al. A framework for evaluating the public health impact of e-cigarettes and other vaporized nicotine products. Addiction. 2017;112(1):8-17. doi: 10.1111/add.13394. PubMed PMID: WOS:000393904300003.

343. Levy DT, Yuan Z, Li Y. The Prevalence and Characteristics of E-Cigarette Users in the US. International Journal of Environmental Research and Public Health. 2017;14(10). doi: 10.3390/ijerph14101200. PubMed PMID: WOS:000414763200109.

344. Li J, Newcombe R, Newcombe R, Walton D. Susceptibility to e-cigarette use among never-users: findings from a survey of New Zealand adult smokers and ex-smokers. The New Zealand medical journal. 2015;128(1421):65-8. Epub 2015/09/16. PubMed PMID: 26370759.

345. Li J, Newcombe R, Walton D. The use of, and attitudes towards, electronic cigarettes and self‐reported exposure to advertising and the product in general. Australian and New Zealand journal of public health. 2014;38(6):524-8.

346. Li J, Newcombe R, Walton D. The prevalence, correlates and reasons for using electronic cigarettes among New Zealand adults. Addictive Behaviors. 2015;45:245-51. doi: 10.1016/j.addbeh.2015.02.006. PubMed PMID: 2015-13545-044.

347. Li J, Newcombe R, Walton D. Contextual information around the first use of electronic cigarettes among New Zealand smokers and recent quitters. Nicotine & Tobacco Research. 2016;18(5):737-8. doi: 10.1093/ntr/ntv143. PubMed PMID: 2016-40277-033.

348. Liang Y, Zheng X, Zeng DD, Zhou X. Impact of Flavor on Electronic Cigarette Marketing in Social Media. In: Zheng X, Zeng DD, Chen H, Leischow SJ, editors. Smart Health, Icsh 2015. Lecture Notes in Computer Science. 95452016. p. 278-83.

349. Lindblom EN. Effectively regulating e-cigarettes and their advertising-and the first amendment. Food and Drug Law Journal. 2015;70:55.

350. Liozidou AI, Dimou N, Lioupa A, Behrakis P. Experimentation With Cigarettes and e-Cigarettes Among Greek Adolescents. Chest. 2016;149(4):594A-A. doi: 10.1016/j.chest.2016.02.620. PubMed PMID: WOS:000400116000593.

351. Lippert AM. Do Adolescent Smokers Use E-Cigarettes to Help Them Quit? The Sociodemographic Correlates and Cessation Motivations of US Adolescent E-Cigarette Use. American Journal of Health Promotion. 2015;29(6):374-9. doi: 10.4278/ajhp.131120-QUAN-595. PubMed PMID: WOS:000357359500006.

352. Lisko JG, Lee GE, Kimbrell JB, Rybak ME, Valentin-Blasini L, Watson CH. Caffeine concentrations in coffee, tea, chocolate, and energy drink flavored e-liquids. Nicotine & Tobacco Research. 2017;19(4):484-92. PubMed PMID: 2017-30597-012.

353. Litt MD, Duffy V, Oncken C. Cigarette smoking and electronic cigarette vaping patterns as a function of e-cigarette flavourings. Tobacco Control. 2016;(0):1-6. doi: 10.1136/tobaccocontrol-2016-053223.

354. Littlefield AK, Gottlieb JC, Cohen LM, Trotter DRM. Electronic Cigarette Use Among College Students: Links to Gender, Race/Ethnicity, Smoking, and Heavy Drinking. Journal of American College Health. 2015;63(8):523-9. doi: 10.1080/07448481.2015.1043130. PubMed PMID: WOS:000364769900002.

355. Loomis BR, Rogers T, King BA, Dench DL, Gammon DG, Fulmer EB, et al. National and State-Specific Sales and Prices for Electronic Cigarettes-U.S., 2012-2013. American Journal of Preventive Medicine. 2016;50(1):18-29. Epub 2015/07/15. doi: 10.1016/j.amepre.2015.05.003. PubMed PMID: 26163173; PubMed Central PMCID: PMCPMC4691554.

356. Lotrean LM. Use of electronic cigarettes among Romanian university students: a cross-sectional study. BMC public health. 2015;15:358. Epub 2015/04/19. doi: 10.1186/s12889-015-1713-6. PubMed PMID: 25888354; PubMed Central PMCID: PMCPMC4414446.

357. Loukas A, Marti CN, Cooper M, Pasch KE, Perry CL. Exclusive e-cigarette use predicts cigarette initiation among college students. Addictive Behaviors. 2018;76:343-7. doi: 10.1016/j.addbeh.2017.08.023. PubMed PMID: WOS:000412959900051.

358. Lozano P, Barrientos-Gutierrez I, Arillo-Santillan E, Morello P, Mejia R, Sargent JD, et al. A longitudinal study of electronic cigarette use and onset of conventional cigarette smoking and marijuana use among Mexican adolescents. Drug and Alcohol Dependence. 2017;180:427-30. doi: 10.1016/j.drugalcdep.2017.09.001. PubMed PMID: WOS:000414816600058.

359. Lund KE. The role of e-cigarettes in the tobacco endgame. Nordic Studies on Alcohol and Drugs. 2016;33(3):241-2. doi: 10.1515/nsad-2016-0018. PubMed PMID: WOS:000379145900003.

360. Majeed BA, Weaver SR, Gregory KR, Whitney CF, Slovic P, Pechacek TF, et al. Changing Perceptions of Harm of E-Cigarettes Among US Adults, 2012-2015. American Journal of Preventive Medicine. 2017;52(3):331-8. doi: 10.1016/j.amepre.2016.08.039. PubMed PMID: WOS:000400434200013.

361. Malas M, van der Tempel J, Schwartz R, Minichiello A, Lightfoot C, Noormohamed A, et al. Electronic Cigarettes for Smoking Cessation: A Systematic Review. Nicotine & Tobacco Research. 2016;18(10):1926-36. doi: 10.1093/ntr/ntw119. PubMed PMID: WOS:000386201100002.

362. Maloney EK, Cappella JN. Does vaping in e-cigarette advertisements affect tobacco smoking urge, intentions, and perceptions in daily, intermittent, and former smokers? Health communication. 2016;31(1):129-38.

363. Mantey DS, Cooper MR, Clendennen SL, Pasch KE, Perry CL. E-Cigarette Marketing Exposure Is Associated With E-Cigarette Use Among US Youth. Journal of Adolescent Health. 2016;58(6):686-90.

364. Margolis KA, Nguyen AB, Slavit WI, King BA. E-cigarette curiosity among U.S. middle and high school students: Findings from the 2014 national youth tobacco survey. Preventive Medicine. 2016;89:1-6. Epub 2016/05/08. doi: 10.1016/j.ypmed.2016.05.001. PubMed PMID: 27155440; PubMed Central PMCID: PMCPMC4969114.

365. Mark KS, Farquhar B, Chisolm MS, Coleman-Cowger VH, Terplan M. Knowledge, Attitudes, and Practice of Electronic Cigarette Use Among Pregnant Women. Journal of Addiction Medicine. 2015;9(4):266-72. doi: 10.1097/adm.0000000000000128. PubMed PMID: WOS:000369710500004.

366. Martinez DJ. What may be associated with young adult e-cigarette use? Application of the Integrated Behavior Model and affect heuristic to examine key correlates. US: ProQuest Information & Learning; 2018.

367. Martinez-Sanchez JM, Ballbe M, Fu M, Carlos Martin-Sanchez J, Salto E, Gottlieb M, et al. Electronic cigarette use among adult population: a cross-sectional study in Barcelona, Spain (2013-2014). BMJ open. 2014;4(8). doi: 10.1136/bmjopen-2014-005894. PubMed PMID: WOS:000340779400061.

368. Martinez-Sanchez JM, Fu M, Carlos Martin-Sanchez J, Ballbe M, Salto E, Fernandez E. Perception of electronic cigarettes in the general population: does their usefulness outweigh their risks? BMJ open. 2015;5(11). doi: 10.1136/bmjopen-2015-009218. PubMed PMID: WOS:000368840100105.

369. Marynak KL, Gammon DG, Rogers T, Coats EM, Singh T, King BA. Sales of Nicotine-Containing Electronic Cigarette Products: United States, 2015. American Journal of Public Health. 2017;107(5):702-5. doi: 10.2105/ajph.2017.303660. PubMed PMID: WOS:000404385600035.

370. Mayor S. Young adults using e-cigarettes are more likely to progress to smoking, study shows. BMJ. 2015;351:h4802. Epub 2015/09/12. doi: 10.1136/bmj.h4802. PubMed PMID: 26353973.

371. Mays D, Arrazola RA, Tworek C, Rolle IV, Neff LJ, Portnoy DB. Openness to Using Non-cigarette Tobacco Products Among U.S. Young Adults. American Journal of Preventive Medicine. 2016;50(4):528-34. Epub 2015/11/10. doi: 10.1016/j.amepre.2015.08.015. PubMed PMID: 26549502; PubMed Central PMCID: PMCPMC5253657.

372. Mazza D, McGrath-Morrow SA, Collaco JM. Use and Perceptions of Electronic Cigarettes Among Caregivers of Infants and Children with Bronchopulmonary Dysplasia. Pediatric Allergy Immunology and Pulmonology. 2017;30(3):141-7. doi: 10.1089/ped.2017.0771. PubMed PMID: WOS:000417377200002.

373. McCabe SE, West BT, Veliz P, Boyd CJ. E-cigarette use, cigarette smoking, dual use, and problem behaviors among u.S. Adolescents: Results from a national survey. Journal of Adolescent Health. 2017. doi: 10.1016/j.jadohealth.2017.02.004. PubMed PMID: 2017-15815-001.

374. McCarthy M. Far more non-smoking US students are trying e-cigarettes, figures show. BMJ. 2014;349:g5353. Epub 2014/08/31. doi: 10.1136/bmj.g5353. PubMed PMID: 25171814.

375. McCarthy M. Youth exposure to e-cigarette advertising on US television soars. BMJ. 2014;348:g3703.

376. McCarthy M. Teens who use e-cigarettes are more likely to take up smoking, US study finds. BMJ. 2015;351:h4471. Epub 2015/08/21. doi: 10.1136/bmj.h4471. PubMed PMID: 26289777.

377. McCarthy M. Teens’e-cigarette use rises as spending on advertising soars, says CDC. BMJ. 2016;352:i93.

378. McCubbin A, Fallin-Bennett A, Barnett J, Ashford K. Perceptions and use of electronic cigarettes in pregnancy. Health Education Research. 2017;32(1):22-32. doi: 10.1093/her/cyw059. PubMed PMID: 121153763. Language: English. Entry Date: 20170209. Revision Date: 20170216. Publication Type: Article.

379. McDonald D. Review of The electronic cigarette, an alternative to tobacco? Drug and Alcohol Review. 2013;32(6):635-6. doi: 10.1111/dar.12054. PubMed PMID: 2013-40585-015.

380. McDonald EA, Ling PM. One of several ‘toys’ for smoking: Young adult experiences with electronic cigarettes in New York City. Tobacco Control. 2015;24(6):588-93. doi: 10.1136/tobaccocontrol-2014-051743. PubMed PMID: 2015-49461-012.

381. McGraw D. Current and future trends in electronic cigarette use. International journal of psychiatry in medicine. 2015;48(4):325-32. Epub 2014/01/01. doi: 10.2190/PM.48.4.g. PubMed PMID: 25817527.

382. McKeganey N, Dickson T. Why Don't More Smokers Switch to Using E-Cigarettes: The Views of Confirmed Smokers. International Journal of Environmental Research and Public Health. 2017;14(6). Epub 2017/06/18. doi: 10.3390/ijerph14060647. PubMed PMID: 28621763; PubMed Central PMCID: PMCPMC5486333.

383. McMillen RC, Gottlieb MA, Shaefer RMW, Winickoff JP, Klein JD. Trends in electronic cigarette use among U.S. Adults: Use is increasing in both smokers and nonsmokers. Nicotine & Tobacco Research. 2015;17(10):1195-202. doi: 10.1093/ntr/ntu213. PubMed PMID: 2015-42983-003.

384. McPherson S, Howell D, Lewis J, Barbosa-Leiker C, Bertotti Metoyer P, Roll J. Self-reported smoking effects and comparative value between cigarettes and high dose e-cigarettes in nicotine-dependent cigarette smokers. Behavioural Pharmacology. 2016;27(2-3, Specl):301-7. doi: 10.1097/fbp.0000000000000226. PubMed PMID: 2016-19910-028.

385. Meernik C, Baker HM, Lee JG, Goldstein AO. The Tobacco 21 Movement and Electronic Nicotine Delivery System Use Among Youth. Pediatrics. 2017;139(1). Epub 2016/12/13. doi: 10.1542/peds.2016-2216. PubMed PMID: 27940513.

386. Mello S, Bigman CA, Sanders-Jackson A, Tan AS. Perceived Harm of Secondhand Electronic Cigarette Vapors and Policy Support to Restrict Public Vaping: Results From a National Survey of US Adults. Nicotine & Tobacco Research. 2016;18(5):686-93. Epub 2015/10/17. doi: 10.1093/ntr/ntv232. PubMed PMID: 26470722.

387. Meltzer LR, Simrnons VN, Sutton SK, Drobes DJ, Quinn GP, Meade CD, et al. A randomized controlled trial of a smoking cessation self-help intervention for dual users of tobacco cigarettes and E-cigarettes: Intervention development and research design. Contemporary Clinical Trials. 2017;60:56-62. doi: 10.1016/j.cct.2017.06.014. PubMed PMID: WOS:000407981100007.

388. Meyers MJ, Delucchi K, Halpern-Felsher B. Access to Tobacco Among California High School Students: The Role of Family Members, Peers, and Retail Venues. Journal of Adolescent Health. 2017;61(3):385-8. doi: 10.1016/j.jadohealth.2017.04.012. PubMed PMID: WOS:000415371100018.

389. Miao S, Beach ES, Sommer TJ, Zimmerman JB, Jordt S-E. High-intensity sweeteners in alternative tobacco products. Nicotine & Tobacco Research. 2016;18(11):2169-73. doi: 10.1093/ntr/ntw141. PubMed PMID: 2016-50445-017.

390. Miech R, Patrick ME, O'malley PM, Johnston LD. What are kids vaping? Results from a national survey of US adolescents. Tobacco control. 2017;26(4):386-91.

391. Miech RA, O'Malley PM, Johnston LD, Patrick ME. E-cigarettes and the drug use patterns of adolescents. Nicotine & Tobacco Research. 2016;18(5):654-9. doi: 10.1093/ntr/ntv217. PubMed PMID: 2016-40277-021.

392. Miller A. Nicotine poisoning increase due to e-cigarettes. Canadian Medical Association Journal. 2014;186(10):E367-E. doi: 10.1503/cmaj.109-4818. PubMed PMID: WOS:000339058400018.

393. Moheimani RS, Bhetraratana M, Peters KM, Yang BK, Yin F, Gornbein J, et al. Sympathomimetic Effects of Acute E-Cigarette Use: Role of Nicotine and Non-Nicotine Constituents. Journal of the American Heart Association. 2017;6(9). doi: 10.1161/jaha.117.006579. PubMed PMID: WOS:000411362700050.

394. Moore GF, Littlecott HJ, Moore L, Ahmed N, Holliday J. E-cigarette use and intentions to smoke among 10-11-year-old never-smokers in Wales. Tobacco Control. 2016;25(2):147-52. doi: 10.1136/tobaccocontrol-2014-052011. PubMed PMID: 2016-47739-002.

395. Moore M, McKee M, Daube M. Harm reduction and e-cigarettes: Distorting the approach. Journal of Public Health Policy. 2016;37(4):403-10. Epub 2017/02/17. doi: 10.1057/s41271-016-0031-2. PubMed PMID: 28202930.

396. Morean ME, Kong G, Camenga DR, Cavallo DA, Krishnan-Sarin S. High School Students' Use of Electronic Cigarettes to Vaporize Cannabis. Pediatrics. 2015;136(4):611-6. Epub 2015/09/09. doi: 10.1542/peds.2015-1727. PubMed PMID: 26347431; PubMed Central PMCID: PMCPMC4586732.

397. Morean ME, Kong G, Camenga DR, Cavallo DA, Simon P, Krishnan-Sarin S. Latent class analysis of current e-cigarette and other substance use in high school students. Drug and Alcohol Dependence. 2016;161:292-7. doi: 10.1016/j.drugalcdep.2016.02.018. PubMed PMID: 2016-10234-001.

398. Morean ME, Kong G, Cavallo DA, Camenga DR, Krishnan-Sarin S. Nicotine concentration of e-cigarettes used by adolescents. Drug and Alcohol Dependence. 2016;167:224-7. doi: 10.1016/j.drugalcdep.2016.06.031. PubMed PMID: WOS:000385325100030.

399. Morean ME, Lipshie N, Josephson M, Foster D. Predictors of adult e-cigarette users vaporizing cannabis using e-cigarettes and vape-pens. Substance Use & Misuse. 2017;52(8):974-81. doi: 10.1080/10826084.2016.1268162. PubMed PMID: 2017-19675-002.

400. Morphett K, Carter A, Hall W, Gartner C. Medicalisation, smoking and e-cigarettes: evidence and implications. Tobacco Control. 2016. doi: 10.1136/tobaccocontrol-2016-053348.

401. Mowls DS. Cigarette smoking, electronic nicotine product use, and biomarkers of exposure among American Indians in the Southern Plains. US: ProQuest Information & Learning; 2018.

402. Murthy VH. E-Cigarette Use Among Youth and Young Adults A Major Public Health Concern. JAMA Pediatrics. 2017;171(3):209-10. doi: 10.1001/jamapediatrics.2016.4662. PubMed PMID: WOS:000396202400004.

403. Nădăşan V, Foley KL, Pénzes M, Paulik E, Mihăicuţă Ş, Ábrám Z, et al. Use of electronic cigarettes and alternative tobacco products among Romanian adolescents. International Journal of Public Health. 2016;61(2):199-207. doi: 10.1007/s00038-015-0774-8. PubMed PMID: 2016-00383-001.

404. Nagelhout GE, Heijndijk SM, Cummings KM, Willemsen MC, van den Putte B, Heckman BW, et al. E-cigarette advertisements, and associations with the use of e-cigarettes and disapproval or quitting of smoking: Findings from the International Tobacco Control (ITC) Netherlands Survey. International Journal of Drug Policy. 2016;29:73-9.

405. Naughton F. Daily e-cigarette use increases quit attempts and reduces smoking with no effect on cessation. Evidence-based nursing. 2016;19(1):18. Epub 2015/09/18. doi: 10.1136/eb-2015-102173. PubMed PMID: 26376905.

406. Nayak P, Kemp CB, Redmon P. A Qualitative Study of Vape Shop Operators' Perceptions of Risks and Benefits of E-Cigarette Use and Attitude Toward Their Potential Regulation by the US Food and Drug Administration, Florida, Georgia, South Carolina, or North Carolina, 2015. Preventing chronic disease. 2016;13:E68. Epub 2016/05/20. doi: 10.5888/pcd13.160071. PubMed PMID: 27197081; PubMed Central PMCID: PMCPMC4877178.

407. Nayak P, Pechacek TF, Weaver SR, Eriksen MP. Electronic nicotine delivery system dual use and intention to quit smoking: Will the socioeconomic gap in smoking get greater? Addictive Behaviors. 2016;61:112-6. doi: 10.1016/j.addbeh.2016.05.020. PubMed PMID: 2016-31384-020.

408. Nichols TT, Foulds J, Yingst JM, Veldheer S, Hrabovsky S, Richie J, et al. Cue-reactivity in experienced electronic cigarette users: Novel stimulus videos and a pilot fMRI study. Brain Research Bulletin. 2016;123:23-32. doi: 10.1016/j.brainresbull.2015.10.003. PubMed PMID: 2015-49401-001.

409. Nicksic NE, Snell LM, Rudy AK, Cobb CO, Barnes AJ. Tobacco Marketing, E-cigarette Susceptibility, and Perceptions among Adults. American Journal of Health Behavior. 2017;41(5):579-90. doi: 10.5993/ajhb.41.5.7. PubMed PMID: WOS:000407973600007.

410. Noland M, Ickes MJ, Rayens MK, Butler K, Wiggins AT, Hahn EJ. Social influences on use of cigarettes, e-cigarettes, and hookah by college students. Journal of American College Health. 2016;64(4):319-28. doi: 10.1080/07448481.2016.1138478. PubMed PMID: 2016-21851-006.

411. Nonnemaker J, Kim AE, Lee YO, MacMonegle A. Quantifying how smokers value attributes of electronic cigarettes. Tobacco Control. 2016;25(e1):e37-e43. doi: 10.1136/tobaccocontrol-2015-052511. PubMed PMID: 26546152.

412. Nowariak ENS, Lien RK, Boyle RG, Amato MS, Beebe LA. E-cigarette use among treatment-seeking smokers: Moderation of abstinence by use frequency. Addictive Behaviors. 2018;77:137-42. doi: 10.1016/j.addbeh.2017.09.023. PubMed PMID: 2017-52967-024.

413. O'Connor R, Rousu MC, Bansal-Travers M, Vogl L, Corrigan JR. Using experimental auctions to examine demand for e-cigarettes. Nicotine & Tobacco Research. 2017;19(6):767-72. PubMed PMID: 2017-30525-016.

414. Oncken CA, Litt MD, McLaughlin LD, Burki NA. Nicotine Concentrations With Electronic Cigarette Use: Effects of Sex and Flavor. Nicotine & Tobacco Research. 2015;17(4):473-8. doi: 10.1093/ntr/ntu232. PubMed PMID: WOS:000353903000013.

415. Ooms GI, Bosdriesz JR, Portrait FRM, Kunst AE. Sociodemographic differences in the use of electronic nicotine delivery systems in the European Union. Nicotine & Tobacco Research. 2016;18(5):724-9. doi: 10.1093/ntr/ntv215. PubMed PMID: 2016-40277-031.

416. Orr MS. Electronic cigarettes in the USA: a summary of available toxicology data and suggestions for the future. Tobacco Control. 2014;23 Suppl 2:ii18-22. Epub 2014/04/16. doi: 10.1136/tobaccocontrol-2013-051474. PubMed PMID: 24732158; PubMed Central PMCID: PMCPMC3995288.

417. Owotomo O, Maslowsky J, Loukas A. Perceptions of the harm and addictiveness of conventional cigarette smoking among adolescent e-cigarette users. Journal of Adolescent Health. 2017. doi: 10.1016/j.jadohealth.2017.08.007. PubMed PMID: 2017-47622-001.

418. Owusu D, Aibangbee J, Collins C, Robertson C, Wang L, Littleton MA, et al. The use of e-cigarettes among school-going adolescents in a predominantly rural environment of central Appalachia. Journal of Community Health: The Publication for Health Promotion and Disease Prevention. 2017;42(3):624-31. doi: 10.1007/s10900-016-0297-0. PubMed PMID: 2017-19463-025.

419. Paek H-J, Kim S, Hove T, Huh JY. Reduced harm or another gateway to smoking? Source, message, and information characteristics of e-cigarette videos on YouTube. Journal of Health Communication. 2014;19(5):545-60.

420. Palipudi KM, Mbulo L, Morton J, Mbulo L, Bunnell R, Blutcher-Nelson G, et al. Awareness and current use of electronic cigarettes in Indonesia, Malaysia, Qatar, and Greece: Findings from 2011–2013 Global Adult Tobacco Surveys. Nicotine & Tobacco Research. 2016;18(4):501-7. doi: 10.1093/ntr/ntv081. PubMed PMID: 2016-40275-019.

421. Park J-Y, Seo D-C, Lin H-C. E-cigarette use and intention to initiate or quit smoking among US youths. American Journal of Public Health. 2016;106(4):672-8. doi: 10.2105/ajph.2015.302994. PubMed PMID: 2016-19979-002.

422. Park S, Lee H, Min S. Factors associated with electronic cigarette use among current cigarette-smoking adolescents in the Republic of Korea. Addictive Behaviors. 2017;69:22-6. doi: 10.1016/j.addbeh.2017.01.002. PubMed PMID: WOS:000397372000004.

423. Park SH, Duncan DT, Shahawy OE, Lee L, Shearston JA, Tamura K, et al. Characteristics of adults who switched from cigarette smoking to e-cigarettes. American Journal of Preventive Medicine. 2017;53(5):652-60. doi: 10.1016/j.amepre.2017.06.033. PubMed PMID: 2017-47482-014.

424. Park SH, Lee L, Shearston JA, Weitzman M. Patterns of electronic cigarette use and level of psychological distress. PLOS ONE. 2017;12(3):e0173625. Epub 2017/03/10. doi: 10.1371/journal.pone.0173625. PubMed PMID: 28278239; PubMed Central PMCID: PMCPMC5344459.

425. Pasquereau A, Guignard R, Andler R, Viet N-T. Electronic cigarettes, quit attempts and smoking cessation: a 6-month follow-up. Addiction. 2017;112(9):1620-8. doi: 10.1111/add.13869. PubMed PMID: WOS:000406975400015.

426. Patel D, Davis KC, Cox S, Bradfield B, King BA, Shafer P, et al. Reasons for current E-cigarette use among U.S. adults. Preventive Medicine. 2016;93:14-20. doi: 10.1016/j.ypmed.2016.09.011. PubMed PMID: 2016-58371-004.

427. Patrick ME, Miech RA, Carlier C, O'Malley PM, Johnston LD, Schulenberg JE. Self-reported reasons for vaping among 8th, 10th, and 12th graders in the US: Nationally-representative results. Drug and Alcohol Dependence. 2016;165:275-8. doi: 10.1016/j.drugalcdep.2016.05.017. PubMed PMID: 2016-28660-001.

428. Pearson JL, Amato MS, Wang X, Zhao K, Cha S, Cohn AM, et al. How US smokers refer to e-cigarettes: An examination of user-generated posts from a web-based smoking cessation intervention, 2008–2015. Nicotine & Tobacco Research. 2017;19(2):253-7. doi: 10.1093/ntr/ntw206. PubMed PMID: 2017-30934-016.

429. Pearson JL, Richardson A, Niaura RS, Vallone DM, Abrams DB. e-Cigarette Awareness, Use, and Harm Perceptions in US Adults. American Journal of Public Health. 2012;102(9):1758-66. doi: 10.2105/ajph.2011.300526. PubMed PMID: WOS:000307913400022.

430. Pearson JL, Stanton CA, Cha S, Niaura RS, Luta G, Graham AL. E-cigarettes and smoking cessation: Insights and cautions from a secondary analysis of data from a study of online treatment-seeking smokers. Nicotine & Tobacco Research. 2015;17(10):1219-27. doi: 10.1093/ntr/ntu269. PubMed PMID: 2015-42983-006.

431. Pénzes M, Foley KL, Balázs P, Urbán R. Intention to experiment with e-cigarettes in a cross-sectional survey of undergraduate university students in Hungary. Substance Use & Misuse. 2016;51(9):1083-92. doi: 10.3109/10826084.2016.1160116. PubMed PMID: 2016-30681-002.

432. Pepper JK, Brewer NT. Electronic nicotine delivery system (electronic cigarette) awareness, use, reactions and beliefs: A systematic review. Tobacco Control. 2014;23(5):375-84. doi: 10.1136/tobaccocontrol-2013-051122. PubMed PMID: 2014-34874-001.

433. Pepper JK, Emery SL, Ribisl KM, Brewer NT. How U.S. Adults Find Out About Electronic Cigarettes: Implications for Public Health Messages. Nicotine & Tobacco Research. 2014;16(8):1140-4. doi: 10.1093/ntr/ntu060. PubMed PMID: WOS:000339949600014.

434. Pepper JK, Emery SL, Ribisl KM, Rini CM, Brewer NT. How risky is it to use e-cigarettes? Smokers' beliefs about their health risks from using novel and traditional tobacco products. Journal of Behavioral Medicine. 2015;38(2):318-26. doi: 10.1007/s10865-014-9605-2. PubMed PMID: WOS:000351135900013.

435. Pepper JK, Emery SL, Ribisl KM, Southwell BG, Brewer NT. Effects of advertisements on smokers’ interest in trying e-cigarettes: the roles of product comparison and visual cues. Tobacco control. 2014;23(suppl 3):iii31-iii6.

436. Pepper JK, Reiter PL, McRee A-L, Cameron LD, Gilkey MB, Brewer NT. Adolescent Males' Awareness of and Willingness to Try Electronic Cigarettes. Journal of Adolescent Health. 2013;52(2):144-50. doi: 10.1016/j.jadohealth.2012.09.014. PubMed PMID: WOS:000313560100003.

437. Pepper JK, Ribisl KM, Brewer NT. Adolescents' interest in trying flavoured e-cigarettes. Tobacco Control. 2016;25(supp 2):ii62-ii6. doi: 10.1136/tobaccocontrol-2016-053174. PubMed PMID: WOS:000390589800009.

438. Pepper JK, Ribisl KM, Emery SL, Brewer NT. Reasons for Starting and Stopping Electronic Cigarette Use. International Journal of Environmental Research and Public Health. 2014;11(10):10345-61. doi: 10.3390/ijerph111010345. PubMed PMID: WOS:000344358700024.

439. Persoskie A, O'Brien EK, Nguyen AB, Tworek C. Measuring youth beliefs about the harms of e-cigarettes and smokeless tobacco compared to cigarettes. Addictive Behaviors. 2017;70:7-13. doi: 10.1016/j.addbeh.2017.01.033. PubMed PMID: 2017-13437-003.

440. Pesko MF, Huang J, Johnston LD, Chaloupka FJ. E‐cigarette Price Sensitivity Among Middle and High School Students: Evidence from Monitoring the Future. Addiction. 2017.

441. Pesko MF, Kenkel DS, Wang H, Hughes JM. The effect of potential electronic nicotine delivery system regulations on nicotine product selection. Addiction. 2016;111(4):734-44. Epub 2015/12/08. doi: 10.1111/add.13257. PubMed PMID: 26639526; PubMed Central PMCID: PMCPMC4801654.

442. Pesko MF, Robarts AMT. Adolescent Tobacco Use in Urban Versus Rural Areas of the United States: The Influence of Tobacco Control Policy Environments. Journal of Adolescent Health. 2017;61(1):70-6. doi: 10.1016/j.jadohealth.2017.01.019. PubMed PMID: WOS:000405362300010.

443. Peters EN, Harrell PT, Hendricks PS, O'Grady KE, Pickworth WB, Vocci FJ. Electronic Cigarettes in Adults in Outpatient Substance Use Treatment: Awareness, Perceptions, Use, and Reasons for Use. American Journal on Addictions. 2015;24(3):233-9. doi: 10.1111/ajad.12206. PubMed PMID: WOS:000353414300009.

444. Peters RJ, Jr., Meshack A, Lin M-T, Hill M, Abughosh S. The social norms and beliefs of teenage male electronic cigarette use. Journal of Ethnicity in Substance Abuse. 2013;12(4):300-7. doi: 10.1080/15332640.2013.819310. PubMed PMID: 2013-40352-003.

445. Pineiro B, Correa JB, Simmons VN, Harrell PT, Menzie NS, Unrod M, et al. Gender differences in use and expectancies of e-cigarettes: Online survey results. Addictive Behaviors. 2016;52:91-7. doi: 10.1016/j.addbeh.2015.09.006. PubMed PMID: WOS:000365377100014.

446. Pokhrel P, Fagan P, Herzog TA, Chen Q, Muranaka N, Kehl L, et al. E-cigarette advertising exposure and implicit attitudes among young adult non-smokers. Drug and alcohol dependence. 2016;163:134-40.

447. Pokhrel P, Fagan P, Kehl L, Herzog TA. Receptivity to e-cigarette marketing, harm perceptions, and e-cigarette use. American Journal of Health Behavior. 2015;39(1):121-31. Epub 2014/10/08. doi: 10.5993/ajhb.39.1.13. PubMed PMID: 25290604; PubMed Central PMCID: PMCPMC4877176.

448. Pokhrel P, Herzog TA, Muranaka N, Fagan P. Young adult e-cigarette users' reasons for liking and not liking e-cigarettes: A qualitative study. Psychology & Health. 2015;30(12):1450-69. doi: 10.1080/08870446.2015.1061129. PubMed PMID: WOS:000362124200004.

449. Pokhrel P, Little MA, Fagan P, Muranaka N, Herzog TA. Electronic cigarette use outcome expectancies among college students. Addictive Behaviors. 2014;39(6):1062-5. doi: 10.1016/j.addbeh.2014.02.014. PubMed PMID: 2014-09714-001.

450. Polosa R, Caponnetto P, Cibella F, Le-Houezec J. Quit and smoking reduction rates in vape shop consumers: a prospective 12-month survey. International Journal of Environmental Research and Public Health. 2015;12(4):3428-38. Epub 2015/03/27. doi: 10.3390/ijerph120403428. PubMed PMID: 25811767; PubMed Central PMCID: PMCPMC4410194.

451. Popova L, Ling PM. Nonsmokers' responses to new warning labels on smokeless tobacco and electronic cigarettes: an experimental study. BMC public health. 2014;14:997. Epub 2014/09/26. doi: 10.1186/1471-2458-14-997. PubMed PMID: 25253295; PubMed Central PMCID: PMCPMC4190284.

452. Porter L, Duke J, Hennon M, Dekevich D, Crankshaw E, Homsi G, et al. Electronic Cigarette and Traditional Cigarette Use among Middle and High School Students in Florida, 2011-2014. PLOS ONE. 2015;10(5). doi: 10.1371/journal.pone.0124385. PubMed PMID: WOS:000354544200038.

453. Postolache P, Nemes RM, Serban RI, Rad RM, Stratulat IS. ELECTRONIC CIGARETTE--A WAY OF SMOKING CESSATION? Revista medico-chirurgicala a Societatii de Medici si Naturalisti din Iasi. 2015;119(2):510-6. Epub 2015/07/25. PubMed PMID: 26204660.

454. Potera C. E-Cigarettes May Steer Teens to Smoking. American Journal of Nursing. 2015;115(11):15. Epub 2015/10/29. doi: 10.1097/01.naj.0000473300.18534.18. PubMed PMID: 26510056.

455. Pratt SI, Sargent J, Daniels L, Santos MM, Brunette M. Appeal of electronic cigarettes in smokers with serious mental illness. Addictive Behaviors. 2016;59:30-4. doi: 10.1016/j.addbeh.2016.03.009. PubMed PMID: 2016-21384-007.

456. Printz C. Smoke signals: New reports reflect "staggering" increases in youth e-cigarette usage. Cancer. 2015;121(22):3927-8. Epub 2015/11/05. doi: 10.1002/cncr.29006. PubMed PMID: 26536525.

457. Printz C. E-cigarettes expand tobacco use among adolescents. Cancer. 2017;123(8):1287-. doi: 10.1002/cncr.30715. PubMed PMID: WOS:000398809700003.

458. Prochaska JJ, Grana RA. E-cigarette use among smokers with serious mental illness. PLOS ONE. 2014;9(11):e113013. Epub 2014/11/25. doi: 10.1371/journal.pone.0113013. PubMed PMID: 25419703; PubMed Central PMCID: PMCPMC4242512.

459. Pu J, Zhang X. Exposure to advertising and perception, interest, and use of e-cigarettes among adolescents: findings from the US National Youth Tobacco Survey. Perspectives in Public Health. 2017;137(6):322-5. doi: 10.1177/1757913917722747. PubMed PMID: WOS:000413942600013.

460. Pulvers K, Hayes RB, Scheuermann TS, Romero DR, Emami AS, Resnicow K, et al. Tobacco use, quitting behavior, and health characteristics among current electronic cigarette users in a national tri-ethnic adult stable smoker sample. Nicotine & Tobacco Research. 2015;17(9):1085-95. doi: 10.1093/ntr/ntu241. PubMed PMID: 2015-42980-005.

461. Rahman MA, Hann N, Wilson A, Mnatzaganian G, Worrall-Carter L. E-Cigarettes and Smoking Cessation: Evidence from a Systematic Review and Meta-Analysis. PLOS ONE. 2015;10(3). doi: 10.1371/journal.pone.0122544. PubMed PMID: WOS:000352134700184.

462. Rahman MA, Hann N, Wilson A, Worrall-Carter L. Electronic cigarettes: patterns of use, health effects, use in smoking cessation and regulatory issues. Tobacco Induced Diseases. 2014;12. doi: 10.1186/1617-9625-12-21. PubMed PMID: WOS:000347485300001.

463. Ramo DE, Young-Wolff KC, Prochaska JJ. Prevalence and correlates of electronic-cigarette use in young adults: Findings from three studies over five years. Addictive Behaviors. 2015;41:142-7. doi: 10.1016/j.addbeh.2014.10.019. PubMed PMID: 2014-54131-025.

464. Rankin J. US calls for more restrictions on e-cigarettes for youth. Canadian Medical Association Journal. 2017;189(3):E131. Epub 2017/03/02. doi: 10.1503/cmaj.109-5374. PubMed PMID: 28246163; PubMed Central PMCID: PMCPMC5250529.

465. Rass O, Pacek LR, Johnson PS, Johnson MW. Characterizing use patterns and perceptions of relative harm in dual users of electronic and tobacco cigarettes. Experimental and clinical psychopharmacology. 2015;23(6):494-503. Epub 2015/09/22. doi: 10.1037/pha0000050. PubMed PMID: 26389638; PubMed Central PMCID: PMCPMC4658305.

466. Rayens MK, Ickes MJ, Butler KM, Wiggins AT, Anderson DG, Hahn EJ. University students' perceived risk of and intention to use waterpipe tobacco. Health Education Research. 2017;32(4):306-17. doi: 10.1093/her/cyx049. PubMed PMID: WOS:000407147700002.

467. Regan AK, Pronnoff G, Dube SR, Arrazola R. Electronic nicotine delivery systems: Adult use and awareness of the 'e-cigarette' in the USA. Tobacco Control. 2013;22(1):19-23. doi: 10.1136/tobaccocontrol-2011-050044. PubMed PMID: 2012-34563-004.

468. Reid JL, Rynard VL, Czoli CD, Hammond D. Who is using e-cigarettes in Canada? Nationally representative data on the prevalence of e-cigarette use among Canadians. Preventive Medicine. 2015;81:180-3. doi: 10.1016/j.ypmed.2015.08.019. PubMed PMID: WOS:000368421300027.

469. Reinhold B, Fischbein R, Bhamidipalli SS, Bryant J, Kenne DR. Associations of attitudes towards electronic cigarettes with advertisement exposure and social determinants: a cross sectional study. Tobacco Induced Diseases. 2017;15. doi: 10.1186/s12971-017-0118-y. PubMed PMID: WOS:000394394200001.

470. Rennie LJ, Bazillier-Bruneau C, Rouëssé J. Harm reduction or harm introduction? Prevalence and correlates of e-cigarette use among French adolescents. Journal of Adolescent Health. 2016;58(4):440-5. doi: 10.1016/j.jadohealth.2015.12.013. PubMed PMID: 2016-06178-001.

471. Richardson A, Ganz O, Stalgaitis C, Abrams D, Vallone D. Noncombustible tobacco product advertising: how companies are selling the new face of tobacco. Nicotine & Tobacco Research. 2014;16(5):606-14. doi: 10.1093/ntr/ntt200. PubMed PMID: 24379146.

472. Richardson A, Ganz O, Vallone D. Tobacco on the web: surveillance and characterisation of online tobacco and e-cigarette advertising. Tobacco Control. 2015;24(4):341-7.

473. Richardson A, Pearson J, Xiao H, Stalgaitis C, Vallone D. Prevalence, harm perceptions, and reasons for using noncombustible tobacco products among current and former smokers. American Journal of Public Health. 2014;104(8):1437-44. doi: 10.2105/ajph.2013.301804. PubMed PMID: 2014-29353-017.

474. Richter L. Addiction: Protect the young from e-cigarettes. Nature. 2015;524(7563):35. Epub 2015/08/08. doi: 10.1038/524035b. PubMed PMID: 26245572.

475. Riggs NR, Pentz MA. Inhibitory control and the onset of combustible cigarette, e-cigarette, and hookah use in early adolescence: The moderating role of socioeconomic status. Child Neuropsychology. 2016;22(6):679-91. doi: 10.1080/09297049.2015.1053389. PubMed PMID: 2016-28355-004.

476. Rigotti NA. e-Cigarette Use and Subsequent Tobacco Use by Adolescents: New Evidence About a Potential Risk of e-Cigarettes. JAMA. 2015;314(7):673-4. Epub 2015/08/19. doi: 10.1001/jama.2015.8382. PubMed PMID: 26284717.

477. Rigotti NA, Harrington KF, Richter K, Fellows JL, Sherman SE, Grossman E, et al. Increasing Prevalence of Electronic Cigarette Use Among Smokers Hospitalized in 5 US Cities, 2010-2013. Nicotine & Tobacco Research. 2015;17(2):236-44. doi: 10.1093/ntr/ntu138. PubMed PMID: WOS:000350142300019.

478. Rigotti NA, Wu M. Advising patients about electronic cigarettes. European heart journal. 2015;36(3):135-6. Epub 2015/03/06. PubMed PMID: 25741552.

479. Robertson L, Cameron C, McGee R, Marsh L, Hoek J. Point-of-sale tobacco promotion and youth smoking: a meta-analysis. Tobacco control. 2016:tobaccocontrol-2015-052586.

480. Roditis M, Delucchi K, Cash D, Halpern-Felsher B. Adolescents' Perceptions of Health Risks, Social Risks, and Benefits Differ Across Tobacco Products. Journal of Adolescent Health. 2016;58(5):558-66. Epub 2016/04/25. doi: 10.1016/j.jadohealth.2016.01.012. PubMed PMID: 27107909; PubMed Central PMCID: PMCPMC5072979.

481. Roditis ML, Halpern-Felsher B. Adolescents' perceptions of risks and benefits of conventional cigarettes, e-cigarettes, and marijuana: A qualitative analysis. Journal of Adolescent Health. 2015;57(2):179-85. doi: 10.1016/j.jadohealth.2015.04.002. PubMed PMID: 2015-28714-001.

482. Rodriguez E, Parron T, Alarcon R. Perceptions and use of the e-cigarette among university students. Archivos De Bronconeumologia. 2017;53(11):650-2. doi: 10.1016/j.arbres.2017.03.015. PubMed PMID: WOS:000415031200020.

483. Rogers T. Electronic nicotine delivery systems (ENDS): New evidence from the State and Community Tobacco Control Research Initiative. Tobacco Control. 2014;23(Supp 3):1-2. doi: 10.1136/tobaccocontrol-2014-051790. PubMed PMID: 2014-35246-001.

484. Rom O, Pecorelli A, Valacchi G, Reznick AZ. Are E-cigarettes a safe and good alternative to cigarette smoking? In: Valacchi G, editor. Cellular and Environmental Stressors in Biology and Medicine. Annals of the New York Academy of Sciences. 13402015. p. 65-74.

485. Rooke C, Amos A. News media representations of electronic cigarettes: an analysis of newspaper coverage in the UK and Scotland. Tobacco Control. 2014;23(6):507-12. Epub 2013/07/26. doi: 10.1136/tobaccocontrol-2013-051043. PubMed PMID: 23884011.

486. Rosbrook K, Erythropel HC, DeWinter TM, Falinski M, O'Malley S, Krishnan-Sarin S, et al. The effect of sucralose on flavor sweetness in electronic cigarettes varies between delivery devices. PLOS ONE. 2017;12(10). doi: 10.1371/journal.pone.0185334. PubMed PMID: WOS:000412029600020.

487. Rosbrook K, Green BG. Sensory effects of menthol and nicotine in an E-cigarette. Nicotine & Tobacco Research. 2016;18(7):1588-95. doi: 10.1093/ntr/ntw019. PubMed PMID: 2016-40280-007.

488. Rose SW, Barker DC, D'Angelo H, Khan T, Huang J, Chaloupka FJ, et al. The availability of electronic cigarettes in US retail outlets, 2012: Results of two national studies. Tobacco Control. 2014;23(Supp 3):10-6. doi: 10.1136/tobaccocontrol-2013-051461. PubMed PMID: 2014-35246-003.

489. Rowell TR, Reeber SL, Lee SL, Harris RA, Nethery RC, Herring AH, et al. Flavored e-cigarette liquids reduce proliferation and viability in the CALU3 airway epithelial cell line. American Journal of Physiology-Lung Cellular and Molecular Physiology. 2017;313(1):L52-L66. doi: 10.1152/ajplung.00392.2016. PubMed PMID: WOS:000404995100005.

490. Rther T, Wissen F, Linhardt A, Aichert D, Pogarell O, De Vries H. ELECTRONIC-CIGARETTES (E-CIGARETTES) IN GERMANY - A SMOKING CESSATION AID? European Psychiatry. 2014;29. PubMed PMID: WOS:000347280701409.

491. Ruether T, Wissen F, Linhardt A, Aichert DS, Pogarell O, de Vries H. Electronic Cigarettes-Attitudes and Use in Germany. Nicotine & Tobacco Research. 2016;18(5):660-9. doi: 10.1093/ntr/ntv188. PubMed PMID: WOS:000376350700022.

492. Rutten LJF, Blake KD, Agunwamba AA, Grana RA, Wilson PM, Ebbert JO, et al. Use of E-Cigarettes Among Current Smokers: Associations Among Reasons for Use, Quit Intentions, and Current Tobacco Use. Nicotine & Tobacco Research. 2015;17(10):1228-34. doi: 10.1093/ntr/ntv003. PubMed PMID: WOS:000363175500007.

493. Saddleson M, Kozlowski L, Giovino G, Goniewicz M, Mahoney M, Homish G, et al. Enjoyment and other reasons for electronic cigarette use: Results from college students in New York. Addictive behaviors. 2016;54:33-9.

494. Saddleson ML. Tobacco/nicotine use in adolescents and young adults: A focus on electronic cigarettes and measurement of non-daily tobacco cigarette use: State University of New York at Buffalo; 2015.

495. Sanders‐Jackson A, Schleicher NC, Fortmann SP, Henriksen L. Effect of warning statements in e‐cigarette advertisements: an experiment with young adults in the United States. Addiction. 2015;110(12).

496. Sanders-Jackson AN, Tan ASL, Bigman CA, Henriksen L. Knowledge about E-cigarette constituents and regulation: Results from a national survey of U.S. young adults. Nicotine & Tobacco Research. 2015;17(10):1247-54. doi: 10.1093/ntr/ntu276. PubMed PMID: 2015-42983-010.

497. Schmidt L, Reidmohr A, Harwell TS, Helgerson SD. Prevalence and reasons for initiating use of electronic cigarettes among adults in Montana, 2013. Preventing Chronic Disease. 2014;11. PubMed PMID: 2014-54769-001.

498. Schmitt CL, Lee YO, Curry LE, Farrelly MC, Rogers T. Research support for effective state and community tobacco control programme response to electronic nicotine delivery systems. Tobacco Control. 2014;23 Suppl 3:iii54-7. Epub 2014/06/18. doi: 10.1136/tobaccocontrol-2013-051460. PubMed PMID: 24935899; PubMed Central PMCID: PMCPMC4078674.

499. Schneider S, Diehl K. Vaping as a catalyst for smoking? An initial model on the initiation of electronic cigarette use and the transition to tobacco smoking among adolescents. Nicotine & Tobacco Research. 2016;18(5):647-53. doi: 10.1093/ntr/ntv193. PubMed PMID: 2016-40277-020.

500. Schneller LM, Lindgren BR, Shields PG, Hatsukami DK, O'Connor RJ. Strong preference for mint snus flavor among research participants. Addictive Behaviors Reports. 2017;6:51-5. doi: 10.1016/j.abrep.2017.07.004. PubMed PMID: 2017-50436-006.

501. Schoenborn CA, Clarke TC. Percentage of Adults Who Ever Used an E-cigarette and Percentage Who Currently Use E-cigarettes, by Age Group - National Health Interview Survey, United States, 2016. Mmwr-Morbidity and Mortality Weekly Report. 2017;66(33):892-. PubMed PMID: WOS:000408306200006.

502. Sears CG, Hart JL, Walker KL, Robertson RM. Generally Recognized as Safe: Uncertainty Surrounding E-Cigarette Flavoring Safety. International Journal of Environmental Research and Public Health. 2017;14(10). doi: 10.3390/ijerph14101274. PubMed PMID: WOS:000414763200183.

503. Seidenberg AB, Jo CL, Ribisl KM. Differences in the design and sale of e-cigarettes by cigarette manufacturers and non-cigarette manufacturers in the USA. Tobacco control. 2016;25(e1):e3-e5.

504. Seto JC, Davis JW, Taira DA. E-cigarette Use Related to Demographic Factors in Hawai'i. Hawai'i journal of medicine & public health : a journal of Asia Pacific Medicine & Public Health. 2016;75(10):295-302. Epub 2016/10/16. PubMed PMID: 27738563; PubMed Central PMCID: PMCPMC5056632.

505. Shah A, Paliwal Y, Holdford D. PREVALENCE AND CHARACTERISTICS OF E-CIGARETTE USERS AMONG COPD PATIENT POPULATION IN THE UNITED STATES. Value in Health. 2017;20(5):A201-A. PubMed PMID: WOS:000405448002354.

506. Shang C, Huang J, Chaloupka FJ, Emery SL. The impact of flavour, device type and warning messages on youth preferences for electronic nicotine delivery systems: evidence from an online discrete choice experiment. Tobacco Control. 2017. doi: 10.1136/tobaccocontrol-2017-053754. PubMed PMID: 29097588.

507. Sharfstein JM. Electronic cigarettes: gateway to understanding the FDA? The Milbank quarterly. 2015;93(2):251-4. Epub 2015/06/06. doi: 10.1111/1468-0009.12119. PubMed PMID: 26044627; PubMed Central PMCID: PMCPMC4462875.

508. Sherratt FC, Newson L, Marcus MW, Field JK, Robinson J. Perceptions towards electronic cigarettes for smoking cessation among stop smoking service users. British Journal of Health Psychology. 2016;21(2):421-33. doi: 10.1111/bjhp.12177. PubMed PMID: 2016-18073-003.

509. Shiffman S, Sembower MA, Pillitteri JL, Gerlach KK, Gitchell JG. The Impact of Flavor Descriptors on Nonsmoking Teens' and Adult Smokers' Interest in Electronic Cigarettes. Nicotine & Tobacco Research. 2015;17(10):1255-62. doi: 10.1093/ntr/ntu333. PubMed PMID: WOS:000363175500011.

510. Shih RA, Parast L, Pedersen ER, Troxel WM, Tucker JS, Miles JNV, et al. Individual, peer, and family factor modification of neighborhood-level effects on adolescent alcohol, cigarette, e-cigarette, and marijuana use. Drug and Alcohol Dependence. 2017;180:76-85. doi: 10.1016/j.drugalcdep.2017.07.014. PubMed PMID: WOS:000414816600013.

511. Siegel MB, Tanwar KL, Wood KS. Electronic Cigarettes As a Smoking-Cessation Tool Results from an Online Survey. American Journal of Preventive Medicine. 2011;40(4):472-5. doi: 10.1016/j.amepre.2010.12.006. PubMed PMID: WOS:000288319700012.

512. Simmons VN, Quinn GP, Harrell PT, Meltzer LR, Correa JB, Unrod M, et al. E-cigarette use in adults: a qualitative study of users' perceptions and future use intentions. Addiction Research & Theory. 2016;24(4):313-21. doi: 10.3109/16066359.2016.1139700. PubMed PMID: WOS:000376397100005.

513. Simonavicius E, McNeill A, Arnott D, Brose LS. What factors are associated with current smokers using or stopping e-cigarette use? Drug and Alcohol Dependence. 2017;173:139-43. doi: 10.1016/j.drugalcdep.2017.01.002. PubMed PMID: WOS:000399509300018.

514. Singh T, Kennedy S, Marynak K, Persoskie A, Melstrom P, King BA. Characteristics of Electronic Cigarette Use Among Middle and High School Students - United States, 2015. MMWR: Morbidity & Mortality Weekly Report. 2016;65(50/51):1425-9. doi: 10.15585/mmwr.mm655051a2. PubMed PMID: 120541760. Language: English. Entry Date: In Process. Revision Date: 20170125. Publication Type: journal article. Journal Subset: Biomedical.

515. Singh T, Marynak K, Arrazola RA, Cox S, Rolle IV, King BA. Vital Signs: Exposure to Electronic Cigarette Advertising Among Middle School and High School Students—United States, 2014. MMWR Morb Mortal Wkly Rep. 2016;64(52):1403-8.

516. Smiley SL, DeAtley T, Rubin LF, Harvey E, Kierstead EC, Webb Hooper M, et al. Early Subjective Sensory Experiences with "cigalike" E-cigarettes Among African American Menthol Smokers: A Qualitative Study. Nicotine & Tobacco Research. 2017. doi: 10.1093/ntr/ntx102. PubMed PMID: 28549156.

517. Smith DM, Bansal-Travers M, Huang J, Barker D, Hyland AJ, Chaloupka F. Association between use of flavoured tobacco products and quit behaviours: Findings from a cross-sectional survey of US adult tobacco users. Tobacco Control. 2016;25(Suppl 2):73-80. doi: 10.1136/tobaccocontrol-2016-053313. PubMed PMID: 2016-61125-011.

518. Smith DM, Bansal-Travers M, O'Connor RJ, Goniewicz ML, Hyland A. Associations between perceptions of e-cigarette advertising and interest in product trial amongst US adult smokers and non-smokers: results from an internet-based pilot survey. Tobacco Induced Diseases. 2015;13. doi: 10.1186/s12971-015-0039-6. PubMed PMID: WOS:000357251600001.

519. Snider SE, Cummings KM, Bickel WK. Behavioral economic substitution between conventional cigarettes and e-cigarettes differs as a function of the frequency of e-cigarette use. Drug and Alcohol Dependence. 2017;177:14-22. doi: 10.1016/j.drugalcdep.2017.03.017. PubMed PMID: WOS:000407666100002.

520. Sokolovsky AW. MOTIVES FOR CONTINUATION AMONG YOUNG ADULT USERS OF ELECTRONIC NICOTINE DELIVERY SYSTEMS: THE ROLE OF IDENTITY. Annals of Behavioral Medicine. 2017;51:S426-S7. PubMed PMID: WOS:000398947200282.

521. Soneji S, Sung H-Y, Primack B, Pierce JP, Sargent J. Problematic assessment of the impact of vaporized nicotine product initiation in the United States. Nicotine & Tobacco Research. 2017;19(2):264-5. doi: 10.1093/ntr/ntw232. PubMed PMID: 2017-30934-018.

522. Soule EK, Lopez AA, Guy MC, Cobb CO. Reasons for using flavored liquids among electronic cigarette users: A concept mapping study. Drug and Alcohol Dependence. 2016;166:168-76.

523. Soule EK, Maloney SF, Guy MC, Eissenberg T, Fagan P. User Identified Positive Outcome Expectancies of Electronic Cigarette Use: A Concept Mapping Study. Psychology of Addictive Behaviors. 2017;31(3):343-53. doi: 10.1037/adb0000263. PubMed PMID: WOS:000400899700012.

524. Soule EK, Nasim A, Rosas S. Adverse Effects of Electronic Cigarette Use: A Concept Mapping Approach. Nicotine & Tobacco Research. 2016;18(5):678-85. Epub 2015/11/14. doi: 10.1093/ntr/ntv246. PubMed PMID: 26563262.

525. Soule EK, Rosas SR, Nasim A. Reasons for electronic cigarette use beyond cigarette smoking cessation: A concept mapping approach. Addictive Behaviors. 2016;56:41-50. doi: 10.1016/j.addbeh.2016.01.008. PubMed PMID: 2016-06463-008.

526. Soussy S, Ahmad E-H, Baalbaki R, Salman R, Shihadeh A, Saliba NA. Detection of 5-hydroxymethylfurfural and furfural in the aerosol of electronic cigarettes. Tobacco Control. 2016;25(suppl 2):ii88-ii93. doi: 10.1136/tobaccocontrol-2016-053220.

527. Spears CA, Jones DM, Weaver SR, Pechacek TF, Eriksen MP. Use of Electronic Nicotine Delivery Systems among Adults with Mental Health Conditions, 2015. International Journal of Environmental Research and Public Health. 2016;14(1). Epub 2016/12/28. doi: 10.3390/ijerph14010010. PubMed PMID: 28025560; PubMed Central PMCID: PMCPMC5295261.

528. Spindle TR, Hiler MM, Cooke ME, Eissenberg T, Kendler KS, Dick DM. Electronic cigarette use and uptake of cigarette smoking: A longitudinal examination of U.S. college students. Addictive Behaviors. 2017;67:66-72. doi: 10.1016/j.addbeh.2016.12.009. PubMed PMID: 2017-03082-013.

529. St.Helen G, Dempsey DA, Havel CM, Jacob P, Benowitz NL. Impact of e-liquid flavors on nicotine intake and pharmacology of e-cigarettes. Drug and Alcohol Dependence. 2017;178:391-8. doi: 10.1016/j.drugalcdep.2017.05.042. PubMed PMID: 2017-34715-056.

530. Stanbrook MB. Electronic cigarettes and youth: a gateway that must be shut. Canadian Medical Association Journal. 2016;188(11):785. Epub 2016/07/20. doi: 10.1503/cmaj.160728. PubMed PMID: 27431301; PubMed Central PMCID: PMCPMC4978568.

531. Stein MD, Caviness CM, Grimone K, Audet D, Borges A, Anderson BJ. E-cigarette knowledge, attitudes, and use in opioid dependent smokers. Journal of Substance Abuse Treatment. 2015;52:73-7. doi: 10.1016/j.jsat.2014.11.002. PubMed PMID: 2014-54621-001.

532. Stenger N, Chailleux E. [Survey on the use of electronic cigarettes and tobacco among children in middle and high school]. Revue des maladies respiratoires. 2016;33(1):56-62. Epub 2015/06/15. doi: 10.1016/j.rmr.2015.05.003. PubMed PMID: 26071978.

533. Stoklosa M, Drope J, Chaloupka FJ. Prices and e-cigarette demand: Evidence from the European Union. Nicotine & Tobacco Research. 2016;18(10):1973-80. doi: 10.1093/ntr/ntw109. PubMed PMID: 2016-46012-008.

534. Strong DR, Myers M, Linke S, Leas E, Hofstetter R, Edland S, et al. Gender differences influence overweight smokers' experimentation with electronic nicotine delivery systems. Addictive Behaviors. 2015;49:20-5. Epub 2015/06/04. doi: 10.1016/j.addbeh.2015.05.003. PubMed PMID: 26036665; PubMed Central PMCID: PMCPMC4478105.

535. Surís J-C, Berchtold A, Akre C. Reasons to use e-cigarettes and associations with other substances among adolescents in Switzerland. Drug & Alcohol Dependence. 2015;153:140-4. doi: 10.1016/j.drugalcdep.2015.05.034. PubMed PMID: 109604366. Language: English. Entry Date: 20150923. Revision Date: 20160220. Publication Type: journal article. Journal Subset: Biomedical.

536. Sussan TE, Shahzad FG, Tabassum E, Cohen JE, Wise RA, Blaha MJ, et al. Electronic cigarette use behaviors and motivations among smokers and non-smokers. BMC public health. 2017;17. doi: 10.1186/s12889-017-4671-3. PubMed PMID: WOS:000409561200001.

537. Sussman S, Garcia R, Cruz TB, Baezconde-Garbanati L, Pentz MA, Unger JB. Consumers’ perceptions of vape shops in Southern California: an analysis of online Yelp reviews. Tobacco Induced Diseases. 2014;12(1):22.

538. Sutfin EL, McCoy TP, Morrell HER, Hoeppner BB, Wolfson M. Electronic cigarette use by college students. Drug and Alcohol Dependence. 2013;131(3):214-21. doi: 10.1016/j.drugalcdep.2013.05.001. PubMed PMID: 2013-20665-001.

539. Sutfin EL, Reboussin BA, Debinski B, Wagoner KG, Spangler J, Wolfson M. The Impact of Trying Electronic Cigarettes on Cigarette Smoking by College Students: A Prospective Analysis. American Journal of Public Health. 2015;105(8):e83-9. Epub 2015/06/13. doi: 10.2105/ajph.2015.302707. PubMed PMID: 26066954; PubMed Central PMCID: PMCPMC4504281.

540. Sutherland R, Sindicich N, Entwistle G, Whittaker E, Peacock A, Matthews A, et al. Tobacco and e-cigarette use amongst illicit drug users in Australia. Drug and Alcohol Dependence. 2016;159:35-41. doi: 10.1016/j.drugalcdep.2015.10.035. PubMed PMID: 2015-57244-001.

541. Syamlal G, King BA, Mazurek JM. Tobacco Use Among Working Adults - United States, 2014-2016. Mmwr-Morbidity and Mortality Weekly Report. 2017;66(42):1130-5. doi: 10.15585/mmwr.mm6642a2. PubMed PMID: WOS:000413771700002.

542. Talih S, Balhas Z, Eissenberg T, Salman R, Karaoghlanian N, El Hellani A, et al. Effects of user puff topography, device voltage, and liquid nicotine concentration on electronic cigarette nicotine yield: measurements and model predictions. Nicotine & Tobacco Research. 2015;17(2):150-7. Epub 2014/09/05. doi: 10.1093/ntr/ntu174. PubMed PMID: 25187061; PubMed Central PMCID: PMCPMC4837998.

543. Tamimi N. Knowledge, attitudes and beliefs towards e-cigarettes among e-cigarette users and stop smoking advisors in south east england: A qualitative study. Primary Health Care Research and Development. 2017. doi: 10.1017/s1463423617000445. PubMed PMID: 2017-34024-001.

544. Tan AS, Bigman CA. E-cigarette awareness and perceived harmfulness: prevalence and associations with smoking-cessation outcomes. American Journal of Preventive Medicine. 2014;47(2):141-9. Epub 2014/05/06. doi: 10.1016/j.amepre.2014.02.011. PubMed PMID: 24794422; PubMed Central PMCID: PMCPMC4107147.

545. Tan ASL, Bigman CA. 'E-cigarette awareness and perceived harmfulness: Prevalence and associations with smoking-cessation outcomes': Correction. American Journal of Preventive Medicine. 2016;50(5):674-6. doi: 10.1016/j.amepre.2016.01.006. PubMed PMID: 2016-20192-021.

546. Tan ASL, Bigman CA, Sanders-Jackson A. Sociodemographic correlates of self-reported exposure to e-cigarette communications and its association with public support for smoke-free and vape-free policies: results from a national survey of US adults. Tobacco Control. 2015;24(6):574-81. doi: 10.1136/tobaccocontrol-2014-051685. PubMed PMID: WOS:000363470600017.

547. Tan ASL, Lee C-j, Bigman CA. Comparison of beliefs about e-cigarettes' harms and benefits among never users and ever users of e-cigarettes. Drug and Alcohol Dependence. 2016;158:67-75. doi: 10.1016/j.drugalcdep.2015.11.003. PubMed PMID: WOS:000368566200009.

548. Temple JR, Shorey RC, Lu Y, Torres E, Stuart GL, Le VD. E-cigarette use of young adults motivations and associations with combustible cigarette alcohol, marijuana, and other illicit drugs. American Journal on Addictions. 2017;26(4):343-8. doi: 10.1111/ajad.12530. PubMed PMID: WOS:000401162900006.

549. Thatcher A. E-cigarettes more popular than tobacco among youth. Canadian Medical Association Journal. 2015;187(6):E184. Epub 2015/03/11. doi: 10.1503/cmaj.109-5010. PubMed PMID: 25754709; PubMed Central PMCID: PMCPMC4387055.

550. Thirlway F. The type of e-cigarette affects its usefulness in smoking cessation. BMJ. 2015;351:h3898. Epub 2015/07/25. doi: 10.1136/bmj.h3898. PubMed PMID: 26205367.

551. Thrasher JF, Abad-Vivero EN, Barrientos-Gutíerrez I, Pérez-Hernández R, Reynales-Shigematsu LM, Mejía R, et al. Prevalence and correlates of e-cigarette perceptions and trial among early adolescents in Mexico. Journal of Adolescent Health. 2016;58(3):358-65. doi: 10.1016/j.jadohealth.2015.11.008. PubMed PMID: 2016-10365-010.

552. Thrul J, Ramo DE. Cessation strategies young adult smokers use after participating in a Facebook intervention. Substance Use & Misuse. 2017;52(2):259-64. doi: 10.1080/10826084.2016.1223690. PubMed PMID: 2016-60808-015.

553. Tierney PA, Karpinski CD, Brown JE, Luo W, Pankow JF. Flavour chemicals in electronic cigarette fluids. Tobacco control. 2016;25(E1):e10-e5. doi: 10.1136/tobaccocontrol-2014-052175.

554. Tomashefski A. The perceived effects of electronic cigarettes on health by adult users: A state of the science systematic literature review. Journal of the American Association of Nurse Practitioners. 2016;28(9):510-5. doi: 10.1002/2327-6924.12358. PubMed PMID: WOS:000385610900008.

555. Torjesen I. E-cigarettes are a "gateway from smoking," RCP concludes. BMJ. 2016;353:i2392. Epub 2016/04/30. doi: 10.1136/bmj.i2392. PubMed PMID: 27125216.

556. Trtchounian A, Talbot P. Electronic nicotine delivery systems: Is there a need for regulation? Tobacco Control. 2011;20(1):47-52. doi: 10.1136/tc.2010.037259. PubMed PMID: 2011-13551-008.

557. Trtchounian A, Williams M, Talbot P. Conventional and electronic cigarettes (e-cigarettes) have different smoking characteristics. Nicotine & Tobacco Research. 2010;12(9):905-12. doi: 10.1093/ntr/ntq114. PubMed PMID: WOS:000281346000005.

558. Trumbo CW, Harper R. Use and perception of electronic cigarettes among college students. Journal of American College Health. 2013;61(3):149-55. doi: 10.1080/07448481.2013.776052. PubMed PMID: 2013-12475-003.

559. Trumbo CW, Harper R. Perceived Characteristics of E-cigarettes as an Innovation by Young Adults. Health Behavior And Policy Review. 2015;2(2):154-62. PubMed PMID: 25729752.

560. Trumbo CW, Kim S-JS. The effect of electronic cigarette advertising on intended use among college students. Addictive Behaviors. 2015;46:77-81. doi: 10.1016/j.addbeh.2015.03.005. PubMed PMID: WOS:000353860300014.

561. Tuchman AE. Advertising and Demand for Addictive Goods: The Effects of E-Cigarette Advertising. working paper, 2016.

562. Twyman L, Bonevski B, Paul C, Bryant J, Gartner C, Guillaumier A. Electronic Cigarettes: Awareness, Recent Use, and Attitudes Within a Sample of Socioeconomically Disadvantaged Australian Smokers. Nicotine & Tobacco Research. 2016;18(5):670-7. doi: 10.1093/ntr/ntv183. PubMed PMID: WOS:000376350700023.

563. Unger JB, Barker D, Baezconde-Garbanati L, Soto DW, Sussman S. Support for electronic cigarette regulations among California voters. Tobacco Control. 2017;26(3):334-7. Epub 2016/05/22. doi: 10.1136/tobaccocontrol-2016-052918. PubMed PMID: 27207853.

564. Unger JB, Soto DW, Leventhal A. E-cigarette use and subsequent cigarette and marijuana use among Hispanic young adults. Drug and Alcohol Dependence. 2016;163:261-4. doi: 10.1016/j.drugalcdep.2016.04.027. PubMed PMID: 2016-21947-001.

565. Urrutia-Pereira M, Oliano VJ, Aranda CS, Mallol J, Sole D. Prevalence and factors associated with smoking among adolescents. Jornal De Pediatria. 2017;93(3):230-7. doi: 10.1016/j.jped.2016.07.003. PubMed PMID: WOS:000402692700005.

566. Valero-Juan LF, Suarez del Arco JA. [Knowledge, attitudes and perceptions of medical students about the electronic cigarette]. Atencion primaria. 2014;46(9):520-1. Epub 2014/08/12. doi: 10.1016/j.aprim.2014.03.004. PubMed PMID: 25107675.

567. van der Tempel J, Noormohamed A, Schwartz R, Norman C, Malas M, Zawertailo L. Vape, quit, tweet? Electronic cigarettes and smoking cessation on Twitter. International Journal of Public Health. 2016;61(2):249-56. doi: 10.1007/s00038-016-0791-2. PubMed PMID: WOS:000373180100014.

568. Vansickel AR, Eissenberg T. Electronic cigarettes: Effective nicotine delivery after acute administration. Nicotine & Tobacco Research. 2013;15(1):267-70. doi: 10.1093/ntr/ntr316. PubMed PMID: 2013-09839-034.

569. Vardavas CI, Filippidis FT, Agaku IT. Determinants and prevalence of e-cigarette use throughout the European Union: a secondary analysis of 26 566 youth and adults from 27 Countries. Tobacco Control. 2015;24(5):442-8. doi: 10.1136/tobaccocontrol-2013-051394. PubMed PMID: 109836439. Language: English. Entry Date: 20150911. Revision Date: 20160229. Publication Type: Journal Article.

570. Varlet V, Farsalinos K, Augsburger M, Thomas A, Etter JF. Toxicity assessment of refill liquids for electronic cigarettes. International Journal of Environmental Research and Public Health. 2015;12(5):4796-815. Epub 2015/05/06. doi: 10.3390/ijerph120504796. PubMed PMID: 25941845; PubMed Central PMCID: PMCPMC4454939.

571. Vasiljevic M, Petrescu DC, Marteau TM. Impact of advertisements promoting candy-like flavoured e-cigarettes on appeal of tobacco smoking among children: an experimental study. Tobacco control. 2016:tobaccocontrol-2015-052593.

572. Veliz P, McCabe SE, McCabe VV, Boyd CJ. Adolescent sports participation, e-cigarette use, and cigarette smoking. American Journal of Preventive Medicine. 2017;53(5):e175-e83. doi: 10.1016/j.amepre.2017.06.032. PubMed PMID: 2017-47482-028.

573. Venkatesan P. E-cigarette use in young people in the UK. Lancet Oncology. 2017;18(10):E571-E. doi: 10.1016/s1470-2045(17)30674-5. PubMed PMID: WOS:000411843500021.

574. Vickerman KA, Beebe LA, Schauer GL, Magnusson B, King BA. Electronic nicotine delivery system (ENDS) use during smoking cessation: a qualitative study of 40 Oklahoma quitline callers. BMJ open. 2017;7(4):e013079. Epub 2017/04/04. doi: 10.1136/bmjopen-2016-013079. PubMed PMID: 28365587.

575. Vickerman KA, Schauer GL, Malarcher AM, Zhang L, Mowery P, Nash CM. Reasons for Electronic Nicotine Delivery System use and smoking abstinence at 6 months: a descriptive study of callers to employer and health plan-sponsored quitlines. Tobacco Control. 2017;26(2). doi: 10.1136/tobaccocontrol-2015-052734. PubMed PMID: WOS:000394533400009.

576. Villanti AC, Johnson AL, Ambrose BK, Cummings KM, Stanton CA, Rose SW, et al. Flavored Tobacco Product Use in Youth and Adults: Findings From the First Wave of the PATH Study (2013-2014). American Journal of Preventive Medicine. 2017;53(2):139-51. doi: 10.1016/j.amepre.2017.01.026. PubMed PMID: WOS:000405998800007.

577. Villanti AC, Pearson JL, Glasser AM, Johnson AL, Collins LK, Niaura RS, et al. Frequency of Youth E-Cigarette and Tobacco Use Patterns in the United States: Measurement Precision Is Critical to Inform Public Health. Nicotine & Tobacco Research. 2017;19(11):1345-50. doi: 10.1093/ntr/ntw388. PubMed PMID: WOS:000412164900013.

578. Villanti AC, Rath JM, Williams VF, Pearson JL, Richardson A, Abrams DB, et al. Impact of exposure to electronic cigarette advertising on susceptibility and trial of electronic cigarettes and cigarettes in US young adults: A randomized controlled trial. Nicotine & Tobacco Research. 2016;18(5):1331-9. doi: 10.1093/ntr/ntv235. PubMed PMID: 2016-40277-119.

579. Villanti AC, Richardson A, Vallone DM, Rath JM. Flavored tobacco product use among US young adults. American Journal of Preventive Medicine. 2013;44(4):388-91.

580. Voigt K. Smoking norms and the regulation of e-cigarettes. American Journal of Public Health. 2015;105(10):1967-72. doi: 10.2105/ajph.2015.302764. PubMed PMID: 2015-51043-001.

581. Volesky KD, Maki A, Scherf C, Watson LM, Cassol E, Villeneuve PJ. Characteristics of e-cigarette users and their perceptions of the benefits, harms and risks of e-cigarette use: survey results from a convenience sample in Ottawa, Canada. Health promotion and chronic disease prevention in Canada : research, policy and practice. 2016;36(7):130-8. Epub 2016/07/14. PubMed PMID: 27409988; PubMed Central PMCID: PMCPMC4962102.

582. Wackowski OA, Delnevo CD. Young adults’ risk perceptions of various tobacco products relative to cigarettes: Results from the National Young Adult Health Survey. Health Education & Behavior. 2016;43(3):328-36. doi: 10.1177/1090198115599988. PubMed PMID: 2016-24451-010.

583. Wackowski OA, Delnevo CD, Pearson JL. Switching to E-Cigarettes in the Event of a Menthol Cigarette Ban. Nicotine & Tobacco Research. 2015;17(10):1286-7. doi: 10.1093/ntr/ntv021. PubMed PMID: WOS:000363175500016.

584. Wada P, Lam CN, Burner E, Terp S, Menchine M, Arora S. EXPOSURE TO AND USE OF ELECTRONIC CIGARETTES: DOES LANGUAGE MATTER? Ethnicity & Disease. 2017;27(3):217-22. doi: 10.18865/ed.27.3.217. PubMed PMID: WOS:000405902500004.

585. Wadsworth E, Neale J, McNeill A, Hitchman SC. How and Why Do Smokers Start Using E-Cigarettes? Qualitative Study of Vapers in London, UK. International Journal of Environmental Research and Public Health. 2016;13(7). Epub 2016/07/05. doi: 10.3390/ijerph13070661. PubMed PMID: 27376312; PubMed Central PMCID: PMCPMC4962202.

586. Wagener TL, Siegel M, Borrelli B. Electronic cigarettes: Achieving a balanced perspective. Addiction. 2012;107(9):1545-8. doi: 10.1111/j.1360-0443.2012.03826.x. PubMed PMID: 2012-21050-004.

587. Wagner NJ, Camerota M, Propper C. Prevalence and Perceptions of Electronic Cigarette Use during Pregnancy. Maternal and Child Health Journal. 2017;21(8):1655-61. doi: 10.1007/s10995-016-2257-9. PubMed PMID: WOS:000405962400008.

588. Wagoner KG, Cornacchione J, Wiseman KD, Teal R, Moracco KE, Sutfin EL. E-cigarettes, Hookah Pens and Vapes: Adolescent and Young Adult Perceptions of Electronic Nicotine Delivery Systems. Nicotine & Tobacco Research. 2016;18(10):2006-12. doi: 10.1093/ntr/ntw095. PubMed PMID: WOS:000386201100012.

589. Wang B, King BA, Corey CG, Arrazola RA, Johnson SE. Awareness and use of non-conventional tobacco products among U.S. students, 2012. American Journal of Preventive Medicine. 2014;47(2, Suppl 1):S36-S52. doi: 10.1016/j.amepre.2014.05.003. PubMed PMID: 2014-31191-008.

590. Wang L, Zhan Y, Li Q, Zeng DD, Leischow SJ, Okamoto J. An Examination of Electronic Cigarette Content on Social Media: Analysis of E-Cigarette Flavor Content on Reddit. International Journal of Environmental Research and Public Health. 2015;12(11):14916-35. doi: 10.3390/ijerph121114916. PubMed PMID: WOS:000365645500084.

591. Wang M, Wang JW, Cao SS, Wang HQ, Hu RY. Cigarette Smoking and Electronic Cigarettes Use: A Meta-Analysis. International Journal of Environmental Research and Public Health. 2016;13(1). doi: 10.3390/ijerph13010120. PubMed PMID: WOS:000374186100062.

592. Wang MP, Ho SY, Leung LT, Lam TH. Electronic cigarette use and its association with smoking in Hong Kong Chinese adolescents. Addictive Behaviors. 2015;50:124-7. doi: 10.1016/j.addbeh.2015.06.037. PubMed PMID: WOS:000359167000021.

593. Wang MP, Ho SY, Leung LT, Lam TH. Electronic Cigarette Use and Respiratory Symptoms in Chinese Adolescents in Hong Kong. JAMA Pediatrics. 2016;170(1):89-91. Epub 2015/11/10. doi: 10.1001/jamapediatrics.2015.3024. PubMed PMID: 26551991.

594. Wang MP, Li WH, Wu Y, Lam TH, Chan SS. Electronic cigarette use is not associated with quitting of conventional cigarettes in youth smokers. Pediatric Research. 2017;82(1):14-8. doi: 10.1038/pr.2017.80. PubMed PMID: WOS:000406256000006.

595. Wang MP, Li WHC, Jiang N, Chu LY, Kwong A, Lai V, et al. E-cigarette awareness, perceptions and use among community-recruited smokers in Hong Kong. PLOS ONE. 2015;10(10). PubMed PMID: 2015-49345-001.

596. Wang Y, Wilson FA, Larson J, Chen L-W. The Use of E-Cigarettes Among US Immigrants: The 2014 National Health Interview Survey. Public Health Reports. 2016;131(4):605-13. doi: 10.1177/0033354916662220. PubMed PMID: WOS:000380182800016.

597. Wasowicz A, Feleszko W, Goniewicz ML. E-Cigarette use among children and young people: the need for regulation. Expert Review of Respiratory Medicine. 2015;9(5):507-9. doi: 10.1586/17476348.2015.1077120. PubMed PMID: WOS:000361328400002.

598. Waters EA, Mueller-Luckey G, Levault K, Jenkins WD. Perceived Harms and Social Norms in the Use of Electronic Cigarettes and Smokeless Tobacco. Journal of Health Communication. 2017;22(6):497-505. doi: 10.1080/10810730.2017.1311972. PubMed PMID: WOS:000401555400006.

599. Weaver SR, Kemp CB, Heath JW, Pechacek TF, Eriksen MP. Use of Nicotine in Electronic Nicotine and Non-Nicotine Delivery Systems by US Adults, 2015. Public Health Reports. 2017;132(5):545-8. doi: 10.1177/0033354917723597. PubMed PMID: WOS:000410282300004.

600. Weaver SR, Kim H, Glasser AM, Sutfin EL, Barrington-Trimis J, Payne TJ, et al. Establishing consensus on survey measures for electronic nicotine and non-nicotine delivery system use: Current challenges and considerations for researchers. Addictive Behaviors. 2017. doi: 10.1016/j.addbeh.2017.11.016. PubMed PMID: 2017-52810-001.

601. Weaver SR, Majeed BA, Pechacek TF, Nyman AL, Gregory KR, Eriksen MP. Use of electronic nicotine delivery systems and other tobacco products among USA adults, 2014: Results from a national survey. International Journal of Public Health. 2016;61(2):177-88. doi: 10.1007/s00038-015-0761-0. PubMed PMID: 2015-51891-001.

602. Webb Hooper M, Kolar SK. Racial/Ethnic Differences in Electronic Cigarette Use and Reasons for Use among Current and Former Smokers: Findings from a Community-Based Sample. International Journal of Environmental Research and Public Health. 2016;13(10). Epub 2016/10/19. doi: 10.3390/ijerph13101009. PubMed PMID: 27754449; PubMed Central PMCID: PMCPMC5086748.

603. Webb Hooper M, Kolar SK. Racial/ethnic differences in electronic cigarette knowledge, social norms, and risk perceptions among current and former smokers. Addictive Behaviors. 2017;67:86-91. doi: 10.1016/j.addbeh.2016.12.013. PubMed PMID: 2017-03082-016.

604. Werse B, Mueller D, Stoever H, Dichtl A, Graf N. The Use of Electronic Vaping Devices among Adolescents - Use Patterns in a Representative Sample from Frankfurt/Main. Suchttherapie. 2017;18(3):134-9. doi: 10.1055/s-0043-113855. PubMed PMID: WOS:000407379600011.

605. Westling E, Rusby JC, Crowley R, Light JM. Electronic Cigarette Use by Youth: Prevalence, Correlates, and Use Trajectories From Middle to High School. Journal of Adolescent Health. 2017;60(6):660-6. doi: 10.1016/j.jadohealth.2016.12.019. PubMed PMID: WOS:000405362200007.

606. White J, Li J, Newcombe R, Walton D. Tripling Use of Electronic Cigarettes Among New Zealand Adolescents Between 2012 and 2014. Journal of Adolescent Health. 2015;56(5):522-8. doi: 10.1016/j.jadohealth.2015.01.022. PubMed PMID: WOS:000353052200010.

607. Willemsen MC, Croes EA, Kotz D, van Schayck OC. [Electronic cigarettes: use, health risks, and effectiveness as a cessation method]. Nederlands tijdschrift voor geneeskunde. 2015;159:A9259. Epub 2015/08/27. PubMed PMID: 26306484.

608. Williams M, Ghai S, Talbot P. Disposable electronic cigarettes and electronic hookahs: Evaluation of performance. Nicotine & Tobacco Research. 2015;17(2):201-8. doi: 10.1093/ntr/ntu118. PubMed PMID: 2015-03033-014.

609. Williams RJ, Knight R. Insights in public health: Electronic cigarettes: marketing to Hawai'i's adolescents. Hawai'i journal of medicine & public health : a journal of Asia Pacific Medicine & Public Health. 2015;74(2):66-70. Epub 2015/03/11. PubMed PMID: 25755916; PubMed Central PMCID: PMCPMC4338570.

610. Williams RS. VapeCons: E-cigarette user conventions. Journal of Public Health Policy. 2015;36(4):440-51. doi: 10.1057/jphp.2015.31. PubMed PMID: 2015-52490-004.

611. Willis E, Haught MJ, Morris II DL. Up in Vapor: Exploring the Health Messages of E-Cigarette Advertisements. Health Communication. 2016:1-9.

612. Wills TA, Knight R, Sargent JD, Gibbons FX, Pagano I, Williams RJ. Longitudinal study of e-cigarette use and onset of cigarette smoking among high school students in Hawaii. Tobacco Control. 2017;26(1):34-9. doi: 10.1136/tobaccocontrol-2015-052705. PubMed PMID: WOS:000391439000013.

613. Wills TA, Sargent JD. Do E-cigarettes reduce smoking? Preventive Medicine. 2017;100:285-6. doi: 10.1016/j.ypmed.2017.04.030. PubMed PMID: WOS:000405677000041.

614. Wills TA, Sargent JD, Knight R, Pagano I, Gibbons FX. E-cigarette use and willingness to smoke: a sample of adolescent non-smokers. Tobacco Control. 2016;25(E1):E52-E9. doi: 10.1136/tobaccocontrol-2015-052349. PubMed PMID: WOS:000375221900011.

615. Wilson FA, Wang Y. Recent Findings on the Prevalence of E-Cigarette Use Among Adults in the U.S. American Journal of Preventive Medicine. 2017;52(3):385-90. doi: 10.1016/j.amepre.2016.10.029. PubMed PMID: WOS:000400434200020.

616. Winickoff JP, Winickoff SE. Potential Solutions to Electronic Cigarette Use Among Adolescents. Pediatrics. 2016;138(2). Epub 2016/07/13. doi: 10.1542/peds.2016-1502. PubMed PMID: 27401100.

617. Wise J. Children are three times as likely to try e-cigarettes as tobacco products, study finds. BMJ. 2014;349:g7508. Epub 2014/12/17. doi: 10.1136/bmj.g7508. PubMed PMID: 25501667.

618. Wong LP, Alias H, Mohammadi NA, Ghadimi A, Hoe VCW. E-Cigarette Users' Attitudes on the Banning of Sales of Nicotine E-Liquid, Its Implication on E-Cigarette Use Behaviours and Alternative Sources of Nicotine E-Liquid. Journal of Community Health. 2017;42(6):1225-32. doi: 10.1007/s10900-017-0374-z. PubMed PMID: WOS:000413972300020.

619. Wong LP, Mohamad Shakir SM, Alias H, Aghamohammadi N, Hoe VCW. Reasons for using electronic cigarettes and intentions to quit among electronic cigarette users in Malaysia. Journal of Community Health: The Publication for Health Promotion and Disease Prevention. 2016;41(6):1101-9. doi: 10.1007/s10900-016-0196-4. PubMed PMID: 2016-52673-001.

620. Xu Y, Guo Y, Liu K, Liu Z, Wang X. E-Cigarette Awareness, Use, and Harm Perception among Adults: A Meta-Analysis of Observational Studies. PLOS ONE. 2016;11(11):e0165938. Epub 2016/11/20. doi: 10.1371/journal.pone.0165938. PubMed PMID: 27861501; PubMed Central PMCID: PMCPMC5115669.

621. Yang W, Wilson FA, Larson J, Li-Wu C. The Use of E-Cigarettes Among U.S. Immigrants: The 2014 National Health Interview Survey. Public Health Reports. 2016;131(4):605-13. PubMed PMID: 116715740. Language: English. Entry Date: 20160714. Revision Date: 20160714. Publication Type: Article.

622. Yao T, Max W, Sung HY, Glantz SA, Goldberg RL, Wang JB, et al. Relationship between spending on electronic cigarettes, 30-day use, and disease symptoms among current adult cigarette smokers in the U.S. PLOS ONE. 2017;12(11):e0187399. Epub 2017/11/08. doi: 10.1371/journal.pone.0187399. PubMed PMID: 29112988; PubMed Central PMCID: PMCPMC5675454.

623. Yingst JM, Veldheer S, Hammett E, Hrabovsky S, Foulds J. A Method for Classifying User-Reported Electronic Cigarette Liquid Flavors. Nicotine & Tobacco Research. 2017;19(11):1381-5. doi: 10.1093/ntr/ntw383. PubMed PMID: WOS:000412164900018.

624. Yingst JM, Veldheer S, Hrabovsky S, Nichols TT, Wilson SJ, Foulds J. Factors associated with electronic cigarette users’ device preferences and transition from first generation to advanced generation devices. Nicotine & Tobacco Research. 2015;17(10):1242-6. doi: 10.1093/ntr/ntv052. PubMed PMID: 2015-42983-009.

625. Yong H-H, Borland R, Balmford J, McNeill A, Hitchman S, Driezen P, et al. Trends in E-cigarette awareness, trial, and use under the different regulatory environments of Australia and the United Kingdom. Nicotine & Tobacco Research. 2015;17(10):1203-11. doi: 10.1093/ntr/ntu231. PubMed PMID: 2015-42983-004.

626. Yu E, Lippert AM. Race/ethnicity modifies the association between school prevalence of e-cigarette use and student-level use: Results from the 2014 US National Youth Tobacco Survey. Health & Place. 2017;46:114-20. doi: 10.1016/j.healthplace.2017.05.003. PubMed PMID: WOS:000407404500015.

627. Zarobkiewicz MK, Wawryk-Gawda E, Wozniakowski MM, Slawinski MA, Jodlowska-Jedrych B. Tobacco smokers and electronic cigarettes users among Polish universities students. Roczniki Panstwowego Zakladu Higieny. 2016;67(1):75-80. Epub 2016/03/10. PubMed PMID: 26953585.

628. Zhan Y, Liu R, Li Q, Leischow SJ, Zeng DD. Identifying topics for e-cigarette user-generated contents: A case study from multiple social media platforms. Journal of Medical Internet Research. 2017;19(1). doi: 10.2196/jmir.5780. PubMed PMID: 2017-04948-015.

629. Zhang X, Pu J. E-cigarette use among US adolescents: secondhand smoke at home matters. International Journal of Public Health. 2016;61(2):209-13. doi: 10.1007/s00038-015-0784-6. PubMed PMID: WOS:000373180100009.

630. Zheng Y, Zhen C, Dench D, Nonnemaker JM. US Demand for Tobacco Products in a System Framework. Health Economics. 2017;26(8):1067-86. doi: 10.1002/hec.3384. PubMed PMID: WOS:000405295000008.

631. Zhong J, Cao S, Gong W, Fei F, Wang M. Electronic Cigarettes Use and Intention to Cigarette Smoking among Never-Smoking Adolescents and Young Adults: A Meta-Analysis. International Journal of Environmental Research and Public Health. 2016;13(5). doi: 10.3390/ijerph13050465. PubMed PMID: WOS:000377256900022.

632. Zhou S, Van Devanter N, Fenstermaker M, Cawkwell P, Sherman S, Weitzman M. A study of the use, knowledge, and beliefs about cigarettes and alternative tobacco products among students at one U.S. medical school. Academic Medicine. 2015;90(12):1713-9. PubMed PMID: 2015-53806-038.

633. Zhu S-H, Gamst A, Lee M, Cummins S, Yin L, Zoref L. The Use and Perception of Electronic Cigarettes and Snus among the US Population. PLOS ONE. 2013;8(10). doi: 10.1371/journal.pone.0079332. PubMed PMID: WOS:000326152300092.

634. Zhu S-H, Sun JY, Bonnevie E, Cummins SE, Gamst A, Yin L, et al. Four hundred and sixty brands of e-cigarettes and counting: implications for product regulation. Tobacco control. 2014;23(suppl 3):iii3-iii9.

635. Zhu S-H, Zhuang Y-L, Wong S, Cummins SE, Tedeschi GJ. E-cigarette use and associated changes in population smoking cessation: evidence from US current population surveys. BMJ. 2017;358. PubMed PMID: 2017-32745-001.

636. Zhuang Y-L, Cummins SE, Sun JY, Zhu S-H. Long-term e-cigarette use and smoking cessation: A longitudinal study with US population. Tobacco Control. 2016;25(Supp 1):90-5. doi: 10.1136/tobaccocontrol-2016-053096. PubMed PMID: 2016-60344-007.
